# Ursane Triterpenes and Norisoprenoids from *Anchusa italica* Retz. and Their Chemotaxonomic Significance

**DOI:** 10.3390/plants14091385

**Published:** 2025-05-03

**Authors:** Linchuang Shen, Bingchen Han, Zhiliang Ma, Xianju Huang, Guangzhong Yang, Yanfeng Zeng, Maochuan Liao, Ruixi Gao, Jun Li

**Affiliations:** 1 School of Pharmaceutical Sciences, South-Central Minzu University, Wuhan 430074, China2024110514@mail.scuec.edu.cn (Z.M.); 2023110427@mail.scuec.edu.cn (X.H.); 2023110457@mail.scuec.edu.cn (G.Y.); 2022110479@mail.scuec.edu.cn (Y.Z.); shenlinchuang1016@163.com (M.L.); 2 College of Life Sciences, South-Central Minzu University, Wuhan 430074, China; 2023010071@mail.scuec.edu.cn; 3 Qinghai Tibetan Medicine Research Institute, Xining 810016, China; 4 Science and Technology Cooperation Base for Evaluation and Utilization of Traditional Medical Resources, South-Central Minzu University, Wuhan 430074, China

**Keywords:** *Anchusa italica* Retz., structural elucidation, ursane triterpene, norisoprenoid, chemotaxonomic significance

## Abstract

A total of 31 compounds were isolated from the ethyl acetate and *n*-butanol fractions of *Anchusa italica* Retz., which contained one ursane triterpenoid, 2*α*,3*β*,19*α*-trihydroxy-23-formyl-urs-12-en-28,21*β-*olide (**1**), and five norisoprenoids: (2*R*,6*R*,9*S*)-9-hydroxy-4-megastigmen-3-one-2*-O-β-*D-glucopyranoside (**3**); (2*R*,6*S*,9*S*)-9-hydroxy-megastigman-4,7-dien-3-one-2*-O-β-*D-glucopyranoside (**4**); (+)-isololiolide *β-*D-glucopyranoside (**5**); (2*S*,8*R*)-loliolide *β-*D-glucopyranoside (**6a**); and (2*R*,8*S*)-loliolide *β-*D-glucopyranoside (**6b**). It also contained 25 known compounds (**2** and **7–30**). The chemical structures of the compounds, inclusive of their absolute configurations, were ascertained using spectroscopic methods such as NMR, HR-MS, and quantum chemical calculations (computational NMR and ECD), in combination with relevant literature data. Moreover, the chemotaxonomic significance of the isolated substances was discussed, with compounds **1**, **2**, and **7–13** potentially broadening the application of triterpenes as taxonomic markers for the classification of the genus *Anchusa*.

## 1. Introduction

*Anchusa italica* Retz. belongs to the genus *Anchusa* (Boraginaceae family) and is a perennial herbaceous plant that is naturally distributed in the Mediterranean region and tropical areas. In China, it is found in the northwestern and southwestern regions, such as Xinjiang, Gansu, and Sichuan [1]. As an important medicinal resource in the Uyghur medicine system, *A. italica* is recorded as “Gao Zi Wan” (meaning “tough”) and often used in the clinical treatment of cardiovascular and cerebrovascular diseases such as hypertension, palpitations, coughs, and depression [2]. Chemical investigations into *A. italica* have revealed that this plant contains abundant natural products, such as essential oils, triterpenoids, flavonoids, steroids, and alkaloids. From the perspective of pharmacological effects, the extracts and active compounds of *A. italica* exhibit a wide range of biological activities, including cardiovascular protection and anti-inflammatory, anti-bacterial, anti-oxidative, and neuroprotective effects [3]. To date, research has mainly focused on isolating and identifying total flavonoids and triterpenoid components, as well as exploring the mechanisms by which they protect myocardial cells [4]. Systematic analyses of their composition, activity, and contribution to plant taxonomy remain lacking.

**Figure 1:**
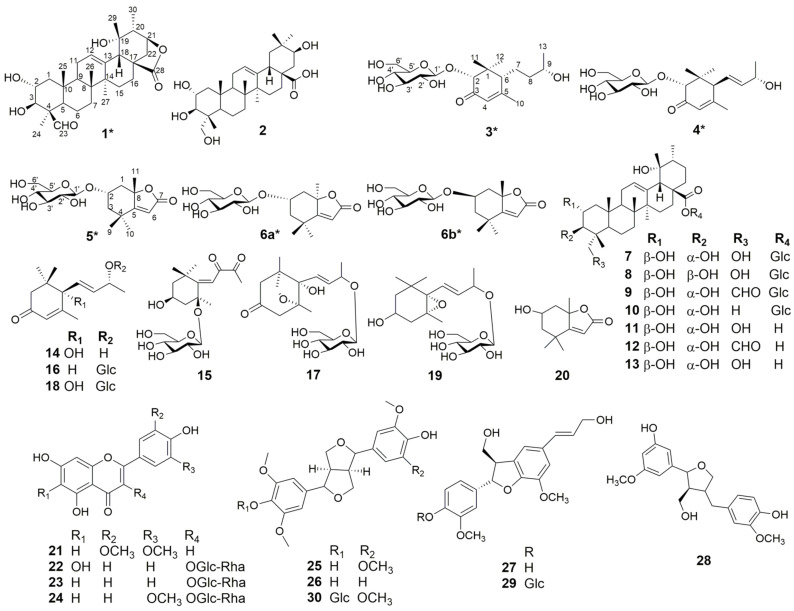
Chemical structures of compounds **1–30** (* for the new compounds).

To explore more compounds with biological activities and their contribution to the chemotaxonomic significance of this plant, we provide a certain basis for the development and utilization of *A. italica*. The whole plant powder was refluxed with 70% ethanol and then sequentially extracted with petroleum ether (PE), dichloromethane (CH_2_Cl_2_), ethyl acetate (EtOAc), and *n*-butanol (BuOH). The EtOAc and BuOH fractions were separated using chromatographic separation techniques: normal, reversed-phase silica gel column chromatography (CC), and HPLC. A total of thirty-one compounds (including nine triterpenes, twelve sesquiterpenes, four flavonoids, and six lignans) were identified, with six new compounds (**1**, **3**, **4**, **5**, **6a**, and **6b**) as well as fourteen known compounds (**15**, **16**, **18–25**, and **27–30**) first reported from *A. italica* (Figure 1). Compounds **5**, **6a**, and **6b** are a differential isomer and a pair of stereoisomers, and in this study, the absolute configurations of these three compounds were identified for the first time using DP4+ analysis and ECD calculations. The chemotaxonomic significance of the isolated substances is also discussed.

## 2. Results

### 2.1. Structure Elucidation

Chemical investigation of the aerial parts of *A. italica* yielded six novel compounds (**1**, **3–5**, **6a**, and **6b**) and 25 known analogues (**2** and **7**–**30**), including 2*α*,3*β*,19*α*-trihydroxy- 23-formyl-urs-12-en-28,21*β-*olide (**1**); (2*R*,6*R*,9*S*)-9-hydroxy-4-megastigmen-3-one-2*-O- β-*D-glucopyranoside (**3**); (2*R*,6*S*,9*S*)-9-hydroxy-megastigman-4,7-dien-3-one-2*-O-β-*D- glucopyranoside (**4**); (+)-isololiolide *β-*D-glucopyranoside (**5**); (2*S*,8*R*)-loliolide *β-*D- glucopyranoside (**6a**); (2*R*,8*S*)-loliolide *β-*D-glucopyranoside (**6b**); 2α,3*β*,21,24- tetrahydroxyoleanan-12-en-28-oic acid (**2**); niga-ichigoside F1 (**7**); niga-ichigoside F2 (**8**); pinfaensin (**9**); glucosyl tormentate (**10**); myrianthic acid (**11**); 24-*epi*-pinfaensic acid (**12**); hydroxyasiatic acid (**13**); (+)-vomifoliol (**14**) [3,4]; lippianoside E (**15**) [5]; 3-oxo-*α*- ionol-*β*-D-glucopyranoside (**16**) [6]; asysgangoside (**17**) [4]; (6*S*, 9*R*)-roseoside (**18**) [7]; sammangaoside B (**19**) [8]; (+)-isololiolide (**20**) [9]; tricin (**21**) [10]; 6-hydroxykaempferol 3-*β*-rutinoside (**22**) [11]; kaempferol 3-*O*-rutinoside (**23**) [12]; narcissin (**24**) [13]; (+)-syringaresinol (**25**) [14]; (+)-mediaresinol (**26**) [4]; dehydrodiconiferyl alcohol (**27**) [15]; vibruresinol (**28**) [16]; dehydrodiconiferyl alcohol 4-*O*-*β*-D-glucopyranoside (**29**) [17]; and syringaresinol-4′-*O*-*β*-D-glucopyranoside (**30**) [14].

Compound **1** was obtained as yellow amorphous powder with a molecular formula of C_30_H_44_O_6_ deduced from *m*/*z* 501.3211 [M + H]^+^, with an unsaturation degree of 9. The 1D NMR data (Table 1) revealed the presence of six tertiary methyl groups at [*δ*_H_ 0.90 (CH_3_-25), 0.95 (CH_3_-26), 1.05 (3H, d, *J* = 7 Hz, CH_3_-29), 1.21 (CH_3_-30), 1.26 (CH_3_-24), and 1.39 (CH_3_-27); *δ*_C_ 18.0, 17.4, 13.8, 28.4, 21.4, and 26.2, respectively]; three oxymethine protons at [*δ*_H_ 3.11 (1H, d, *J* = 9.5 Hz, H-3), 4.06 (1H, ddd, *J* = 5, 7, 10 Hz, H-2), and 4.34 (1H, d, *J* = 5.5 Hz, H-21); *δ*_C_ 82.6, 69.2, and 83.9]; one olefinic proton at [*δ*_H_ 5.53 (1H, t, *J* = 4 Hz, H-12); *δ*_C_ 131.3 and 137.7]; one aldehyde group at [*δ*_H_ 9.90 (1H, s, H-23); *δ*_C_ 208.5]; and one ester carbonyl group at (*δ*_C_ 185.4), suggesting that it is an ursane derivative. Its NMR data (Table 1) showed a strong similarity to 2α,3*β*,19α,23-tetrahydroxyurs-12-en-28, 21*β*-olide [3], except that the chemical shift of C-23 changed from *δ*_C_ 66.4 to *δ*_C_ 208.5 in compound **1**, indicating that the C-23 changed from an oxymethylene to an aldehyde group.

**Figure 2:**
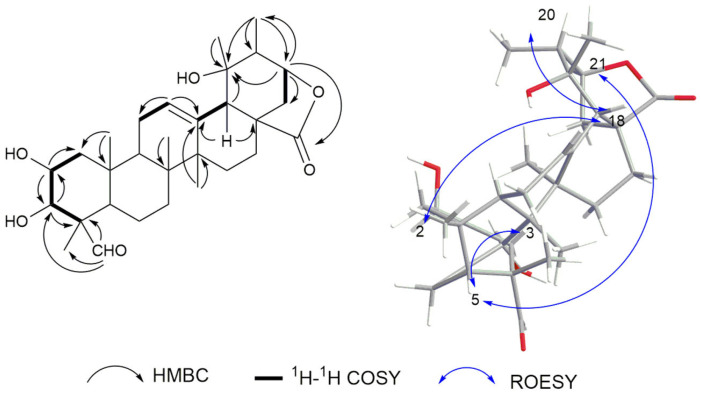
Key ^1^H-^1^H COSY (black bold), HMBC (black arrow) and ROESY (blue arrow) correlations for **1**.

The following correlations helped to confirm the presence of the ursolic acid skeleton: HMBC correlations of H-12 (*δ*_H_ 5.53) with C-11 and C-13 (*δ*_C_ 25.4 and 137.7); H-18 (*δ*_H_ 2.40) with C-13, C-17, and C-19 (*δ*_C_ 137.7, 45.8, and 74.3). Moreover, the following HMBC correlations helped to confirm the presence of the γ-lactone unit: correlations of H-21 (*δ*_H_ 4.35) with C-19, C-22, C-28, and C-30 (*δ*_C_ 74.3, 34.0, 185.4, and 28.4, respectively), together with the unsaturation degree of 9. Other correlations were also found, like H-23 (*δ*_H_ 9.90) with C-4 and C-24 (*δ*_C_ 55.3 and 21.4); CH_3_-25 (*δ*_H_ 0.90) with C-1 and C-10 (*δ*_C_ 47.5 and 39.3); CH_3_-27 (*δ*_H_ 1.39) with C-13 and C-14 (*δ*_C_ 137.7 and 42.7); and CH_3_-30 (*δ*_H_ 2.40) with C-19, C-20, and C-21 (*δ*_C_ 74.3, 45.3, and 83.9). Based on biosynthesis considerations, the natural ursane skeleton has an α-orientation for H-5 and a *β*-orientation for H-18 and CH_3_-25. The ROESY correlations of H-2 (*δ*_H_ 4.06) with H-18 (*δ*_H_ 2.40) and H-20 (*δ*_H_ 1.79), and H-5 (*δ*_H_ 1.15) with H-3 and H-21 (*δ*_H_ 3.11 and 4.34) suggested that H-2 is *β*-oriented, while H-3 and H-21 are α-oriented (Figure 2). Taking these data together, compound **1** was identified as 2*α*,3*β*,19*α*-trihydroxy-23-formyl-urs-12-en-28,21*β-*olide.

Compound **3** was obtained as a colorless oil, possessing a molecular formula of C_19_H_32_O_8_ deduced from *m*/*z* 389.2170 [M + H]^+^ as well as the unsaturation degree of 4. The 1D NMR data (Table 2 and Table 3) revealed the presence of four methyl groups at *δ*_H_ 2.03 (*δ*_C_ 24.6, CH_3_-10), 1.19 (*δ*_C_ 21.0, CH_3_-11), 1.19 (*δ*_C_ 19.8, CH_3_-13) and 0.88 (*δ*_C_ 24.5, CH_3_-12); one olefinic proton at [*δ*_H_ 5.81 (1H, d, *J* = 1.0 Hz, H-4) and *δ*_C_ 124.0]; a ketocarbonyl (*δ*_C_ 201.2) forming a conjugation with the olefinic bond; an isomeric proton at *δ*_H_ 4.20 (1H, s, H-2); and an oxygenated methyl proton at *δ*_H_ 3.87 (1H, m, H-9). Chemical shifts in the *δ*_H_ 3.23–3.90, along with an anomeric proton resonating at [*δ*_H_ 4.33 (1H, d, *J* = 8.0 Hz, H-1′), *δ*_C_ 102.0], were assigned as part of a sugar moiety and matched well with previously reported data as a *β*-glucopyranose. The ^1^H and ^13^C NMR data (Table 2 and Table 3) for **3** were similar to those for (2*R*,6*R*,9*R*)-2,9-dihydroxy-4- megastigmen-3-one [18]. Only one more glucose unit is present in **3**, and the chemical shift value of C-2 was down-field shifted from *δ*_C_ 76.0 (in (2*R*,6*R*,9*R*)-2,9-dihydroxy- 4-megastigmen-3-one) to *δ*_C_ 77.2 in **3**. HMBC correlations were analyzed from CH_3_-11 (*δ*_H_ 1.19) to C-1, C-2, C-6, and C-12 (*δ*_C_ 43.2, 77.2, 54.5, and 24.5, respectively); from H-10 (*δ*_H_ 2.03) to C-4, C-5, and C-6 (*δ*_C_ 124.0, 168.6, and 54.5); from H-7 (*δ*_H_ 2.11) and H-8 (*δ*_H_ 1.60) to C-6 (*δ*_C_ 54.5); and from H-13 (*δ*_H_ 1.19) to C-8 and C-9 (*δ*_C_ 38.2 and 75.4). Glucose was attributable to C-2 by HMBC correlations from *δ*_H_ 4.33 (H-1′) to *δ*_C_ 77.2 (C-2). The relative configuration of **3** was conducted to find correlations among H-2 (*δ*_H_ 4.20), H-6 (*δ*_H_ 2.10) and CH_3_-11 (*δ*_H_ 0.88), providing *β*-oriented configurations. 

**Figure 3:**
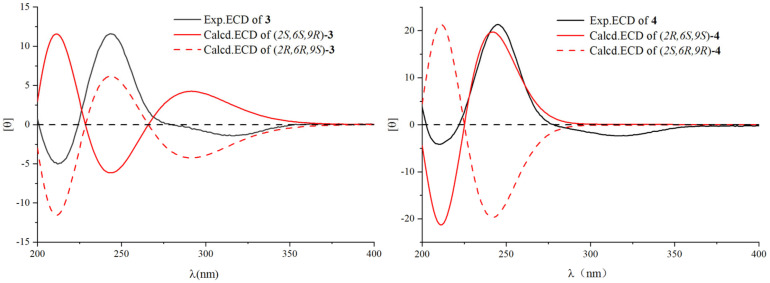
Experimental and calculated ECD spectra for **3** and **4**.

To determine the absolute configuration of the sugar molecule of compound **3**, acid hydrolysis followed by chemical derivatization was performed to determine the D/L configuration of *β*-glucopyranose (Figures S59 and S64). The *β*-glucopyranose from the acid hydrolysis of compound **3** and the standard *β*-D glucopyranose were chemically reacted to give the final product. Their retention times were compared by HPLC analysis, which showed that the *β*-glucopyranose in compound **3** was identified as the D-form, as they showed the same retention time (*t_R_* = 20.2 min). To further verify the configuration of HO-9, two isomers (2*R*,6*R*,9*S*) and (2*R*,6*R*,9*R*) of **3** were subjected to NMR calculations with the DP4+ analysis. These results showed that (2*R*,6*R*,9*S*) of **3** (Figure S56) was the most likely structure for **3,** with a 100.00% DP4+ probability based on all of the NMR data. The theoretical calculations of ECD spectra for **3** (2*S*,6*S*,9*R*) and the enantiomer (2*R*,6*R*,9*S*) were also performed using the TDDFT method. The calculated ECD spectra (2*R*,6*R*,9*S*) were in agreement with the experimental data (a positive peak at λ_250_ nm and a negative peak at λ_300_ nm), which indicated the absolute configuration (Figure 3). Taking these data together, compound **3** was identified as (2*R*,6*R*,9*S*)-9-hydroxy-4- megastigmen-3-one-2-*O*-*β*-D-glucopyranoside.

Compound **4** is a white powder, possessing a molecular formula of C_19_H_30_O_8_ deduced from *m*/*z* 387.20135 [M + H]^+^. The unsaturation degree is 5. The 1D NMR data (Table 2 and Table 3) suggested compound **4** might be the glucoside derivative at C-2 of the known compound 2,9-dihydroxy-megastigman-4,7-dien-3-one, because the chemical shift value of C-2 was down-field shifted from *δ*_C_ 75.5 in 2,9-dihydroxy-megastigman- 4,7-dien-3-one to *δ*_C_ 77.4 in **4** [19]. Glucose was attributable to C-2 by HMBC correlations from H-1′ (*δ*_H_ 4.36) to C-2 (*δ*_C_ 77.4). The *β*-glucopyranose in **4** was identified as the D-form, as they showed the same retention time (*t_R_* = 20.2 min) in HPLC analysis. The relative configuration was assigned by ROESY correlations of H-2 (*δ*_H_ 4.17) with H-8 (*δ*_H_ 5.77), H-9 (*δ*_H_ 4.40), and CH_3_-11 (*δ*_H_ 0.90), suggesting that H-2, H-9, and CH_3_-11 were on the same side of the molecule. The absolute configuration of **4** was determined via ECD calculations using the TDDFT method. The calculated ECD data for (2*R*,6*S*,9*S*) were in agreement with the experimental data of 4 (Figure 3), which indicated that compound **4** could be identified as (2*R*,6*S*,9*S*)-9-hydroxy-megastigman-4,7-dien-3-one-2-O-β-D- glucopyranoside.

**Figure 4:**
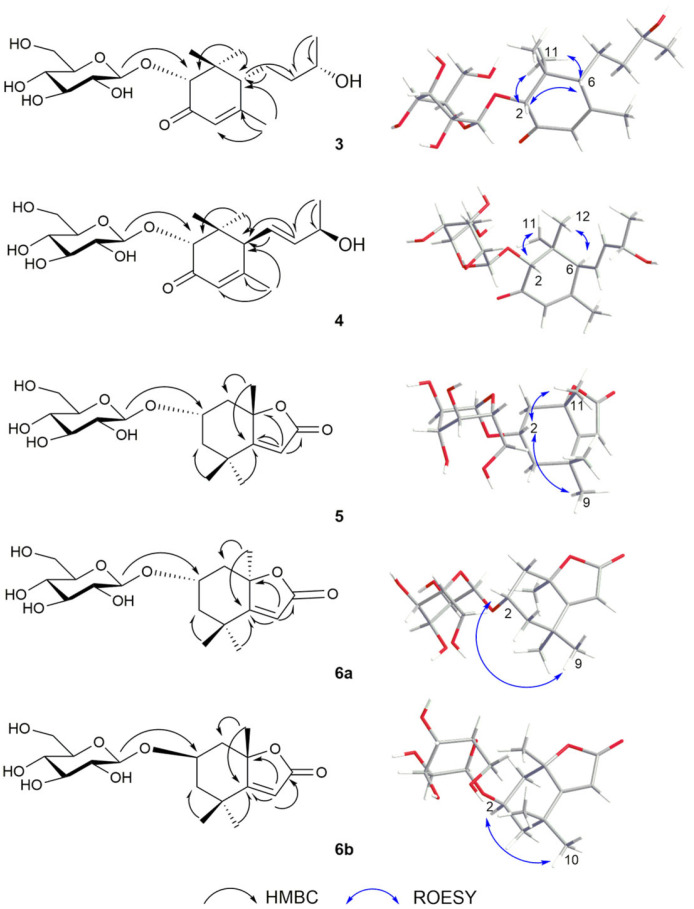
Key HMBC (black arrow) and ROSEY (blue arrow) correlations for **3–5**, **6a**, and **6b**.

**Figure 5:**
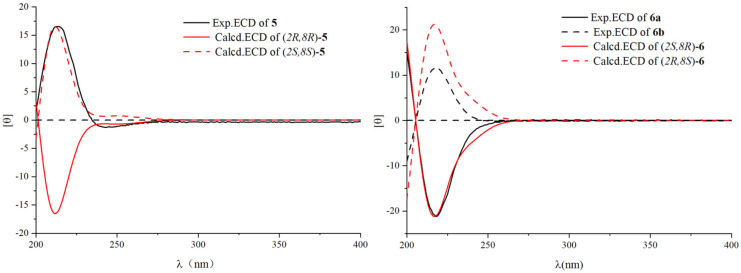
Experimental and calculated ECD spectra for **5**, **6a**, and **6b**.

Compound **5** was obtained as a colorless oil, possessing a molecular formula of C_17_H_26_O_8_ deduced from *m*/*z* 381.1519 [M + Na]^+^ and ^13^C NMR data, as well as an unsaturation degree of 5. The 1D NMR data (Table 2 and Table 3) revealed the presence of three methyl groups at [(*δ*_H_ 1.59 (CH_3_-11), 1.31 (CH_3_-10) and 1.28 (CH_3_-9); *δ*_C_ 25.4, 30.1, and 25.1)]; one olefinic proton at [*δ*_H_ 5.77 (1H, s, C-6), *δ*_C_ 113.4]; one ester carbonyl at (*δ*_C_ 173.8); and an isomeric proton at *δ*_H_ 4.27 (1H, m, H-2). Chemical shifts from *δ*_H_ 3.10 to 3.92, along with an anomeric proton resonating at [*δ*_H_ 4.42 (1H, d, *J* = 8.0 Hz, H-1′), *δ*_C_ 102.5], were assigned as part of a sugar moiety, which matched well with previously reported data, and subsequently confirmed as *β*-D-glucopyranose by HPLC (*t_R_* = 20.2 min, Figures S66 and S67). The ^1^H and ^13^C NMR data (Table 2 and Table 3) of **5** were similar to those of a known compound, (6*S*,7a*S*)-6-hydroxy-4,4,7a-trimethyl-6,7-dihydro-5*H*-1-benzofuran-2-one [20]. Only one more glucose unit appeared in **5,** with the chemical shift value from *δ*_C_ 63.9 of (6S,7aS)-6-hydroxy-4,4,7a-trimethyl-6,7-dihydro-5*H*-1-benzofuran-2-one to *δ*_C_ 72.9 of **5**. HMBC correlations were observed among H-9 (*δ*_H_ 1.28) with C-3, C-4, C-5, and C-10 (*δ*_C_ 48.9, 35.9, 183.8 and 30.1, respectively); H-11 (*δ*_H_ 1.59) with C-1, C-5, and C-8 (*δ*_C_ 45.6, 183.8, and 88.3); and H-6 (*δ*_H_ 5.77) to C-4, C-5, C-7, and C-8 (*δ*_C_ 35.9, 183.8, 173.8 and 88.3). Glucose was assigned at C-2 by the HMBC correlation (Figure 4) of H-1′ (*δ*_H_ 4.42) with C-2 (*δ*_C_ 72.9). The ROESY data of compound **5** indicated that H-2 was α-oriented by correlations of *δ*_H_ 4.27 (H-2) with *δ*_H_ 1.31 (CH_3_-9) and *δ*_H_ 1.59 (CH_3_-11). At last, the ECD analysis of **5** (2*R*,8*R*) and the enantiomer (2*S*,8*S*) were performed using the TDDFT method. The calculated ECD spectra for (2*S*,8*S*) were in agreement with the experimental spectra of **5** (Figure 5). Taking these data together, compound **5** was identified as (2*S*,8*S*)-isololiolide *β*-D-glucopyranoside.

Due to the similar 1D and part of 2D NMR data (HSQC, HMBC) of compounds **6a**, **6b,** and **5** (Table 2 and Table 3), the configuration of **6a** and **6b** was analyzed by ROESY experiment, and further verified by ECD calculations performed by the TDDFT method. ROESY results of stereoisomers **6a** and **6b** suggested that the H-2 (*δ*_H_ 4.28) of **6a** was correlated with CH_3_-9 (*δ*_H_ 1.27), while that of **6b** was correlated with CH_3_-10 (*δ*_H_ 1.45). At the same time, no correlations were found between H-2 (*δ*_H_ 4.28 and 4.30) and CH_3_-11 (*δ*_H_ 1.75 and 1.74) of both compounds. In addition, the calculated ECD spectra for (2*S*,8*R*) were in agreement with the experimental data of **6a**, and the same spectra for (2*R*,8*S*) with the experimental data of **6b** (Figure 5). In general, compound **6a** was identified as (2*S*,8*R*)-loliolide *β*-D-glucopyranoside and compound **6b** was identified as (2*R*,8*S*)-loliolide *β*-*D*-glucopyranoside.

### 2.2. The Protective Effects of Compounds 1–20 on Hypoxia/Reoxygenation-Injured Neonatal Rat Cardiomyocytes

An in vitro experimental model of hypoxia/reoxygenation (H/R) injury in neonatal rat cardiomyocytes was developed to evaluate the protective effects of isolated compounds **1–20**. Neonatal rat cardiomyocytes were subjected to H/R injury for four hours and subsequently evaluated for the potential protective effects of compounds **1–20**. The survival of H/R-treated neonatal rat cardiomyocytes was slightly increased after treatment with compounds **2**, **4**, **6b**, **7**, **9**, **17**, and **20** compared to the control group (H/R treatment only), suggesting that those compounds may have a protective effect on H/R-injured neonatal rat cardiomyocytes (Figure S65).

### 2.3. Chemotaxonomic Significance

In the present study, 31 compounds were identified from the aerial part of *A. italica* (Table 4). They were categorized as triterpenes (**1**, **2**, and **7–13**), norisoprenoids (**3–6** and **14–20**), flavonoids (**21–24**), and lignans (**25–30**). Among these compounds, three classes of compounds including norisoprenoids, flavonoids, and lignan derivatives were isolated from *A. italica*, which have been reported in other *Anchusa* species and even some species in the Boraginaceae family (Table 4). Compounds **18**, **23**, **24**, **25**, and **30** were reported in *Borago officinalis* L. [7], *A. strigose* Banks and Sol. [12], *Heliotropium angiospermum* [13], and *Moltkia aurea* Boiss. [14], leading to a close relationship from the perspective of chemical composition. Compounds **15**, **16**, **18–25**, and **27–30** were found for the first time in *A. italica*, which may complement the results from existing investigations. In previous studies, triterpenes isolated from *A. italica* were rarely identified; thus, compounds **1**, **2**, and **7–13** might expand the use of triterpenes, especially ursane triterpenoids, as chemical taxonomic markers for the classification of the genus *Anchusa*.

## 3. Materials and Methods

### 3.1. General Experimental Procedures

NMR spectra were obtained on a Bruker AM-500 MHz spectrometer (Bruker, Karlsruher, Germany) using TMS as an internal reference. HR-ESI-MS was performed on a quadrupole ion trap high-resolution mass spectrometer (ThermoFisher Scientific, Waltham, MA, USA). CD spectra were obtained using a Chirascan detector (Applied Photophysics Limited Shanghai Representative Office, Shanghai, China). Optical rotation values were recorded on a Rudolph Auto pol IV polarimeter (Rudolph, Hackettstown, NJ, USA). UV full-wavelength scanning was conducted on a Shimadzu UV-2401 PC spectrophotometer (Shimadzu, Kyoto, Japan). CC was performed using silica gel (200–300 mesh, 300–400 mesh, Qingdao Haiyang Chemical Group Co., Qingdao, China). Thin-layer chromatography was performed on silica gel GF254 (Qingdao Haiyang Chemical Group Co., China). D101 macroporous resin was purchased from Tianjin Yunkai Company, China. MCI gel was purchased from Mitsubishi Chemical Group Co. (Mitsubishi Chemical Group, Toyko, Japan). Semi-preparative HPLC was performed on a QingBoHua HPLC system (QingBoHua Technology, Beijing, China) equipped with a binary high-pressure pump, a dual–wavelength UV detector, and a C18 column (250 mm × 10 mm, 5 μm, YMC Co., Ltd., Kyoto, Japan). Other chemical reagents were purchased from Sinopharm Chemical Reagent Co., Ltd., Shanghai, China.

The ECD calculations were carried out using Gaussian 09. Systematic conformational analyses for compounds **3**, **4**, **5**, **6a,** and **6b** were performed via Confab using the MMFF94 molecular mechanics force field calculation. All of these conformers were further optimized using time-dependent density functional theory (TDDFT) at the B3LYP/6–311G (d, p) level in methanol with the IEFPCM model. Vibrational frequency analysis confirmed the stable structures. Based on the optimized structures, the ECD calculation was conducted using TDDFT with a total of 60 excited states. The ECD spectrum was simulated in SpecDis using a Gaussian function with half–bandwidths of 0.3 eV for compounds **3**, **4**, and **5**.

### 3.2. Plant Material

The whole plant of *A. italica* was collected from Xinjiang Uygur Autonomous Region. The plant was picked and identified as *A. italica* by researcher Shi Leiling of the Xinjiang Institute of Traditional Chinese Medicine and Ethnomedicine. The specimen (A20220907) is stored in the Museum of Traditional Chinese Medicine, College of Traditional Chinese Medicine, Xinjiang Medical University.

### 3.3. Extraction and Isolation

The powder (2.5 kg) of *A*. *italica* was extracted in 70% ethanol under reflux (3 × 2 h each time). The ethanol extract was evaporated under reduced pressure to obtain the total extract (356 g). It was dissolved in water and then extracted three times sequentially with PE, CH_2_Cl_2_, EtOAc, and BuOH to provide the fractions accordingly.

The ethyl acetate soluble fraction (21 g) was subjected to CC (9.4 × 40 cm) over silica gel (200–300 mesh) and eluted with CH_2_Cl_2_/MeOH (100:0–70:30, *v/v*) to yield three fractions (Y1–3). Y1 (6 g) was subjected to CC (3 × 60 cm) over silica gel (300–400 mesh) and eluted with PE-EtOAc (85:15, *v/v*) to yield two fractions (Y11–12). Y11 (150 mg) was prepared by HPLC using acetonitrile (MeCN)/H_2_O (35:65, 210/254 nm) to provide compounds **20** (*t_R_* = 20 min, 3 mg), **21** (*t_R_* = 23 min, 2.6 mg), and **14** (*t_R_* = 27 min, 2.5 mg). Y12 (200 mg) was prepared by HPLC using MeCN/H_2_O (30:70, 210/254 nm) to provide compounds **15** (*t_R_* = 14 min, 3 mg), **25** (*t_R_* = 18 min, 13 mg), **26** (*t_R_* = 22 min, 3 mg), and **1** (*t_R_* = 29 min, 7 mg). Y2 (3.8 g) was subjected to CC (3 × 60 cm) over silica gel (300–400 mesh) and eluted with CH_2_Cl_2_/MeOH (50:1–10:1, *v/v*) to yield two fractions (Y21–22). Y21 (100 mg) was prepared by HPLC using MeCN/H_2_O (25:75, 210/254 nm) to provide compounds **27** (*t_R_* = 16 min, 1.8 mg) and **28** (*t_R_* = 20 min, 7 mg). Y22 (700 mg) was prepared by HPLC using MeCN/H_2_O (20:80, 210/254 nm) to provide compounds **7** (*t_R_* = 16 min, 134 mg), **8** (*t_R_* = 23 min, 86 mg), and **9** (*t_R_* = 29 min, 128 mg). Y3 (350 mg) was prepared by HPLC using MeCN/H_2_O (18:82, 210/254 nm) to provide compounds **10** (*t_R_* = 20 min, 12.6 mg), **11** (*t_R_* = 36 min, 23.4 mg), **23** (*t_R_* = 43 min, 3.5 mg), and **24** (*t_R_* = 45 min, 3.6 mg).

The BuOH soluble fraction (45.87 g) was subjected to CC (9.4 × 40 cm) over silica gel (200–300 mesh) and eluted with CH_2_Cl_2_/MeOH (100:0–60:40, *v/v*) to yield two fractions (Z1-Z2). Z1 (3.86 g) was subjected to CC (3 × 60 cm) over silica gel (300–400 mesh) and eluted with PE-EtOAc (75:25, *v/v*) to yield two fractions (Z11-Z12). Z11 (160 mg) was prepared by HPLC using MeCN/H_2_O (20:80, 210/254 nm) to provide compounds **3** (*t_R_* = 15.5 min, 2.1 mg), **16** (*t_R_* = 32 min, 1.0 mg), and **12** (*t_R_* = 38 min, 7.3 mg). Z12 (230 mg) was prepared by HPLC using MeCN/H_2_O (17:83, 210/254 nm) to provide compounds **22** (*t_R_* = 36 min, 4 mg), **29** (*t_R_* = 39 min, 2.3 mg), and **30** (*t_R_* = 45 min, 10.7 mg). Z2 (2.5 g) was subjected to CC (3×60 cm) over silica gel (300–400 mesh) and eluted with CH_2_Cl_2_/MeOH (70:30, *v/v*) to yield three fractions (Z21-Z23). Z21 (300 mg) was prepared by HPLC using MeCN/H_2_O (15:85, 210/254 nm) to provide compounds **13** (*t_R_* = 17 min, 3.4 mg), **4** (t_R_ = 24 min, 0.8 mg), and **2** (*t_R_* = 29 min, 5.4 mg). Z22 (200 mg) was prepared by HPLC using MeCN/H_2_O (12:88, 210/254 nm) to provide compounds **17** (*t_R_* = 20 min, 23 mg), **18** (*t_R_* = 24 min, 6 mg), and **19** (*t_R_* = 36 min, 10.5 mg). Z23 (150 mg) was prepared by HPLC using MeCN/H_2_O (10:90, 210/254 nm) to provide compounds **5** (*t_R_* = 28 min, 1.3 mg), **6a** (t_R_ = 30 min, 0.8 mg), and **6b** (*t_R_* = 32 min, 0.8 mg).

Spectroscopic Data

2*α*,3*β*,19*α*-trihydroxy-23-formyl-urs-12-en-28,21*β-*olide (**1**): yellow amorphous powder; ${\left[\alpha \right]}_{D}^{20}$ = +70.4° (c 0.03, MeOH); UV (MeOH) λ_max_ 205 nm; ^1^H and ^13^C NMR data (CD_3_OD, 500 and 125 MHz), see Table 1; HR-ESI-MS *m*/*z* 501.3211 [M + H]^+^ (calcd for C_30_H_45_O_6_, 501.3200). Supplementary data were available at Figures S1–S10.

(2*R*,6*R*,9*S*)-9-hydroxy-4-megastigmen-3-one-2*-O-β-*D-glucopyranoside (**3**): colorless oil; ${\left[\alpha \right]}_{D}^{20}$ = +28.0° (c 0.05, MeOH); UV (MeOH) λ_max_ 240 nm; ^1^H and ^13^C NMR data (CD_3_OD, 500 and 125 MHz), see Table 2 and Table 3; HR-ESI-MS *m*/*z* 389.2170 [M + H]^+^ (calcd for C_19_H_33_O_8_, 389.2160). Supplementary data were available at Figures S11–S19, S56, Tables S1–S8.

(2*R*,6*S*,9*S*)-9-hydroxy-megastigman-4,7-dien-3-one-2*-O-β-*D-glucopyranoside (**4**): ${\left[\alpha \right]}_{D}^{20}$ = −38.1° (c 0.05, MeOH); UV (MeOH) λ_max_ 205 nm; ^1^H and ^13^C NMR data (CD_3_OD, 500 and 125 MHz), see Table 2 and Table 3; HR-ESI-MS *m*/*z* 387.2013 [M + H]^+^ (calcd for C_19_H_31_O_8_, 387.2017). Supplementary data were available at Figures S20–S28, S57–58, Tables S9–S32.

(+)-isololiolide *β-*D-glucopyranoside (**5**): ${\left[\alpha \right]}_{D}^{20}$ = +4.0° (c 0.02, MeOH); UV (MeOH) λ_max_ 210 nm; ^1^H and ^13^C NMR data (CD_3_OD, 500 and 125 MHz), see Table 2 and Table 3; HR-ESI-MS *m*/*z* 381.1519 [M + Na]^+^ (calcd for C_17_H_26_O_8_Na, 381.1522). Supplementary data were available at Figures S29–S37, Tables S33–S34.

(2*S*,8*R*)-loliolide *β-*D-glucopyranoside (**6a**): ${\left[\alpha \right]}_{D}^{20}$ = −51.4° (c 0.05, MeOH); UV (MeOH) λ_max_ 210 nm; ^1^H and ^13^C NMR data (CD_3_OD, 500 and 125 MHz), see Table 2 and Table 3; HR-ESI-MS *m*/*z* 381.1518 [M + Na]^+^ (calcd for C_17_H_26_O_8_Na, 381.1522). Supplementary data were available at Figures S38–S46, Tables S35–S36.

(2*R*,8*S*)-loliolide *β-*D-glucopyranoside (**6b**): ${\left[\alpha \right]}_{D}^{20}$ = +5.7° (c 0.03, MeOH); UV (MeOH) λ_max_ 210 nm; ^1^H and ^13^C NMR data (CD_3_OD, 500 and 125 MHz), see Table 2 and Table 3; HR-ESI-MS *m*/*z* 381.1518 [M + H]^+^ (calcd for C_17_H_26_O_8_Na, 381.1522). Supplementary data were available at Figures S47–S55, Tables S35–S36.

### 3.4. Acid Hydrolysis of New Compounds

The new compounds **3**, **4**, **5**, **6a,** and **6b** (1 mg each) were dissolved in 2 mL of CF_3_COOH (4 mol/L) solution and reacted at 95 °C for 3 h. The reaction was cooled to room temperature, the reaction solution was extracted with an equal volume (2 mL) of CH_2_Cl_2_ three times, and the aqueous layer after extraction was completely concentrated. A 1 mg amount of D-glucose (China Academy of Food and Drug Administration, Lot No. 110833) and the extracted aqueous phase were taken into four 25 mL round-bottomed flasks, and anhydrous pyridine (0.5 mL) and *L*-cysteine methyl ester hydrochloride (1.0 mg) were added, respectively; the reaction was carried out at 60 °C for 1 h. After cooling, 5 μL of *o*-toluene isothiocyanate was added to each reaction, and the reaction was carried out at 60 °C for 1 h. The solution was cooled to room temperature, the reaction solutions of the above four groups were extracted with an equal volume (2 mL) of CH_2_Cl_2_ three times, and the reaction solution was completely concentrated. The reaction solution of the above four groups was diluted 1-fold with pyridine, the samples (5 μL) were analyzed by HPLC (MeCN/H_2_O 25:75, 0.8 mL/min) with a detection wavelength of 250 nm, and the analytical column was a Waters chromatographic column (4.6 × 250 mm, 5 μm particle size). The acid hydrolysis products of compounds **3**, **4**, **5**, **6a,** and **6b** showed peaks at *t_R_* 20.2 min; D-glucose showed peaks at *t_R_* 20.2 min (Supporting Information Figures S59–S64).

### 3.5. Quantum Chemical Calculations (Computational NMR and ECD)

The theoretical calculations were carried out using Gaussian 09. At first, all conformers were optimized at PM6. Room-temperature equilibrium populations were calculated according to the Boltzmann distribution law, based on which dominative conformers of the population over 1% were kept. The chosen conformers were further optimized at B3LYP/6-31G (d, p) in gas phase. Vibrational frequency analysis confirmed the stable structures. NMR calculations were carried out using the Gauge-Including Atomic Orbitals (GIAO) method at mPW1PW91/6-311 + G (2d, p) level in methanol simulated by the IEFPCM model. The TMS-corrected NMR chemical shift values were averaged according to the Boltzmann distribution and fitted to the experimental values by linear regression. The calculated ^13^C- and ^1^H-NMR chemical shift values of TMS in methanol were 49.2 and 4.9 ppm respectively. To confirm the conclusions of NMR calculations, DP4+ analysis was also performed.

$$\frac{N_{i}}{N}=\frac{g_{i}e^{-\frac{E_{i}}{k_{B}T}}}{\sum g_{i}e^{-\frac{E_{i}}{k_{B}T}}}$$

where *N* is the number of conformer *i* with energy and degeneracy at temperature *T*, and *k_B_* is Boltzmann constant.

ECD calculations were conducted at a B3LYP/6-311G (d, p) level in methanol with the IEFPCM model using time-dependent density functional theory (TDDFT). The rotatory strengths for 30 excited states were calculated. The ECD spectrum was simulated using the ECD/UV analysis tool on the Yinfo Cloud Platform (https://cloud.yinfotek.com/ (27 April 2025)) by overlapping Gaussian functions for each transition, where *σ* represents the width of the band at a height of 1/*e*, while Δ*E_i_* and *R_i_* are the excitation energies and rotatory strengths for transition *i*, respectively. The spectra of the enantiomers were produced directly through mirror inversion about the horizontal axis.

$$\Delta \varepsilon \left(E\right)=\frac{1}{2.241\times 10^{-39}}\times \frac{1}{\sqrt{2\pi \sigma }}\sum_{i}^{A}\Delta E_{i}R_{i}{\mathrm{e}}^{-{\left(\frac{E-E_{i}}{2\sigma }\right)}^{2}}$$

### 3.6. Cell Culture and Hypoxia/Reoxygenation

Primary cultured neonatal rat cardiomyocytes were cultured at 37 °C with 95% air and 5% CO_2_ in DMEM supplemented with 10% FBS, 100 μg/mL streptomycin, and 100 units/mL penicillin. When the cells had grown to 70–80% confluence, they were pretreated with compounds or vehicle (DMSO) for 4 h (a time point with better efficacy in cardiomyocyte protection). The cells were then exposed to an anaerobic medium (serum- and glucose-free) in an hypoxia incubator chamber (SANYO, IX51) with an anoxic mixture gas (95% N_2_ and 5% CO_2_) for 6 h at 37 °C followed by reoxygenation for 4 h with fresh culture medium (95% air and 5% CO_2_) to simulate H/R injury in isolated hearts.

## 4. Conclusions

The phytochemical investigation of ethanolic extracts of *A. italica* led to the isolation and characterization of 31 compounds, including a new ursane triterpene (**1**) and five new norisoprenoids (**3**, **4**, **5**, **6a**, and **6b**). The chemical structures of the new compounds (including absolute configurations) were fully confirmed through comprehensive analyses involving 1D and 2D NMR, HR-ESI-MS, acid hydrolysis, and computational methods for ECD calculation. The protective effects of compounds **1–20** against hypoxia/reoxygenation (H/R)-induced cardiomyocyte injury were tested. The chemical taxonomic significance of the triterpenes (**1**, **2**, and **7–13**) may expand the use of terpenoids as a chemotaxonomy marker for the classification of the genus *Anchusa*.

## Figures and Tables

**Table 1:** ^1^H and ^13^C-NMR data (500 and 125 MHz) of compounds **1** (CD_3_OD, *δ* in ppm, *J* in Hz).

Position	*δ*_C_	*δ*_H_	Position	*δ*_C_	*δ*_H_
1	47.5	0.96, 1H, m 2.06, 1H, m	16	29.9	1.23, 1H, m 1.81, 1H, m
2	69.2	4.06, 1H, ddd, 5, 7, 10	17	45.8	
3	82.6	3.11, 1H, d, 9.5	18	57.0	2.40, 1H, s
4	55.3		19	74.3	
5	58.3	1.15, 1H, m	20	45.3	1.79, 1H, m
6	20.0	1.46, 1H, m 1.81, 1H, m	21	83.9	4.34, 1H, d, 5.5
7	34.0	1.61, 2H, m	22	34.0	1.61, 1H, m 3.34, 1H, d, 9.0
8	41.9		23	208.5	9.90, 1H, s
9	47.1	1.70, 1H, m	24	21.4	1.26, 3H, s
10	39.3		25	18.0	0.90, 3H, s
11	25.4	1.99, 1H, m 2.08, 1H, m	26	17.4	0.95, 3H, s
12	131.3	5.53, 1H, t, 4.0	27	26.2	1.39, 3H, s
13	137.7		28	185.4	
14	42.7		29	13.8	1.05, 3H, d, 7.0
15	29.3	1.08, 1H, m 1.89, 1H, m	30	28.4	1.21, 3H, s

**Table 2:** ^1^H-NMR data (500 MHz) of compounds **3–6** (CD_3_OD, *δ* in ppm, *J* in Hz) ^1^.

Position	3	4	5	6a	6b
1			1.45, 1H, t, 12.0 2.65, 1H, dd, 2.0, 12.0	1.65, 1H, dd, 4.0, 14.0 2.65, 1H, dt, 2.5, 14.5	1.77, 1H, dd, 4.0, 14.0 2.67, 1H, dt, 2.5, 14.5
2	4.20, 1H, s	4.17, 1H, s	4.27, 1H, tt, 4.0, 12.0	4.28, 1H, m	4.30, 1H, m
3			1.38, 1H, t, 12.0 2.18, 1H, dd, 2.0, 12.0	1.55, 1H, dd, 4.0, 14.0 2.20, 1H, dt, 2.5, 14.5	1.44, 1H, dd, 4.0, 14.0 2.21, 1H, dt, 2.5, 14.5
4	5.81, 1H, d, 1.0	5.91, 1H, d, 1.0			
5					
6	2.10, 1H, m	2.77, 1H, d, 7.5	5.77, 1H, s	5.74, 1H, s	5.74, 1H, s
7	1.60, 1H, m 2.11, 1H, m	5.76, 1H, m			
8	1.62, 2H, m	5.77, 1H, m			
9	3.87, 1H, m	4.40, 1H, m	1.31, 3H, s	1.27, 3H, s	1.28, 3H, s
10	2.03, 3H, d, 1.0	1.92, 3H, d, 10.0	1.28, 3H, s	1.43, 3H, s	1.45, 3H, s
11	0.88, 3H, s	0.90, 3H, s	1.59, 3H, s	1.75, 3H, s	1.74, 3H, s
12	1.19, 3H, s	1.11, 3H, s			
13	1.19, 3H, d, 6.0	1.30, 3H, d, 6.5			
Glc					
1	4.33, 1H, d, 8.0	4.36, 1H, d, 7.5	4.42, 1H, d, 8.0	4.37, 1H, d, 8.0	4.39, 1H, d, 8.0
2	3.13, 1H, m	3.17, 1H, m	3.12, 1H, m	3.17, 1H, m	3.17, 1H, m
3	3.33, 1H, m	3.33, 1H, m	3.29, 1H, m	3.36, 1H, m	3.35, 1H, m
4	3.24, 1H, m	3.29, 1H, m	3.24, 1H, m	3.27, 1H, m	3.27, 1H, m
5	3.24 ^1^	3.22, 1H, m	3.29 ^1^	3.27, 1H, m	3.27 ^1^
6	3.65, 1H, m 3.83, 1H, m	3.67, 1H, m 3.83, 1H, m	3.63, 1H, m 3.89, 1H, m	3.64, 1H, m 3.85, 1H, m	3.65, 1H, m 3.86, 1H, m

^1^ Overlapped with other signals.

**Table 3:** ^13^C-NMR data (125 MHz) of compounds **3–6** (CD_3_OD, *δ* in ppm).

Position	3	4	5	6a	6b
1	43.2	42.8	45.6	42.9	45.4
2	77.2	77.4	72.9	74.2	74.5
3	201.2	200.9	48.9	46.9	44.4
4	124.0	125.0	35.9	37.1	37.0
5	168.6	164.7	183.8	185.8	185.7
6	54.5	58.6	113.4	113.1	113.1
7	25.9	128.3	173.8	174.4	174.4
8	38.2	138.5	88.3	88.9	88.9
9	75.4	77.0	30.1	30.9	31.0
10	24.6	23.7	25.1	26.6	26.7
11	21.0	25.5	25.4	27.1	27.0
12	24.5	21.0			
13	19.8	21.0			
Glc					
1	102.0	102.5	102.7	102.9	103.2
2	75.1	75.3	74.8	75.3	75.3
3	78.1	78.1	77.8	78.5	78.4
4	71.7	71.5	71.5	71.7	71.7
5	77.8	78.0	77.8	77.9	77.9
6	62.9	62.6	62.6	62.8	62.8

**Table 4:** A comparison of the compounds from *Anchusa italica* with other species in the genus *Anchusa* or Boraginaceae ^1,2,3^.

Number	Compound	Type	*A. italica*	
1	2*α*,3*β*,19*α*-trihydroxy-23-formyl-urs-12-en-28,21*β-*olide	triterpene	1, 2	
2	2α,3*β*,21,24-tetrahydroxyoleanan-12-en-28-oic acid	triterpene	1	
3	(2*R*,6*R*,9*S*)-9-Hydroxy-4-megastigmen-3-one-2-*O*-*β*-D-glucopyranoside	norisoprenoids	1, 2	
4	(2*R*,6*S*,9*S*)-9-Hydroxy-megastigman-4,7-dien-3-one-2-*O*-*β*-D-glucopyranoside	norisoprenoids	1, 2	
5	(+)-Isololiolide *β*-D-glucopyranoside	norisoprenoids	1, 2	
6a	(2*S*,8*R*)-Loliolide *β*-D-glucopyranoside	norisoprenoids	1, 2	
6b	(2*R*,8*S*)-Loliolide *β*-D-glucopyranoside	norisoprenoids	1, 2	
7	Niga-ichigoside F 1	triterpene	1	
8	Niga-ichigoside F 2	triterpene	1	
9	Pinfaensin	triterpene	1,	
10	Glucosyl tormentate	triterpene	1	
11	23-Hydroxytormentic acid	triterpene	1	
12	24-epi-pinfaensic acid	triterpene	1	
13	19*α*-Hydroxyasiatic acid	triterpene	1	
14	(+)-Vomifoliol	norisoprenoids	1	
15	Lippianoside E	norisoprenoids	1, 3	
16	3-oxo-α-ionol-*β*-D-glucopyranoside	norisoprenoids	1, 3	
17	Asysgangoside	norisoprenoids	1	
18	(6*S*,9*R*)-Roseoside	norisoprenoids	1, 3	*Borago officinalis* L.
19	Sammangaoside B	norisoprenoids	1, 3	
20	(+)-Isololiolide	norisoprenoids	1, 3	
21	Tricin	flavone	1, 3	
22	6-Hydroxykaempferol 3-*β*-rutinoside	flavonol	1, 3	
23	Kaempferol 3-*O*-rutinoside	flavonol	1, 3	*Anchusa strigosa* Banks & Sol.
24	Narcissin	flavonol	1, 3	*Heliotropium angiospermum*
25	(+)-Syringaresinol	lignans	1, 3	*Moltkia aurea* Boiss.
26	(+)-Mediaresinol	lignans	1	
27	Dehydrodiconiferyl alcohol	lignans	1, 3	
28	Vibruresinol	lignans	1, 3	
29	Dehydrodiconiferyl alcohol 4-*O*-*β*-D-glucopyranoside	lignans	1, 3	
30	Syringaresinol-4′-*O*-*β*-D-glucopyranoside	lignans	1, 3	*Moltkia aurea* Boiss.

^1^ Reported in this study; ^2^ New compound; ^3^ Compound reported from *Anchusa italica* for the first time.

## Data Availability

Data is contained within the article or Supplementary Materials.

## Supplementary Materials

The following supporting information can be downloaded at: https://www.mdpi.com/article/10.3390/plants14091385/s1, Figures S1–S10, HR-ESI-MS, NMR, CD, and UV data of **1**; Figures S11–S19: HR-ESI-MS, NMR, CD, and UV data of **3**; Figures S20–S28: HR-ESI-MS, NMR, CD, and UV data of **4**; Figures S29–S37: HR-ESI-MS, NMR, CD, and UV data of **5**; Figures S38–S55: HR-ESI-MS, NMR, CD, and UV data of **6**; Figures S56–58: Computational NMR results of compounds **3** and **4**; Figures S59–S64: Acid hydrolysis of compounds **3–5**, **6a**, and **6b**; Figures S65: The ability of compounds **1–20** against H/R-induced neonatal rat cardiomyocytes injury. Figure S66: Plant photograph of *Anchusa italica* Retz.; Table S1–S36: Text data of computational NMR and ECD for the new compounds.
