# Supplementary material for: Ursane Triterpenes and Norisoprenoids from Anchusa italica Retz. and Their Chemotaxonomic Significance
Source: Plants (Basel). 2025 May 3;14(9):1385. doi: 10.3390/plants14091385 (PMC12073295; doi:10.3390/plants14091385)
Supplement: Supplementary file 1 [file plants-14-01385-s001.zip › plants-3526455-supplementary.pdf]

Ursane Triterpenes and Norisoprenoids from *Anchusa italica* Retz. and Their Chemotaxonomic Significance

Linchuang Shen [1], Bingchen Han [2], Zhiliang Ma [1, 3,] Xianju Huang [1], Guangzhong Yang [1], Yanfeng Zeng [1], Maochuan Liao [1], Ruixi Gao [1,*] and Jun Li [1,4,*]

[1] School of Pharmaceutical Sciences, South-Central Minzu University, Wuhan 430074, China; 2022110463@mail.scuec.sdu.cn (L.S.)

[2] College of Life Sciences, South-Central Minzu University, Wuhan 430074, China

[3] Qinghai Tibetan Medicine Research Institute, Xining 810016, China

[4] Science and Technology Cooperation Base for Evaluation and Utilization of Traditional Medical Resources, South-Central Minzu University, Wuhan 430074, China

* Correspondence: dr.rxgao@gmail.com (R.G.); lijun-pharm@hotmail.com (J.L.)

CONTENTS

**Figures S1-S10.** HRESIMS, NMR,CD , UV data of **1**	**3-7**
**Figures S11-S19.** HRESIMS, NMR,CD , UV data of **3**	**7-12**
**Figures S20-S28.** HRESIMS, NMR,CD , UV data of **4**	**12-16**
**Figures S29-S37.** HRESIMS, NMR,CD , UV data of **5**	**17-21**
**Figures S38-S55.** HRESIMS, NMR,CD , UV data of **6**	**21-30**
NMR and ECD calculation data of **3**	**31-41**
NMR and ECD calculation data of **4**	**42-69**
ECD calculation data of **5**	**70**
ECD calculation data of **6a**	**71**
**Figures S59-S64.** Acid hydrolysis of compounds **3-5**, **6a** and **6b**	**72-74**
**Figures S65.** The ability of compounds **1-20** against H/R-induced neonatal rat cardiomyocytes injury.	**75**
**Figure S66. Photographs of *Anchusa italica* Retz.**	**76**

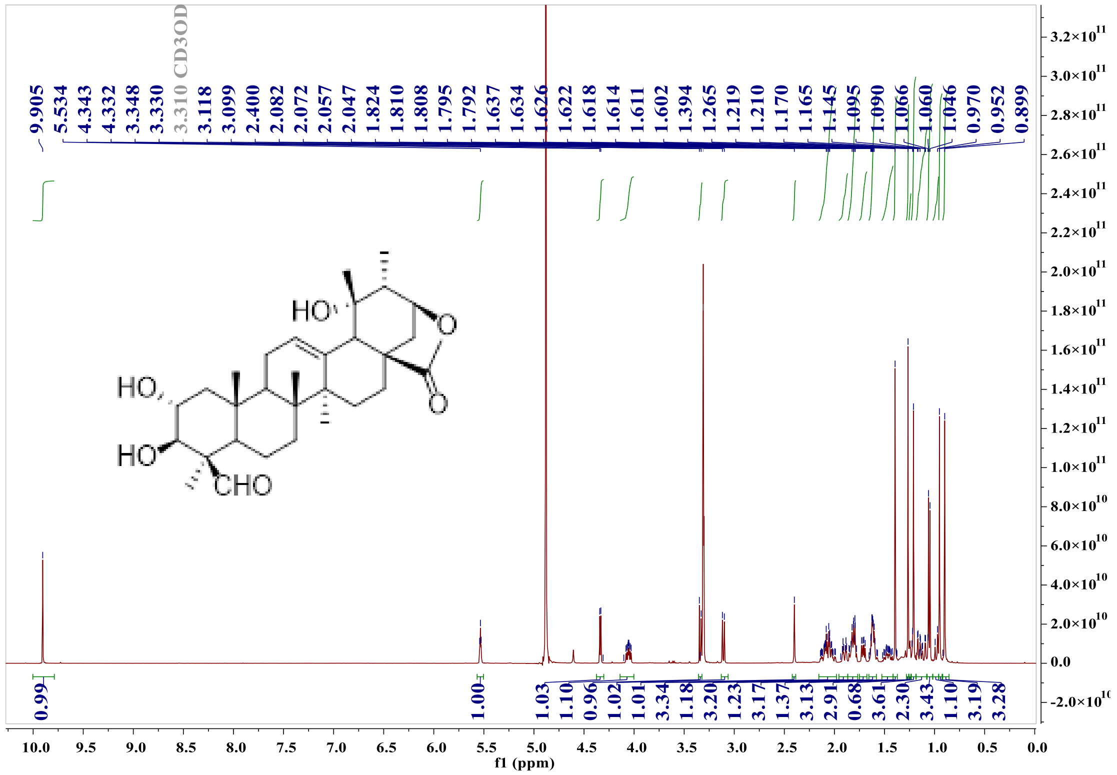

Figure S1: ^{1}H-NMR (600 MHz, CD_3OD) spectrum of compound **1**

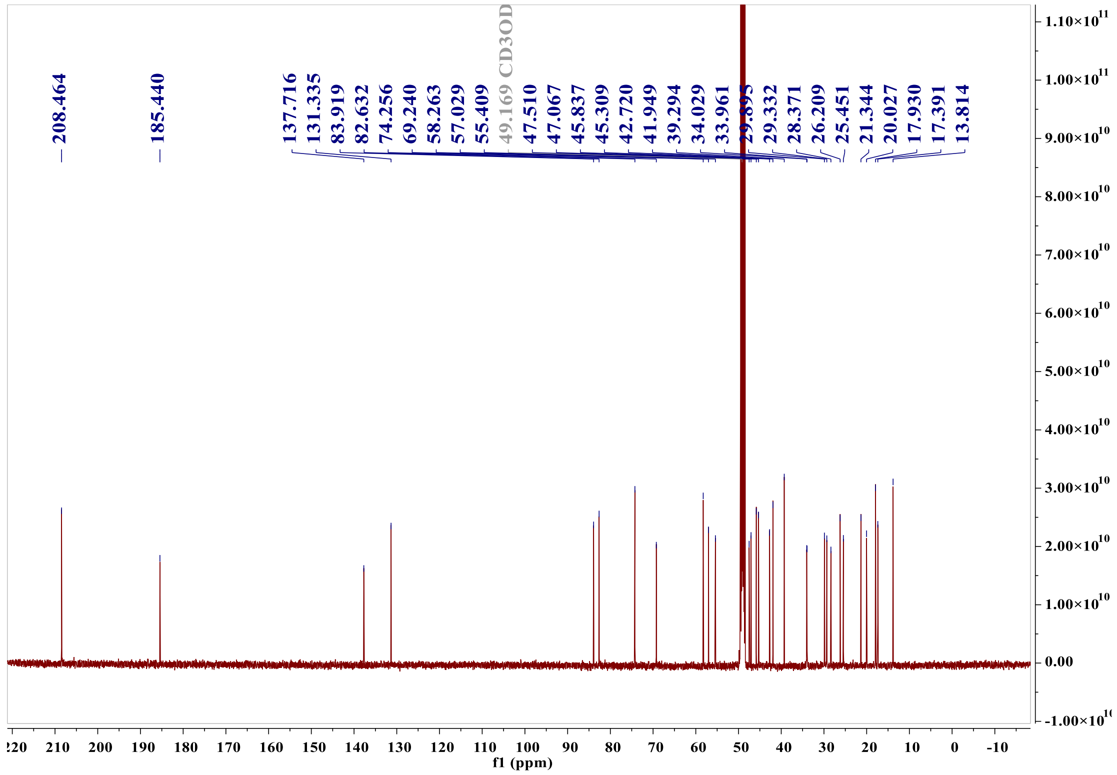

Figure S2: ^{13}C-NMR (150 MHz, CD_3OD) spectrum of compound **1**

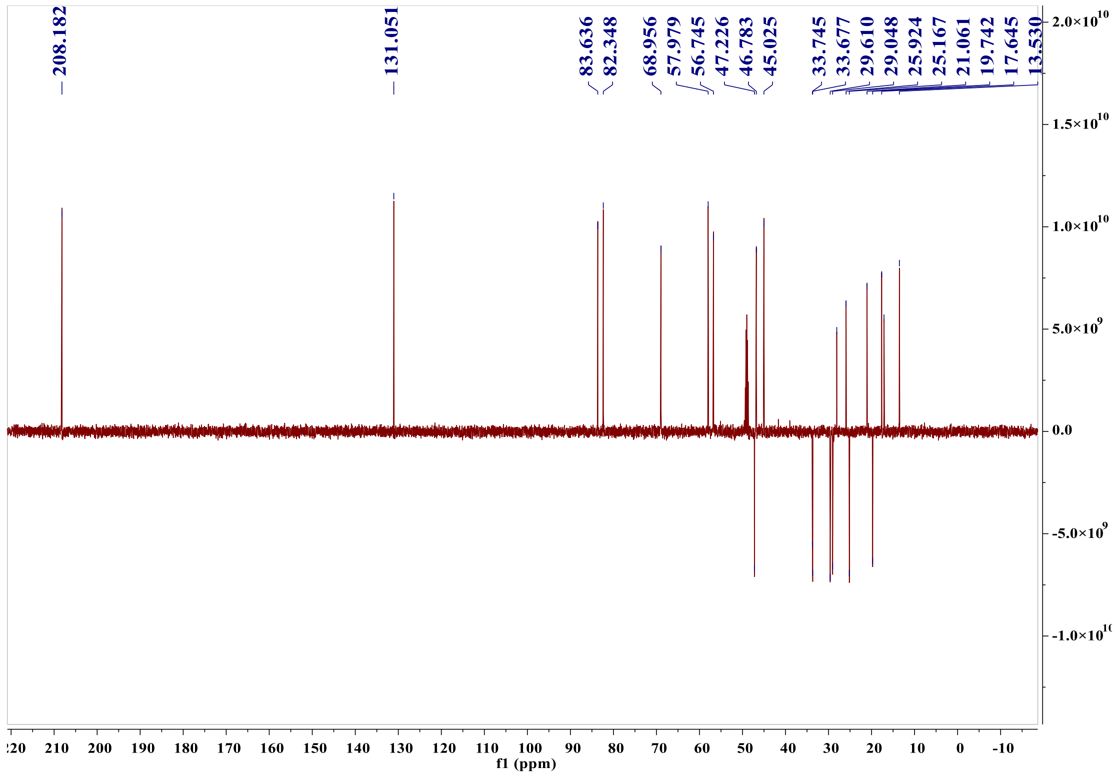

Figure S3: ^{13}C-NMR-DEPT (θ =135°) spectrum of compound **1**

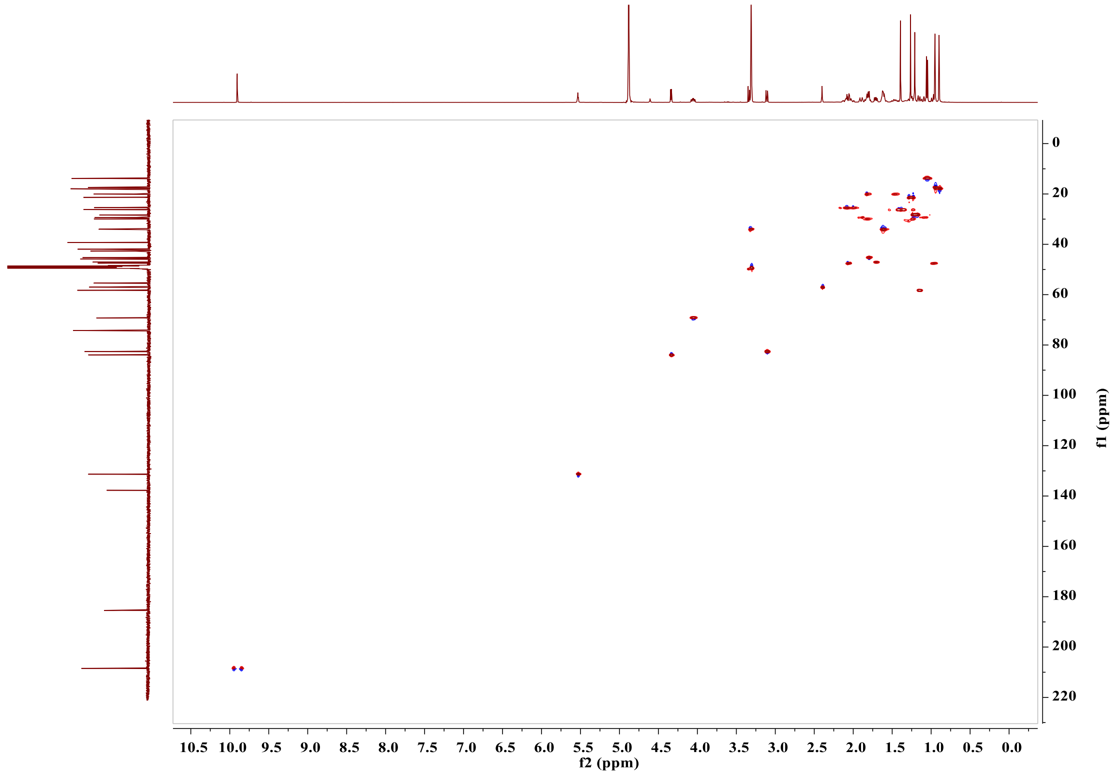

Figure S4: HSQC spectrum of compound **1**

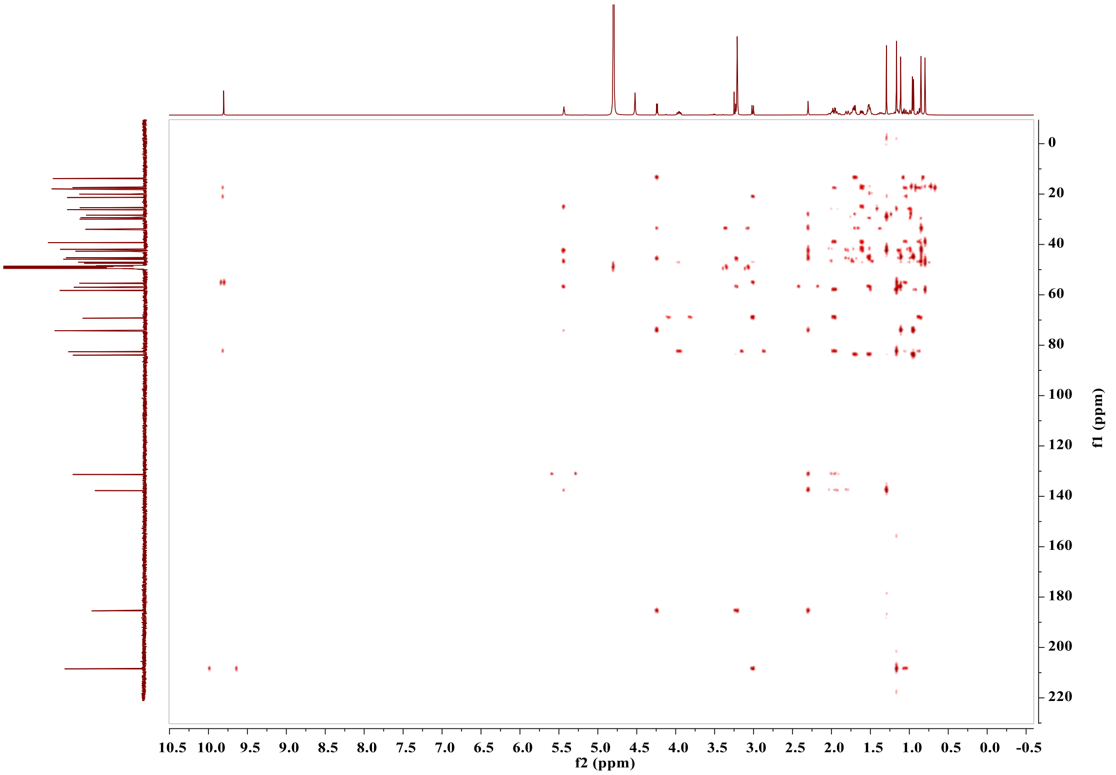

Figure S5: HMBC spectrum of compound **1**

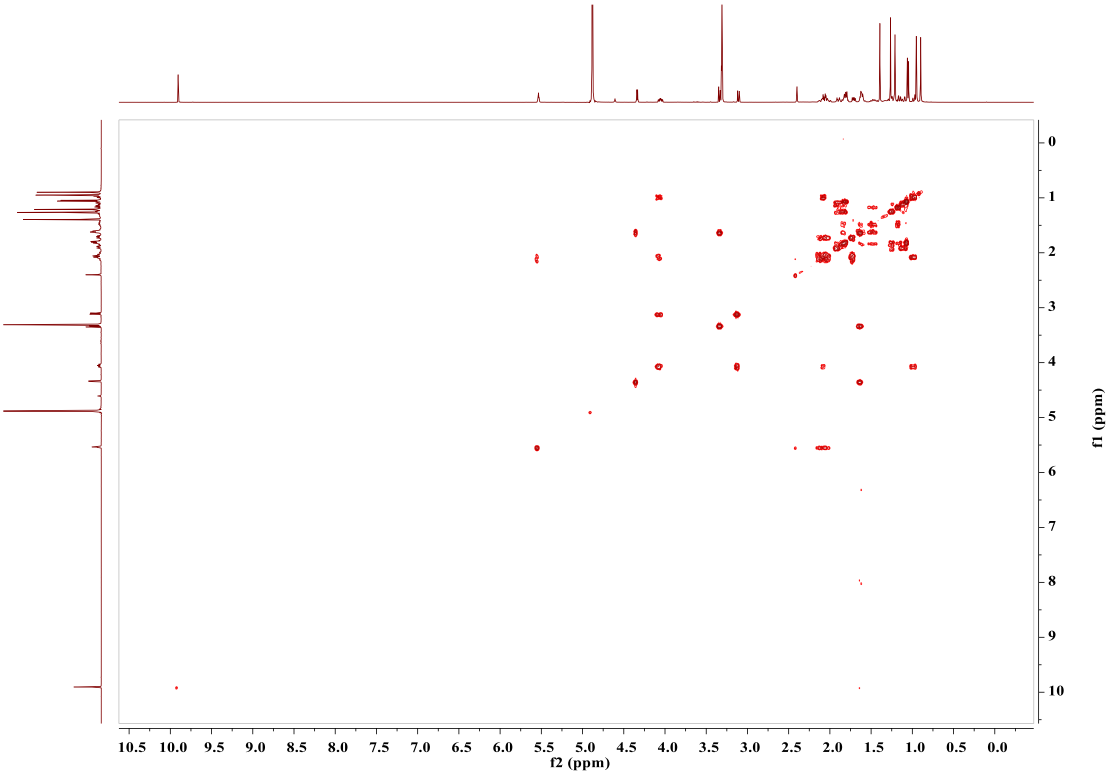

Figure S6: ^{1}H-^{1}H COSY spectrum of compound **1**

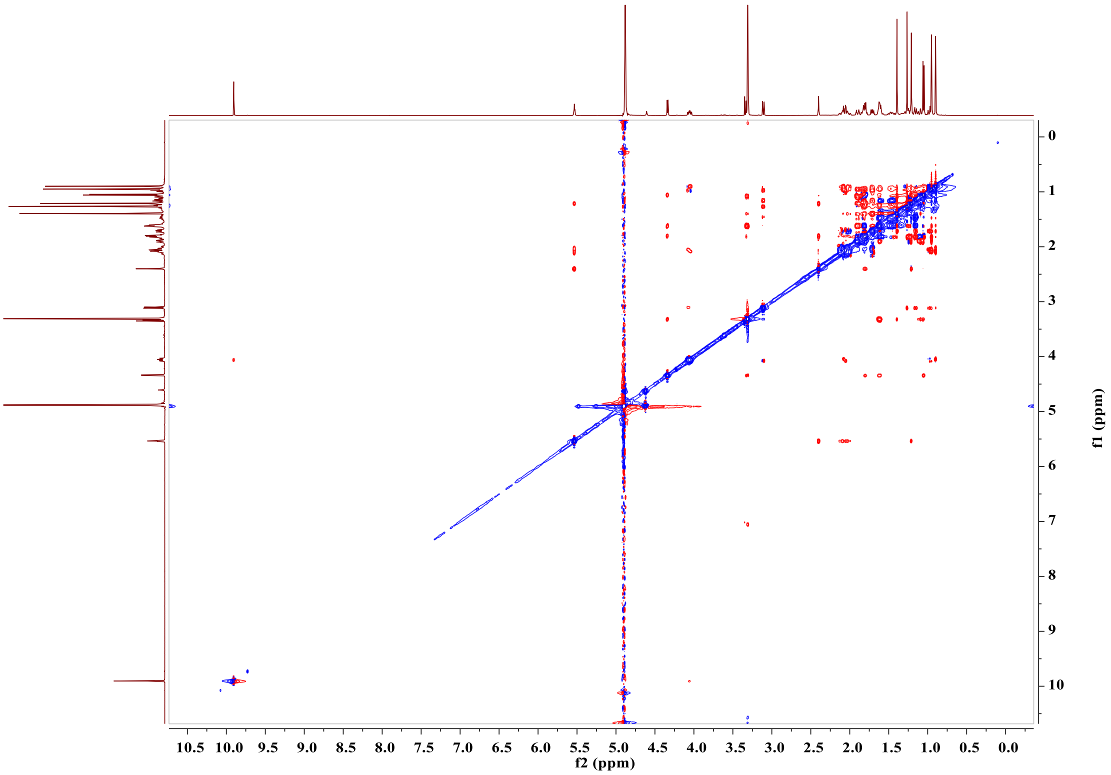

Figure S7: ROESY spectrum of compound **1**

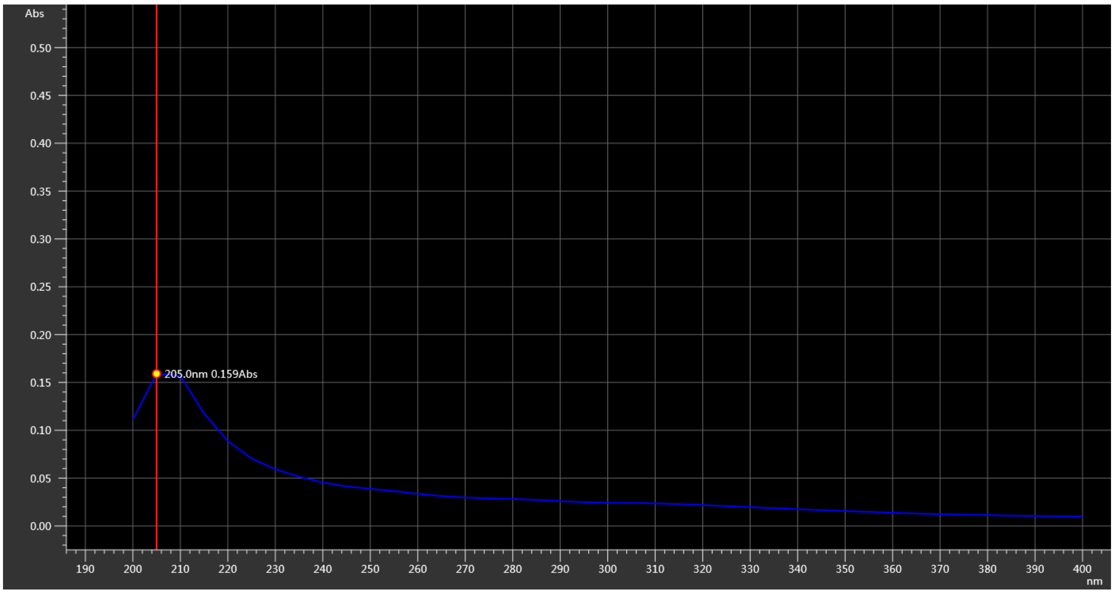

Figure S8: UV spectrum of compound **1**

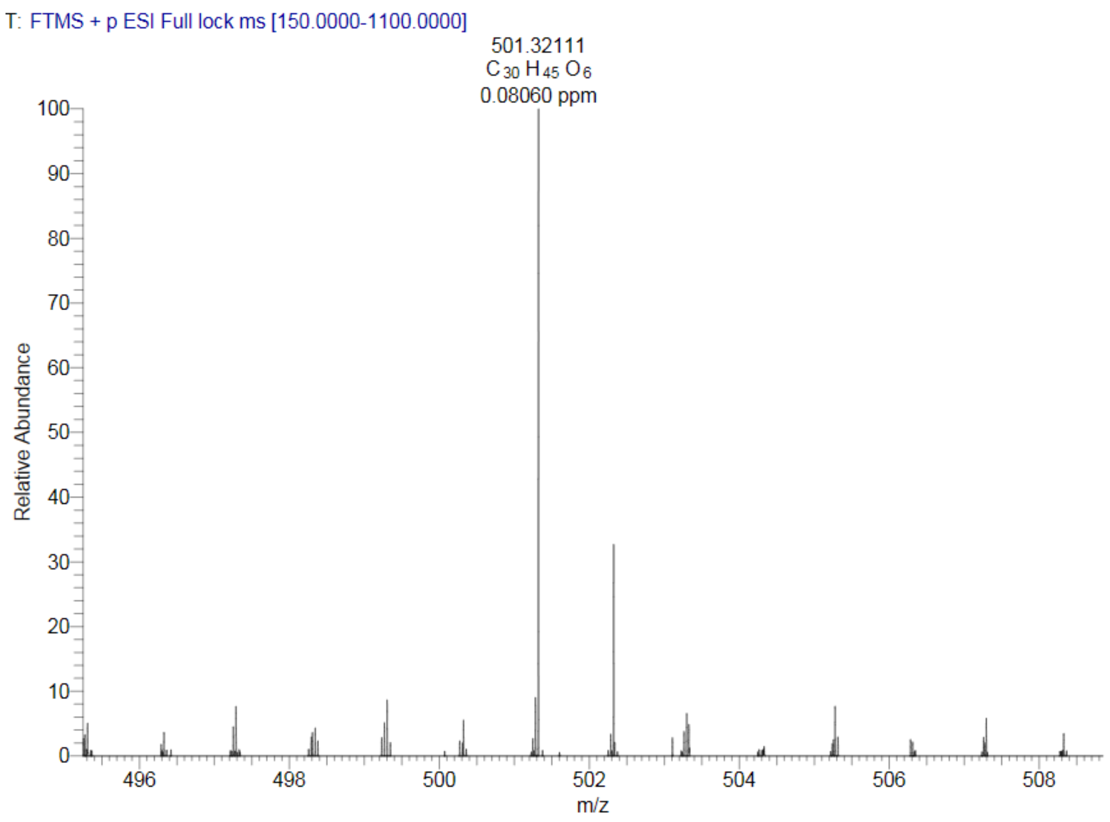

Figure S9: HR-ESI-MS of compound **1**

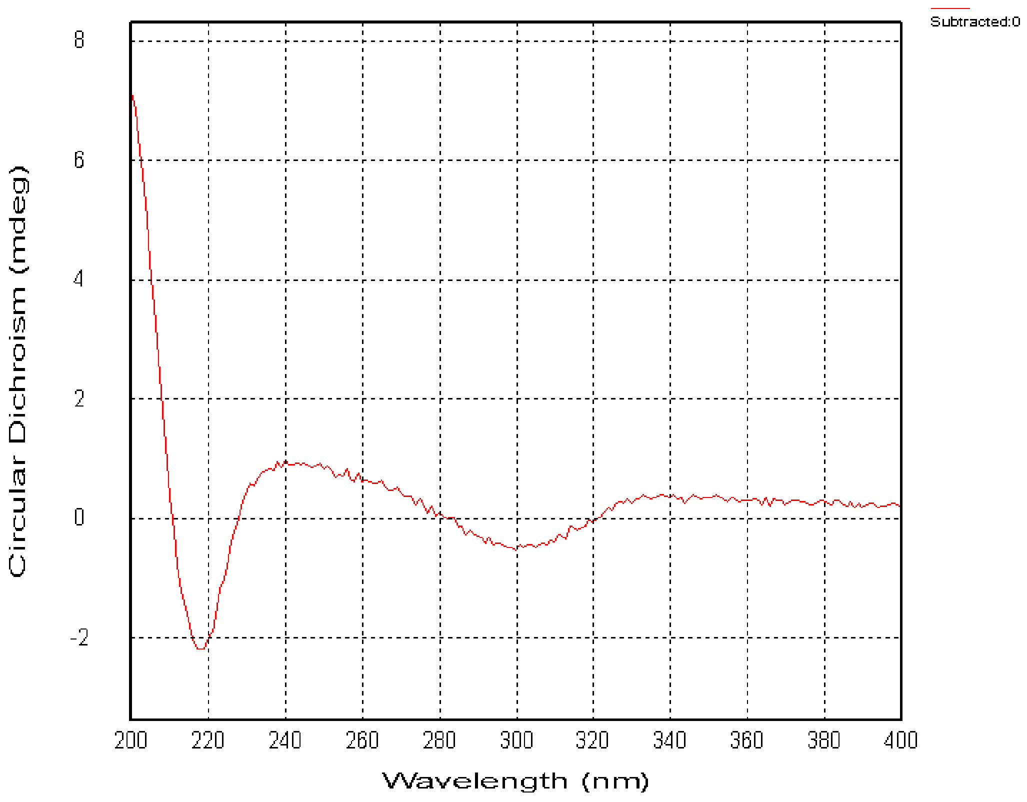

Figure S10: CD of compound **1**

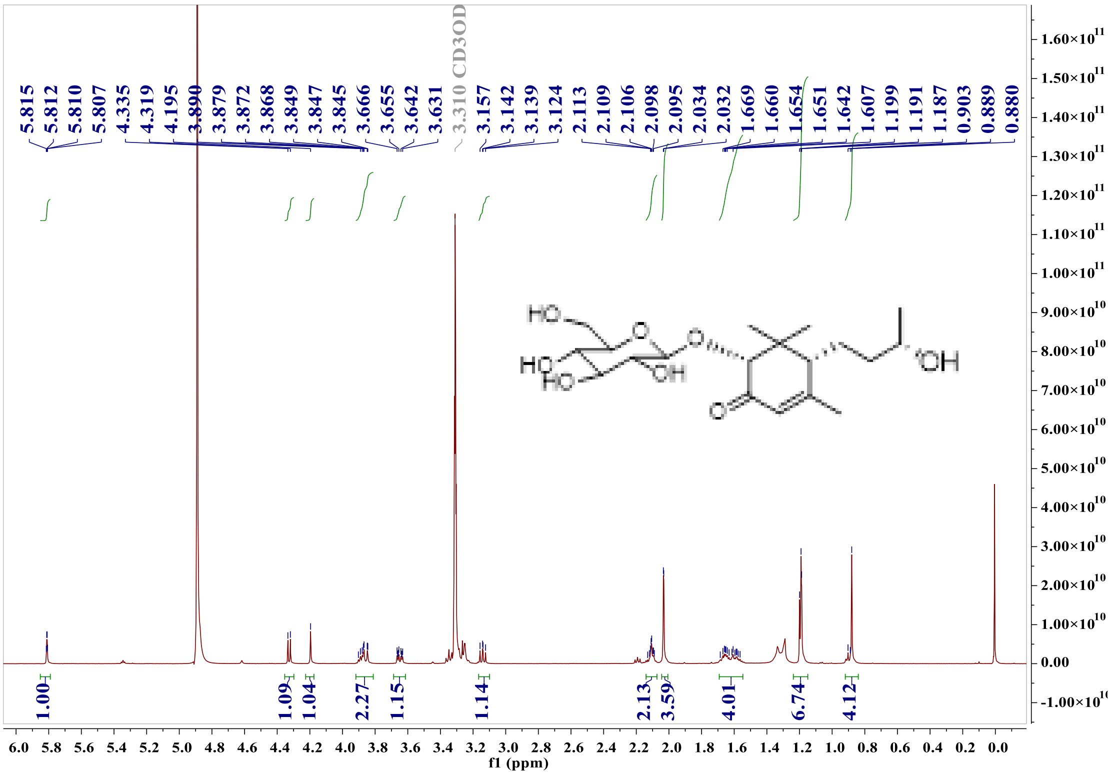

Figure S11: 1H-NMR (600 MHz, CD_3OD) spectrum of compound 3

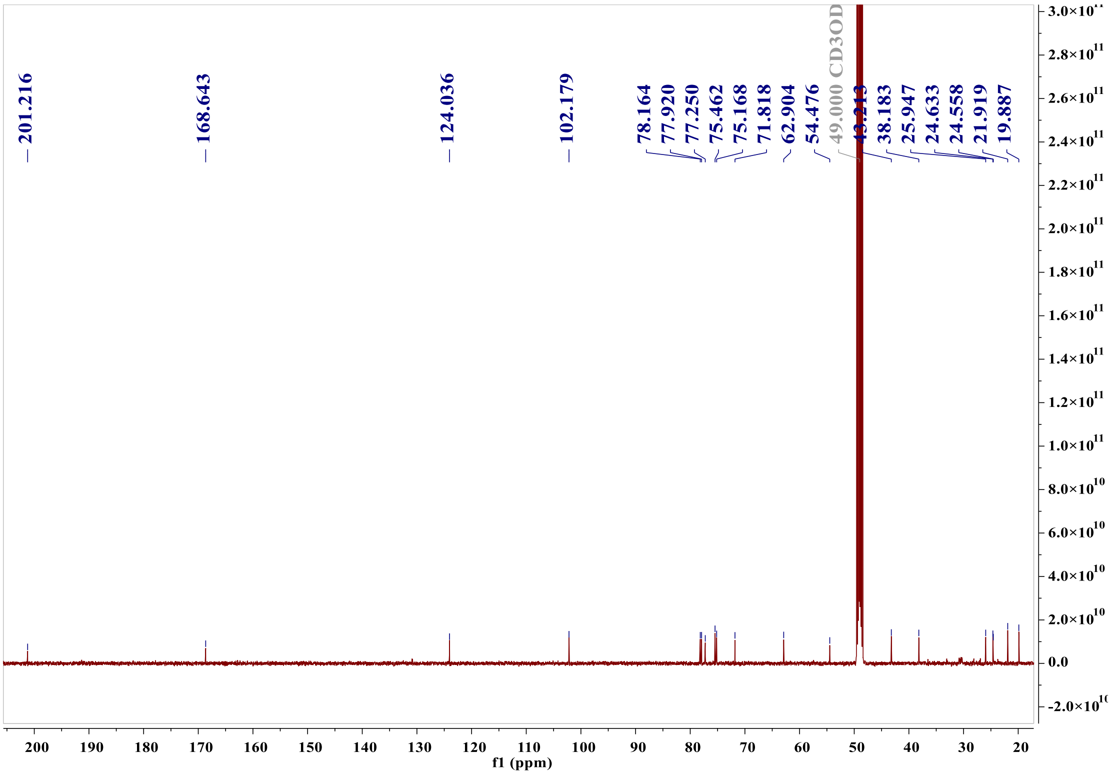

Figure S12: 13C-NMR (150 MHz, CD_3OD) spectrum of compound 3

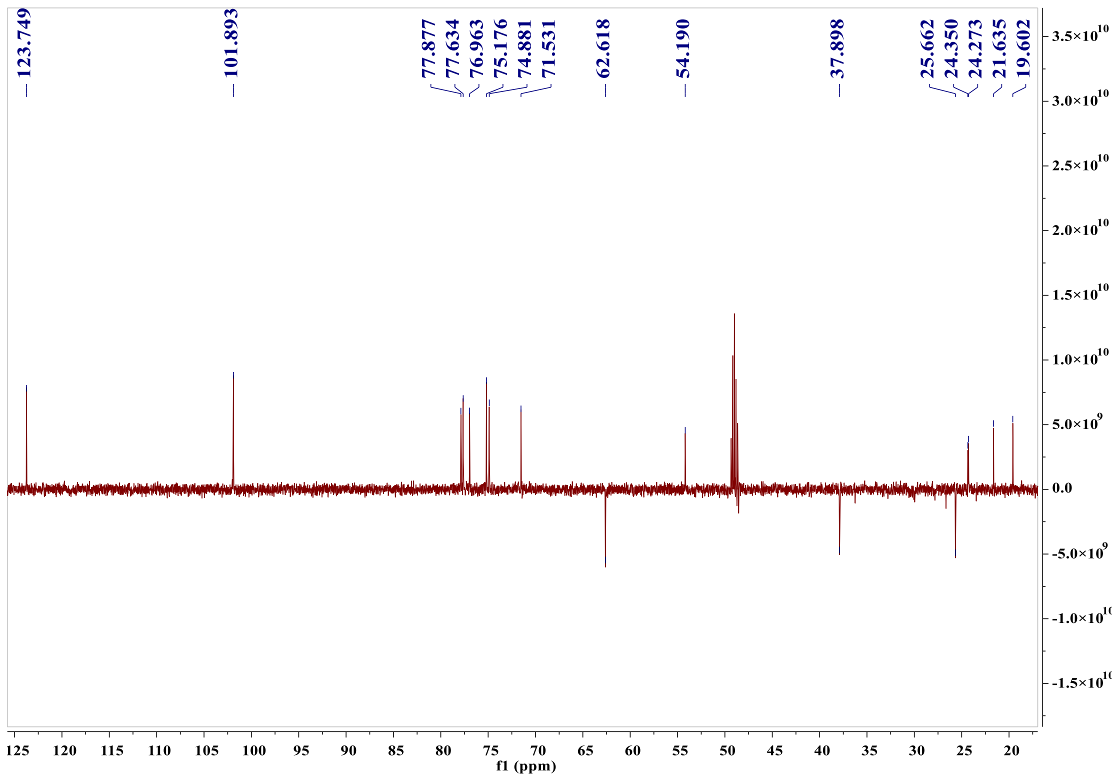

Figure S13: ^{13}C-NMR-DEPT (θ =135°) spectrum of compound **3**

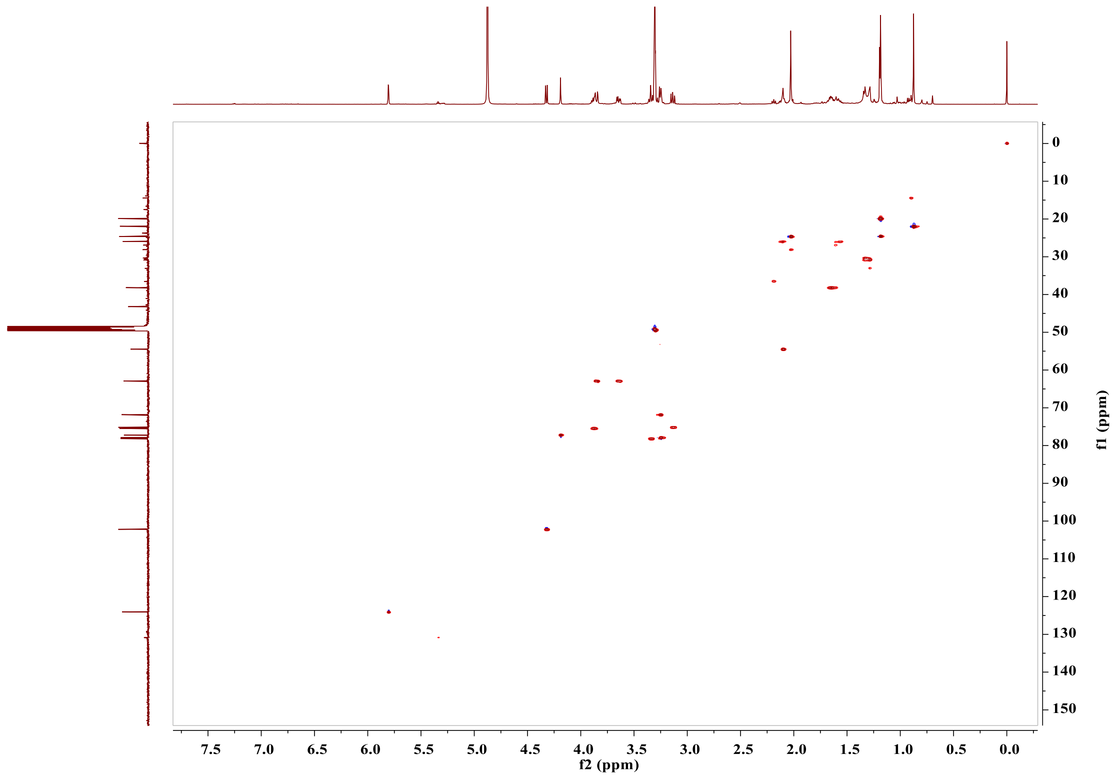

Figure S14: HSQC spectrum of compound **3**

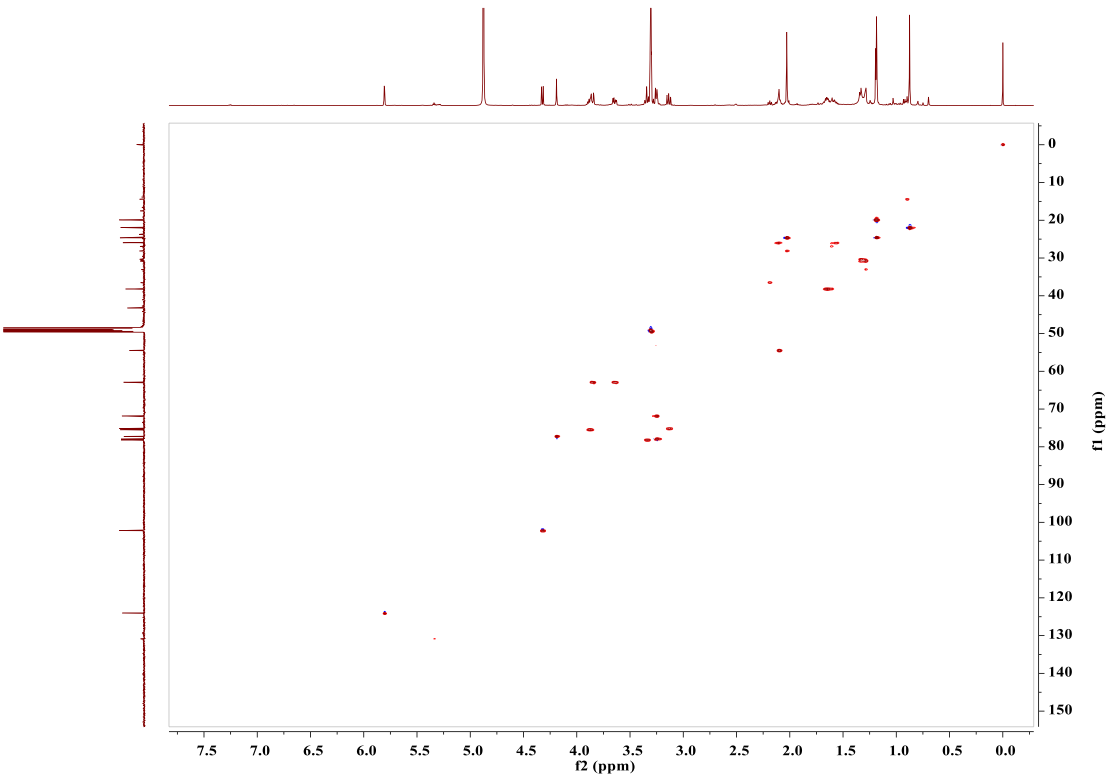

Figure S15: HMBC spectrum of compound **3**

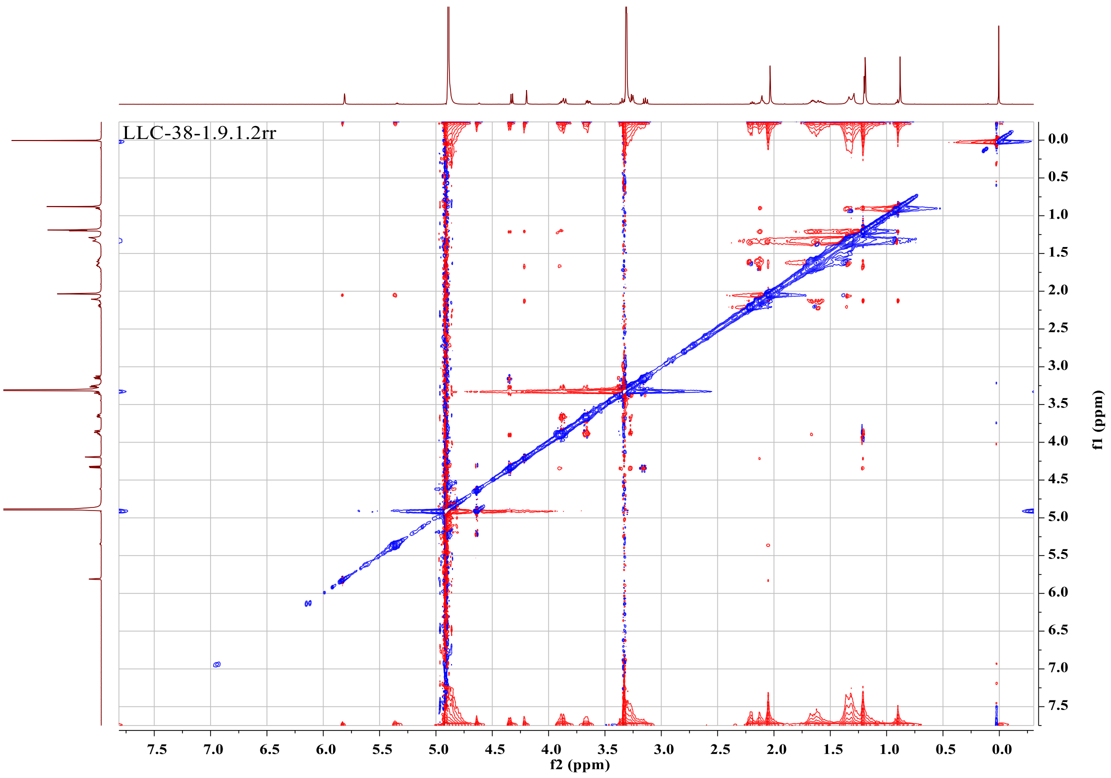

Figure S16: ROESY spectrum of compound **3**

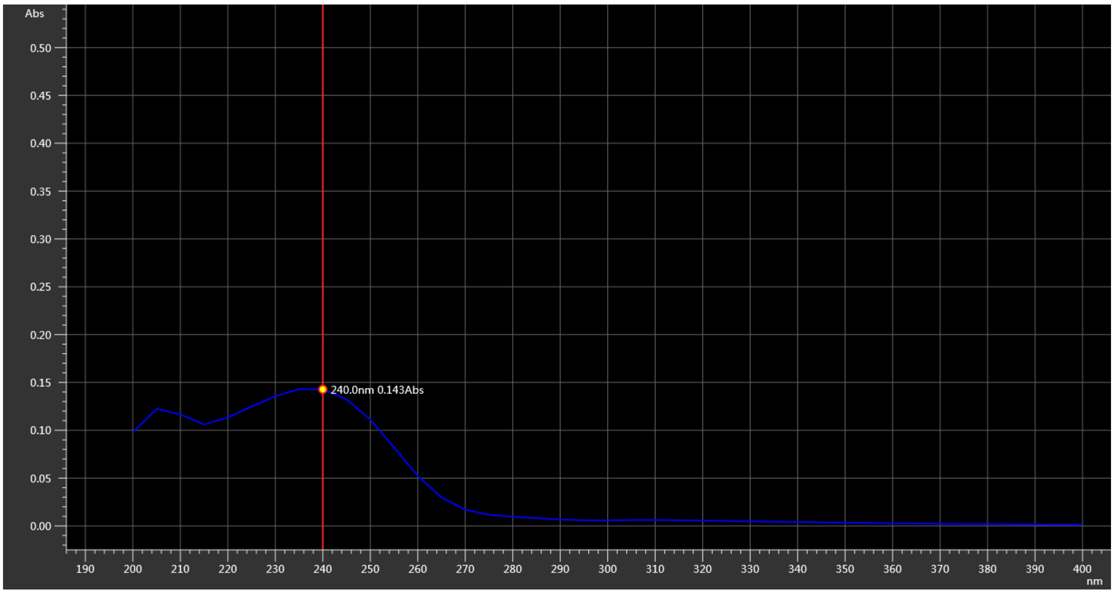

Figure S17: UV spectrum of compound **3**

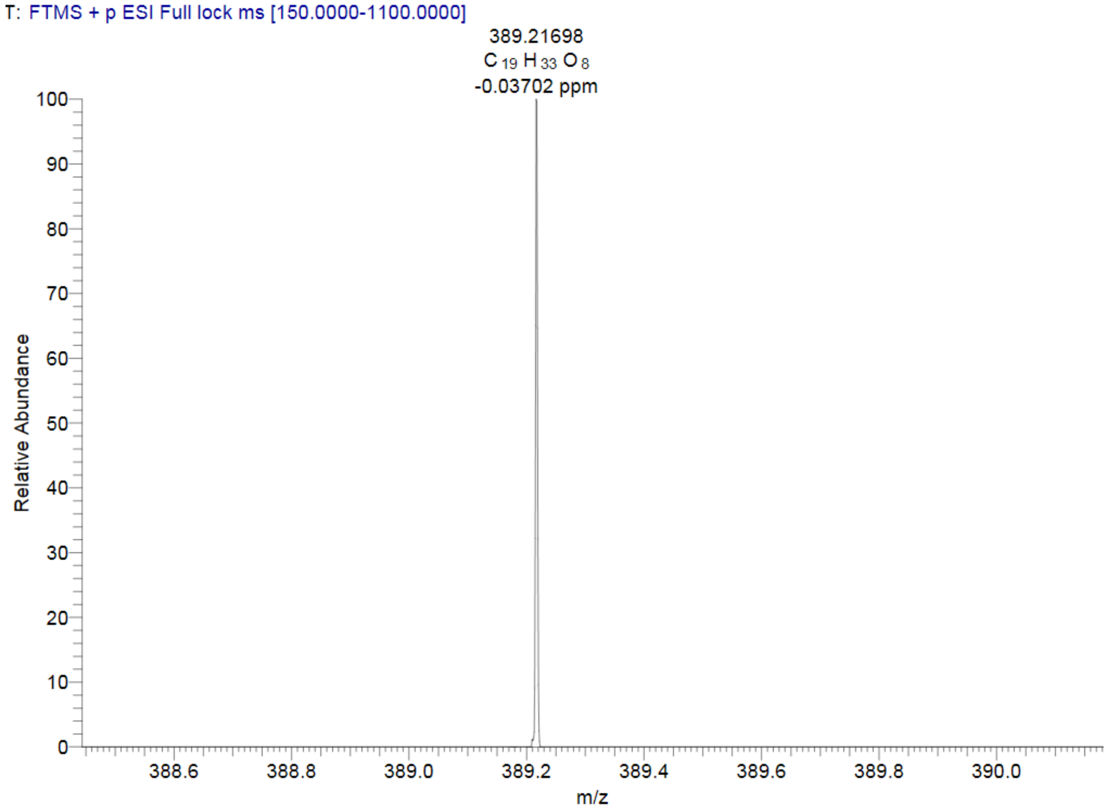

Figure S18: HR-ESI-MS of compound **3**

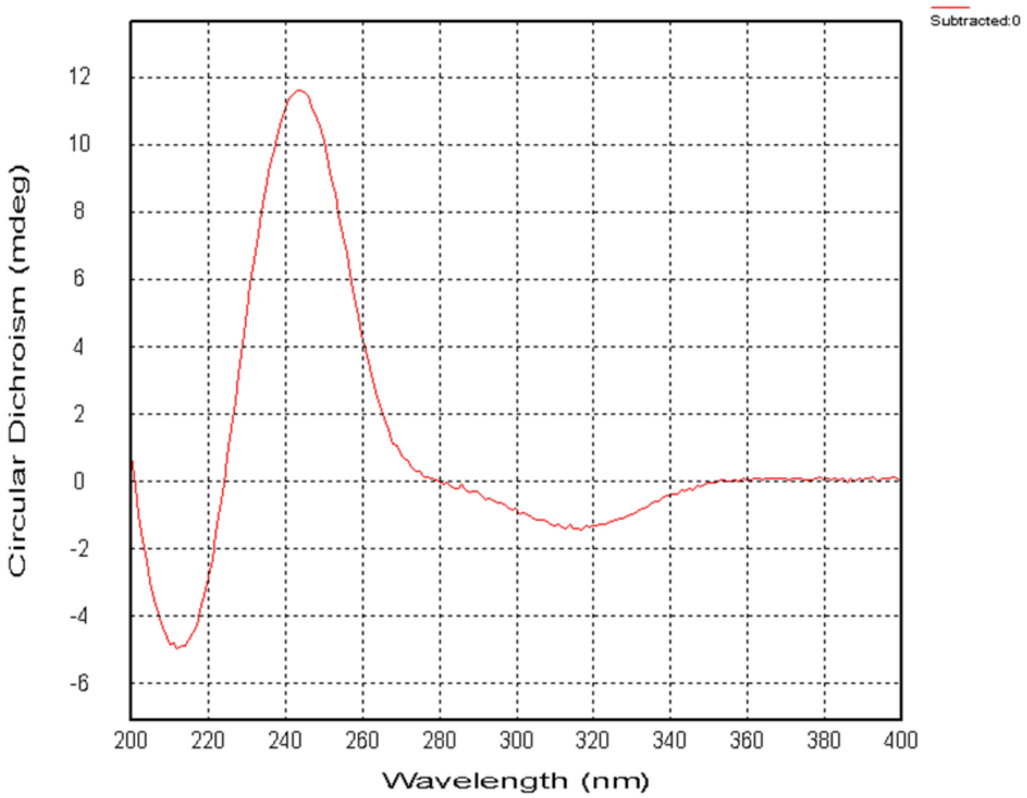

Figure S19: CD spectrum of compound **3**

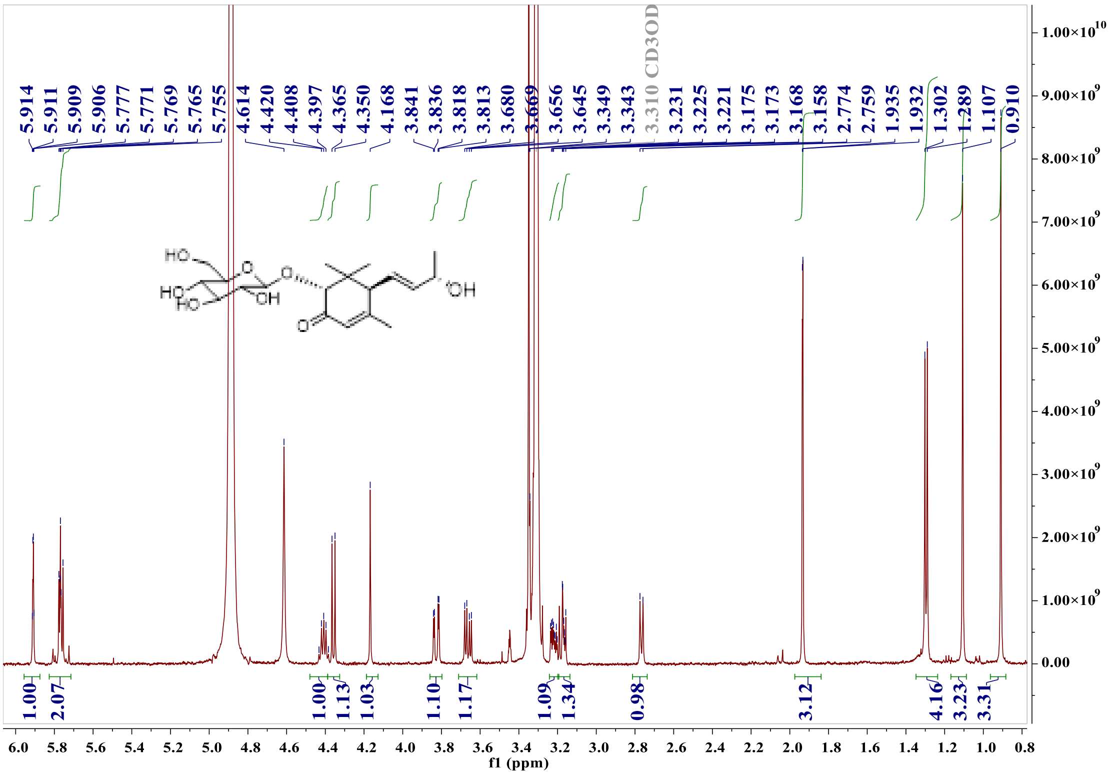

Figure S20: ^{1}H-NMR (500 MHz, CD_3OD) spectrum of compound **4**

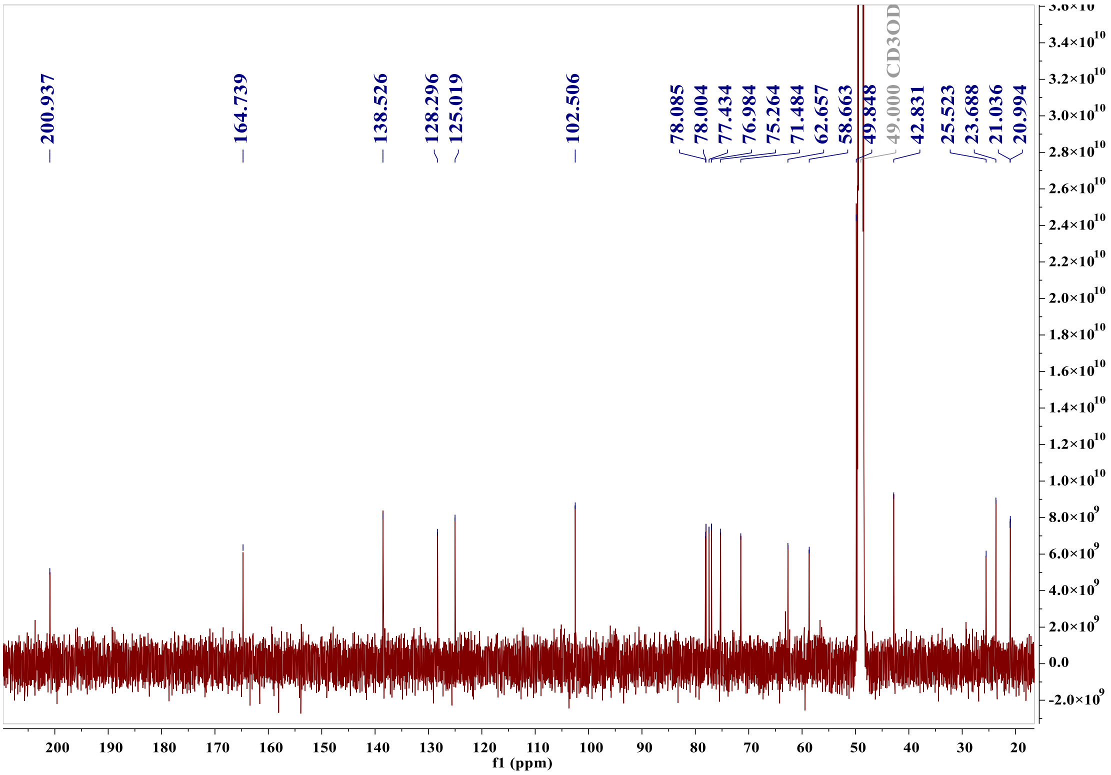

Figure S21: ^{13}C-NMR (125 MHz, CD_3OD) spectrum of compound **4**

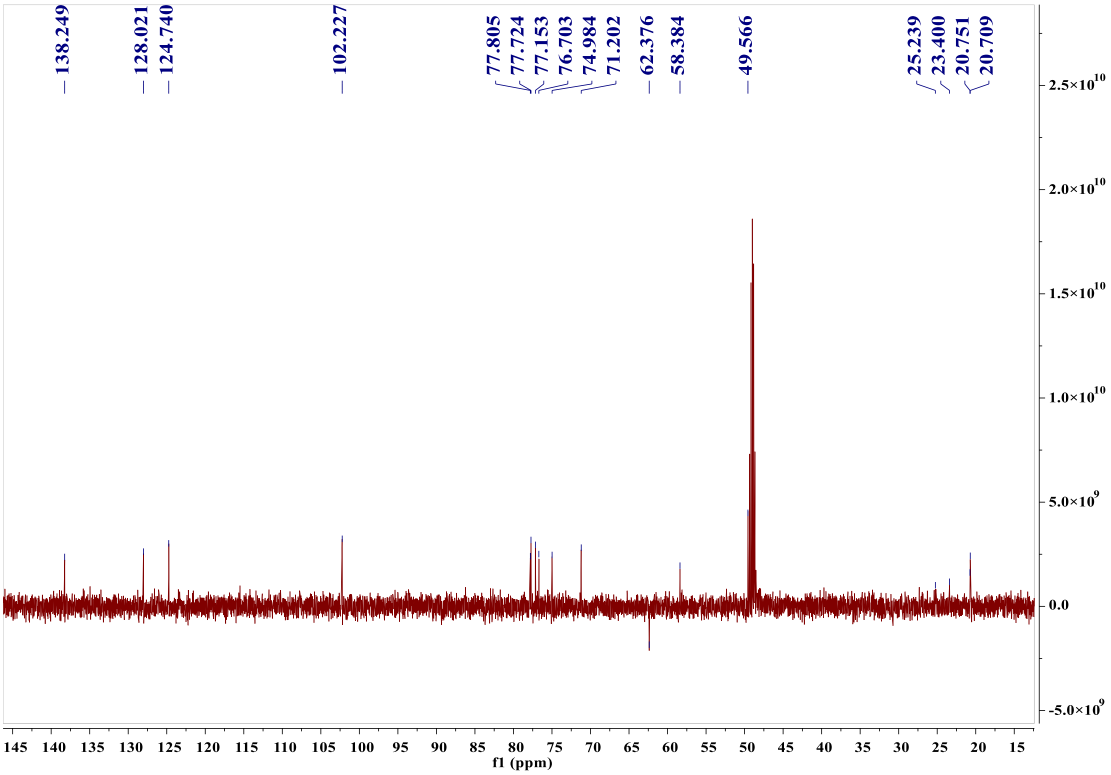

Figure S22: ^{13}C-NMR-DEPT (θ =135°) spectrum of compound **4**

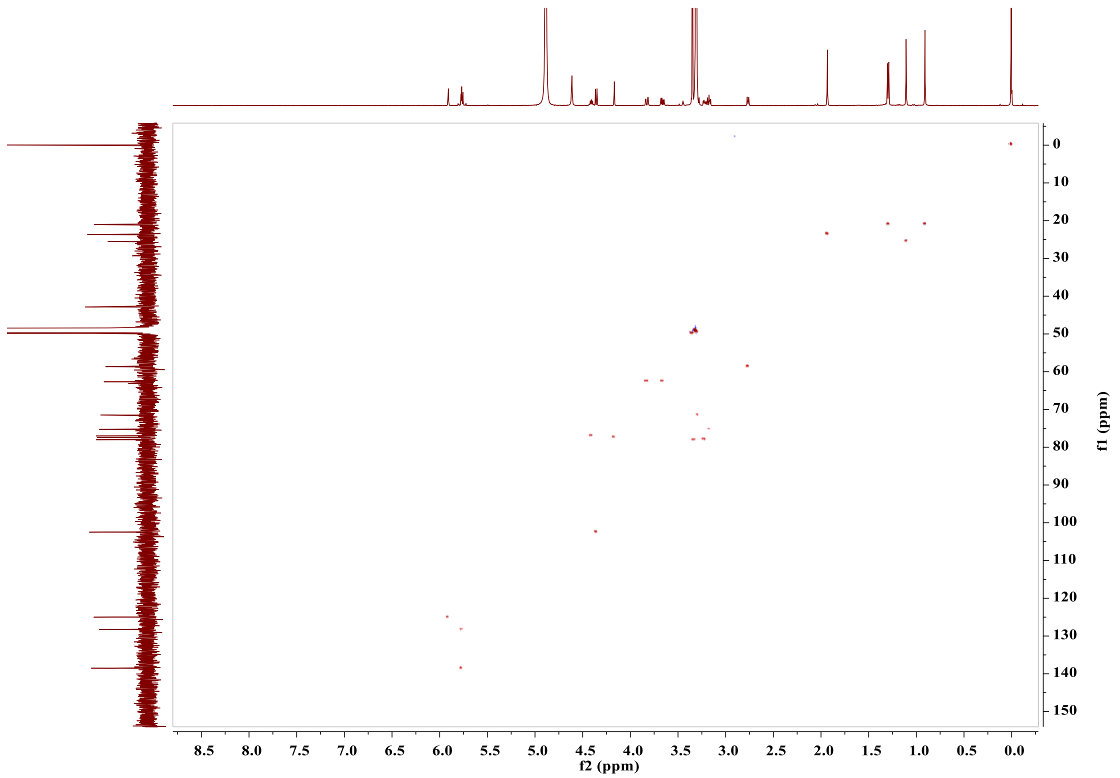

Figure S23: HSQC spectrum of compound **4**

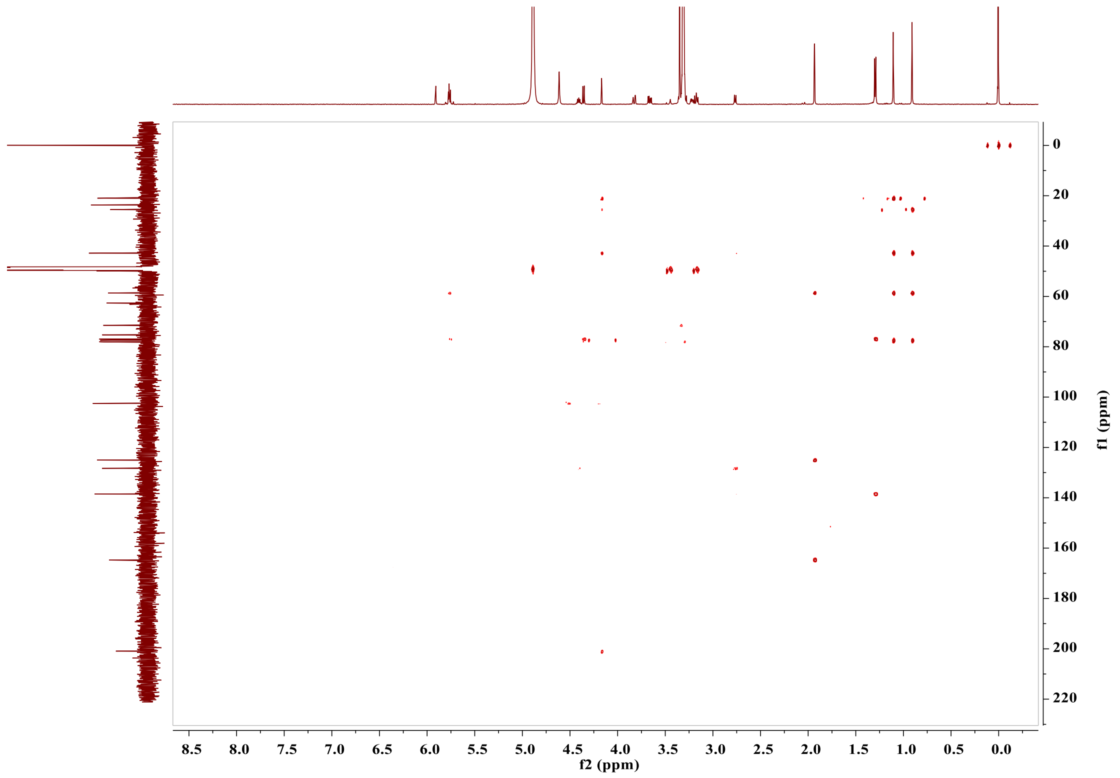

Figure S24: HMBC spectrum of compound **4**

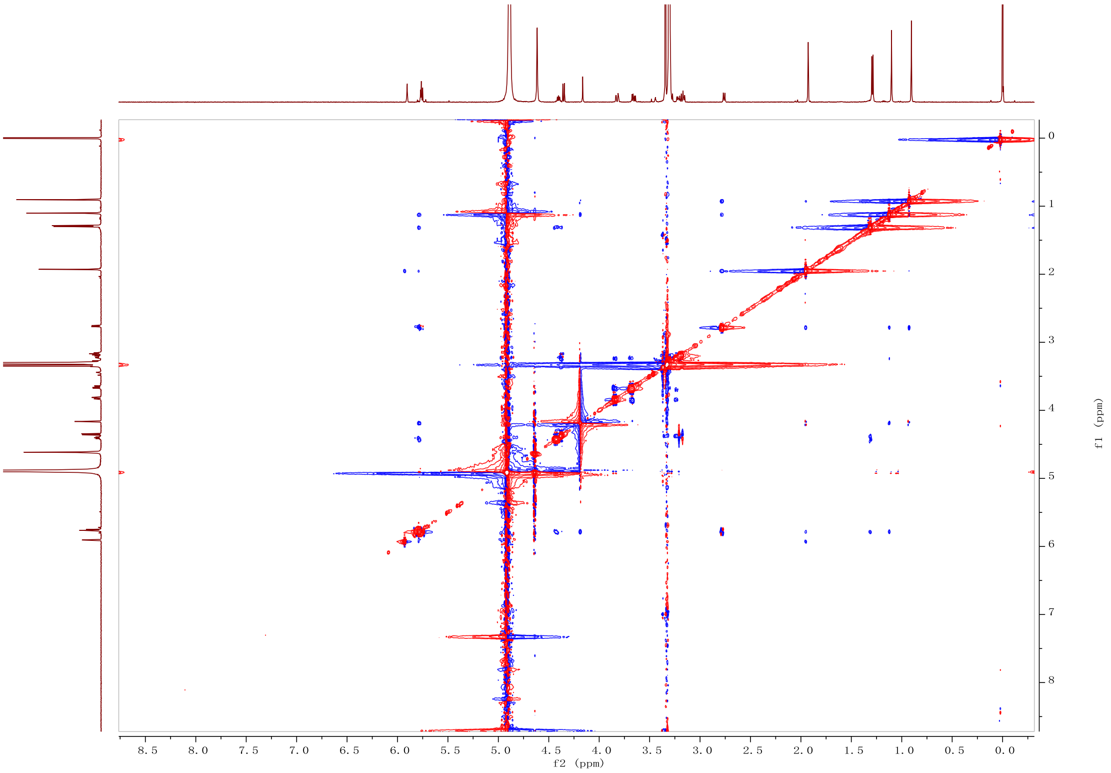

Figure S25: ROESY spectrum of compound **4**

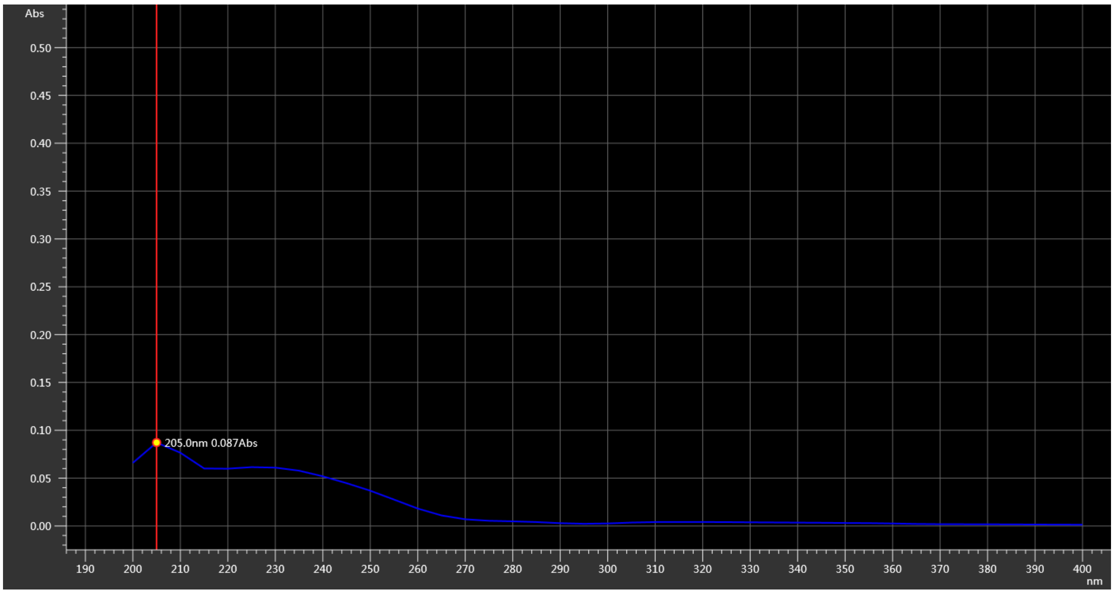

Figure S26: UV spectrum of compound **4**

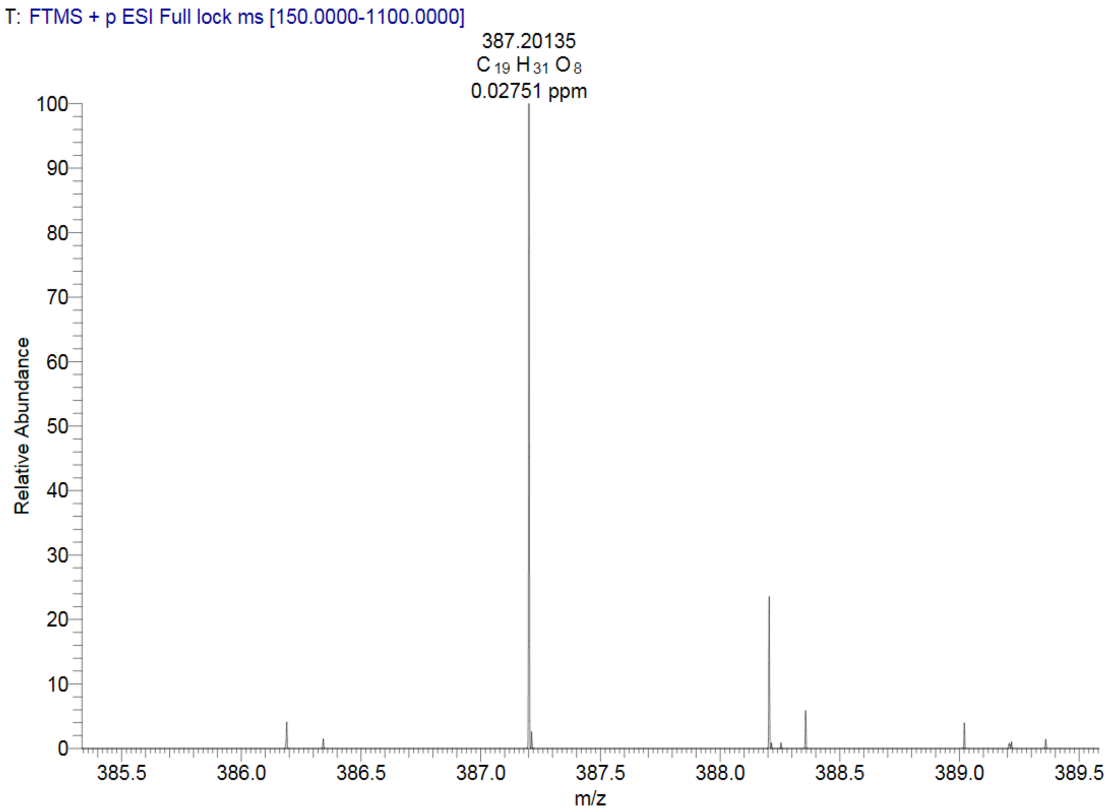

Figure S27: HR-ESI-MS of compound **4**

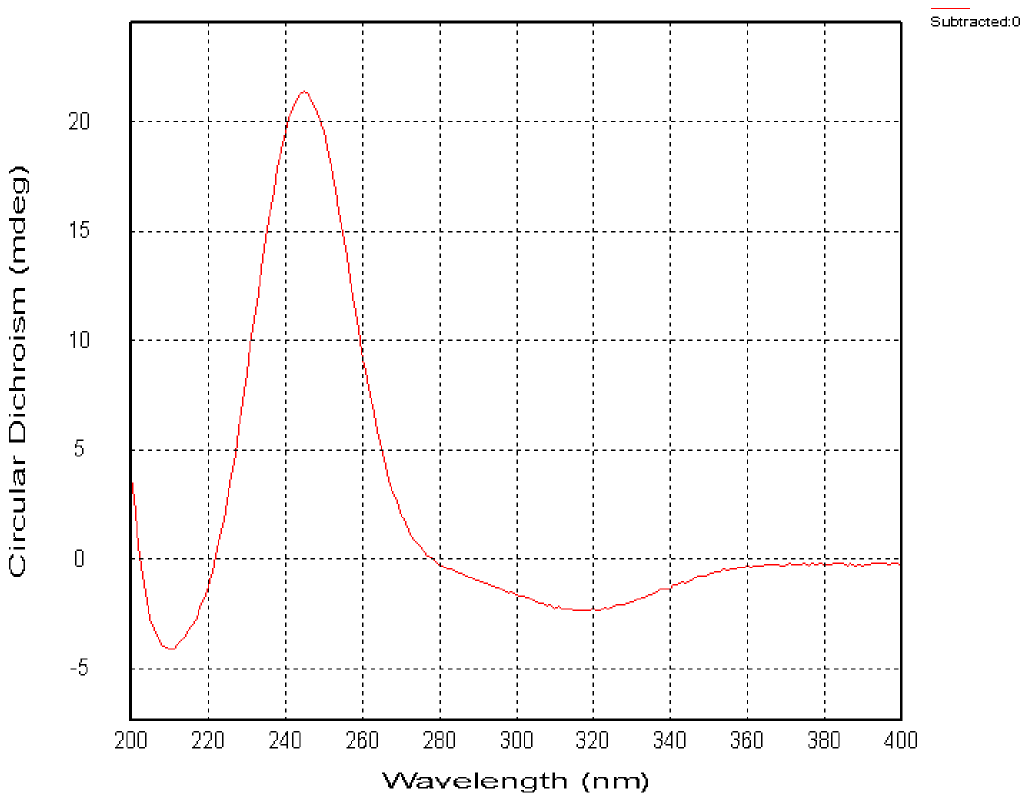

Figure S28: CD spectrum of compound **4**

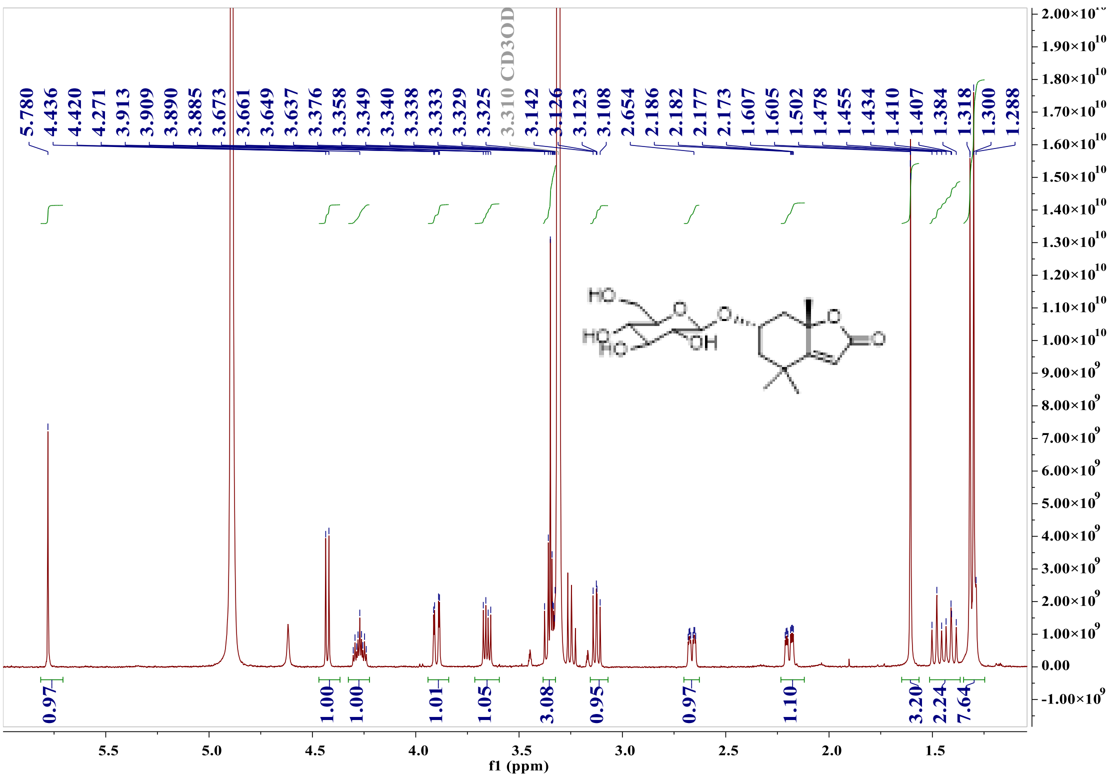

Figure S29: 1H-NMR (500 MHz, CD3OD) spectrum of compound 5

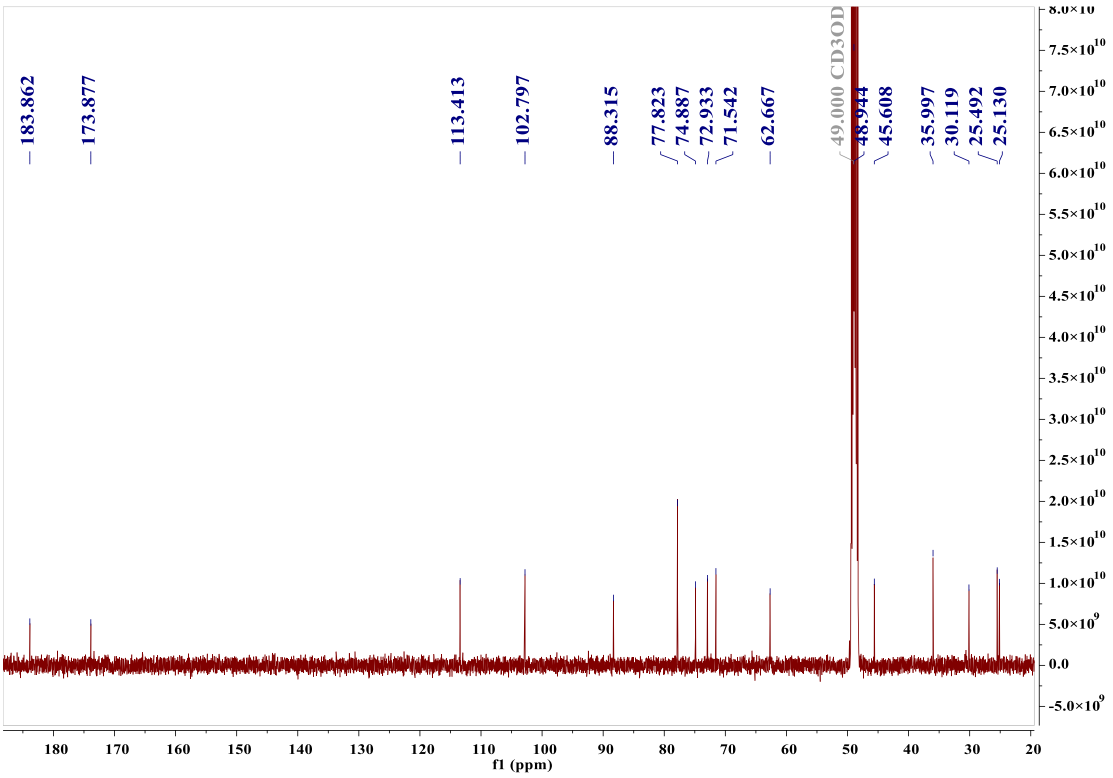

Figure S30: ^{13}C-NMR (125 MHz, CD_3OD) spectrum of compound **5**

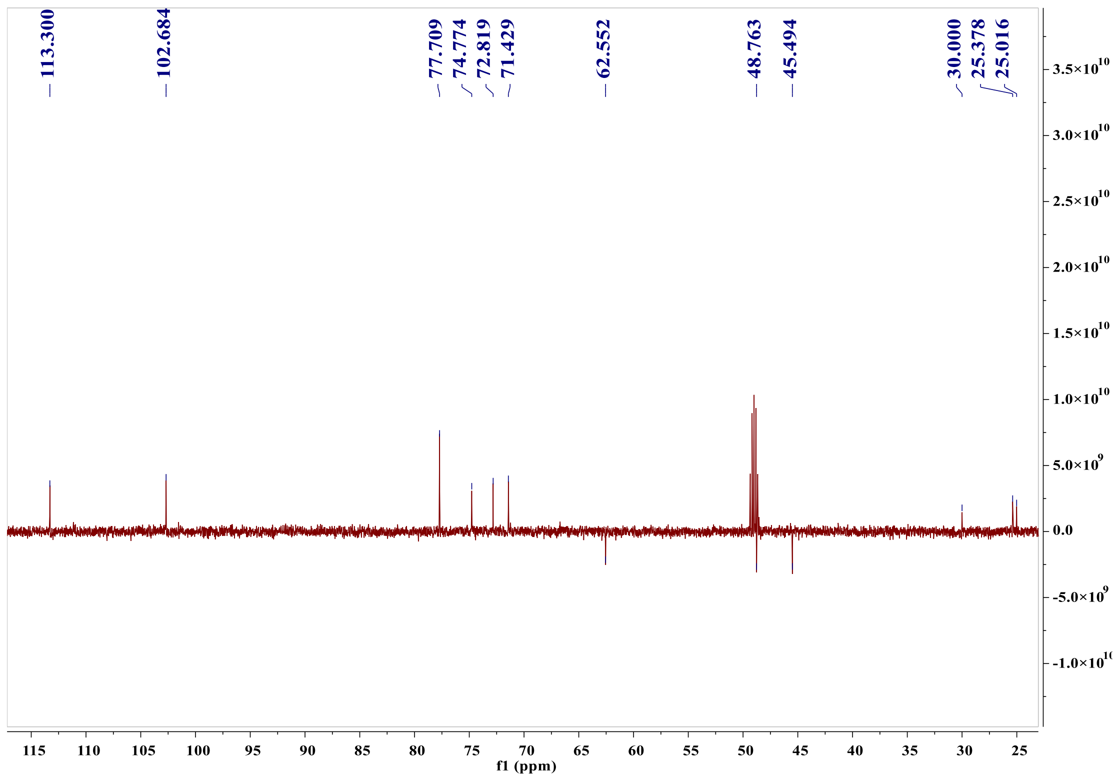

Figure S31: ^{13}C-NMR-DEPT (θ =135°) spectrum of compound **5**

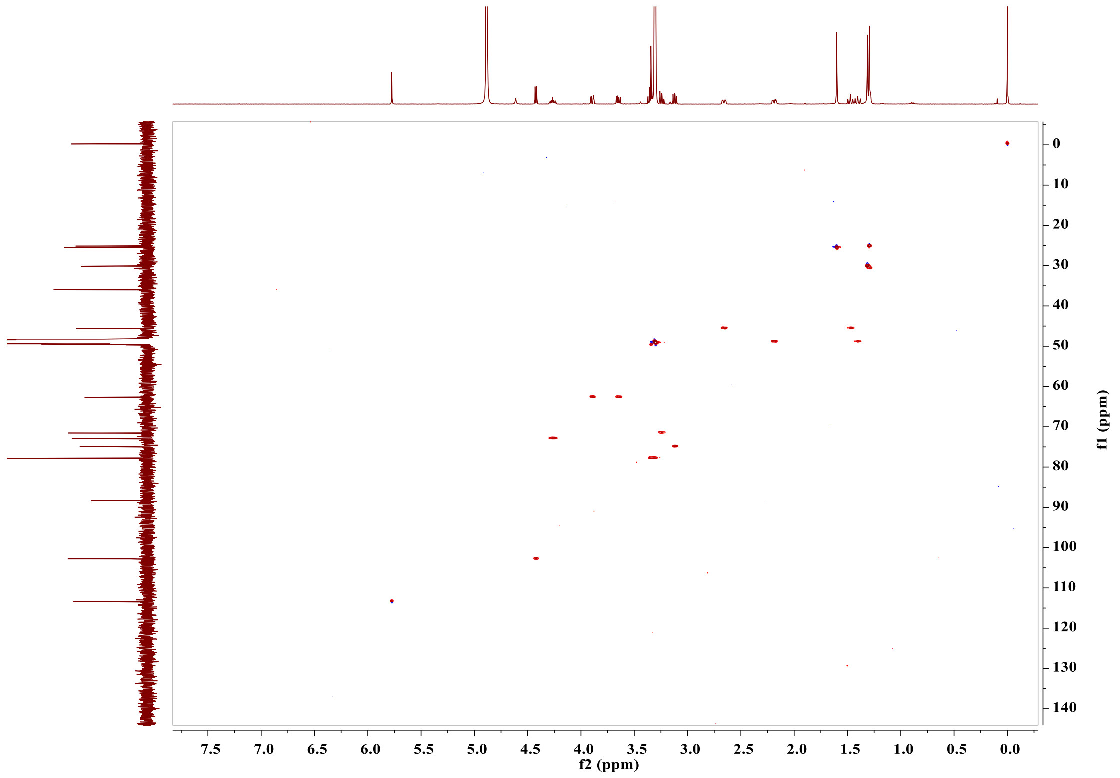

Figure S32: HSQC spectrum of compound **5**

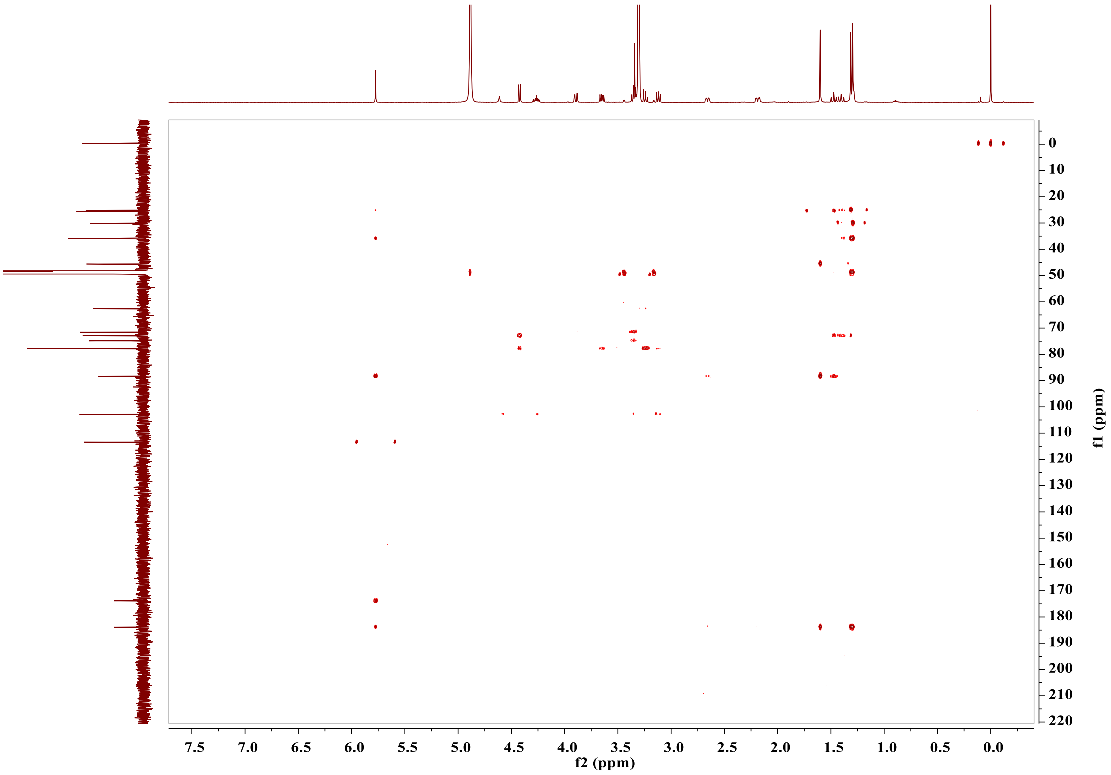

Figure S33: HMBC spectrum of compound **5**

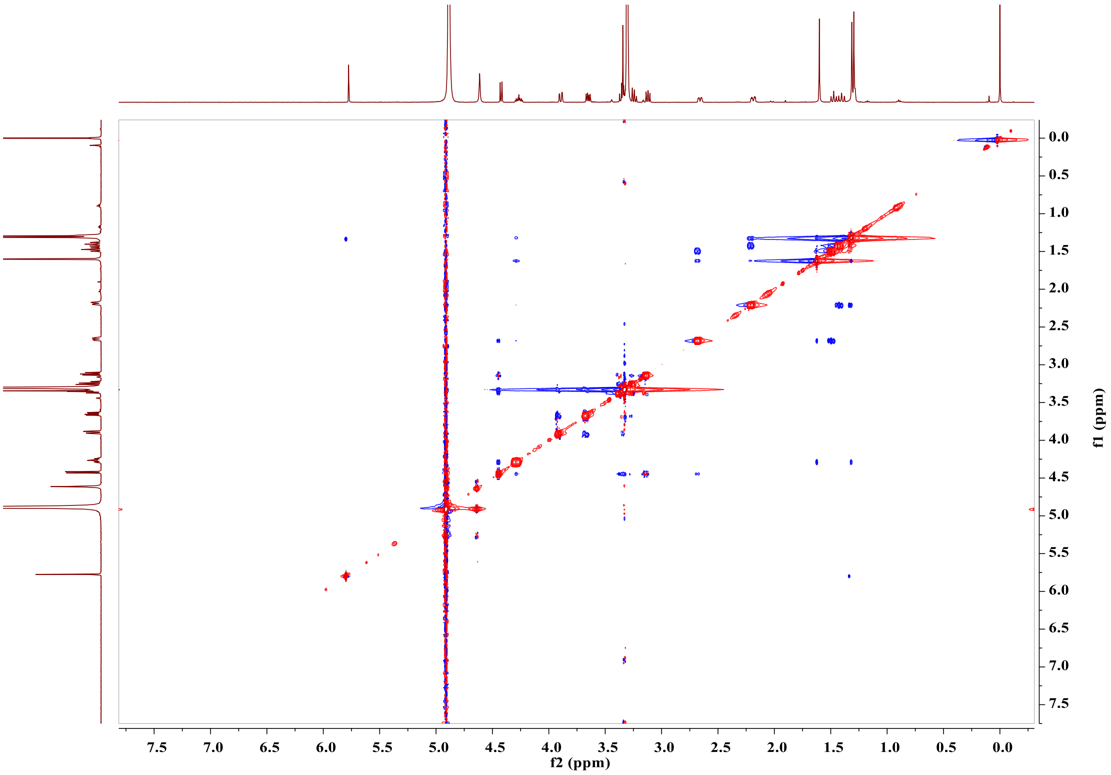

Figure S34: ROESY spectrum of compound **5**

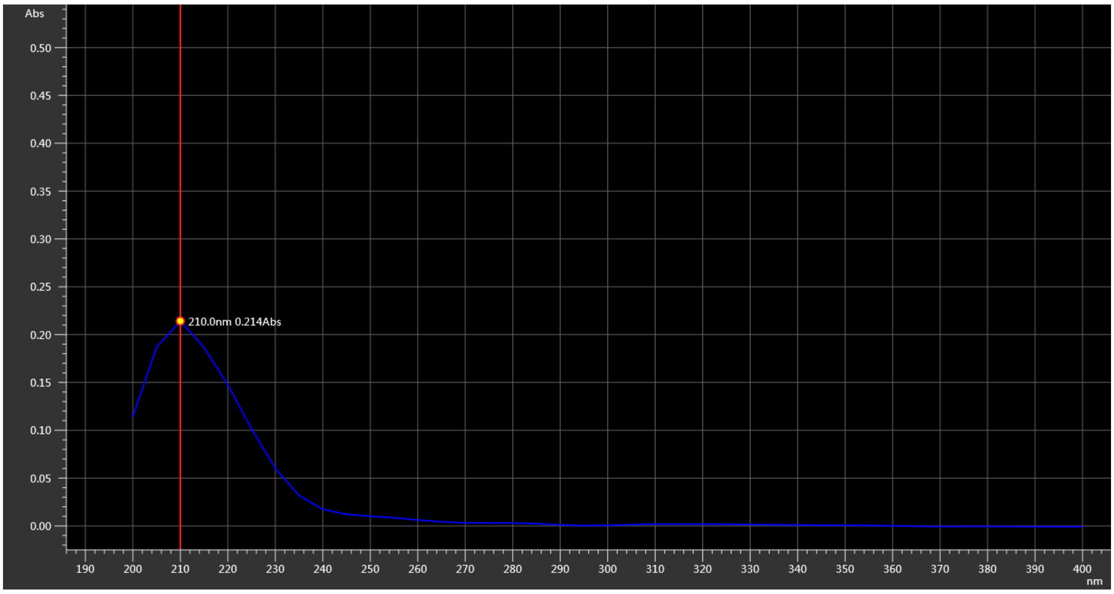

Figure S35: UV spectrum of compound **5**

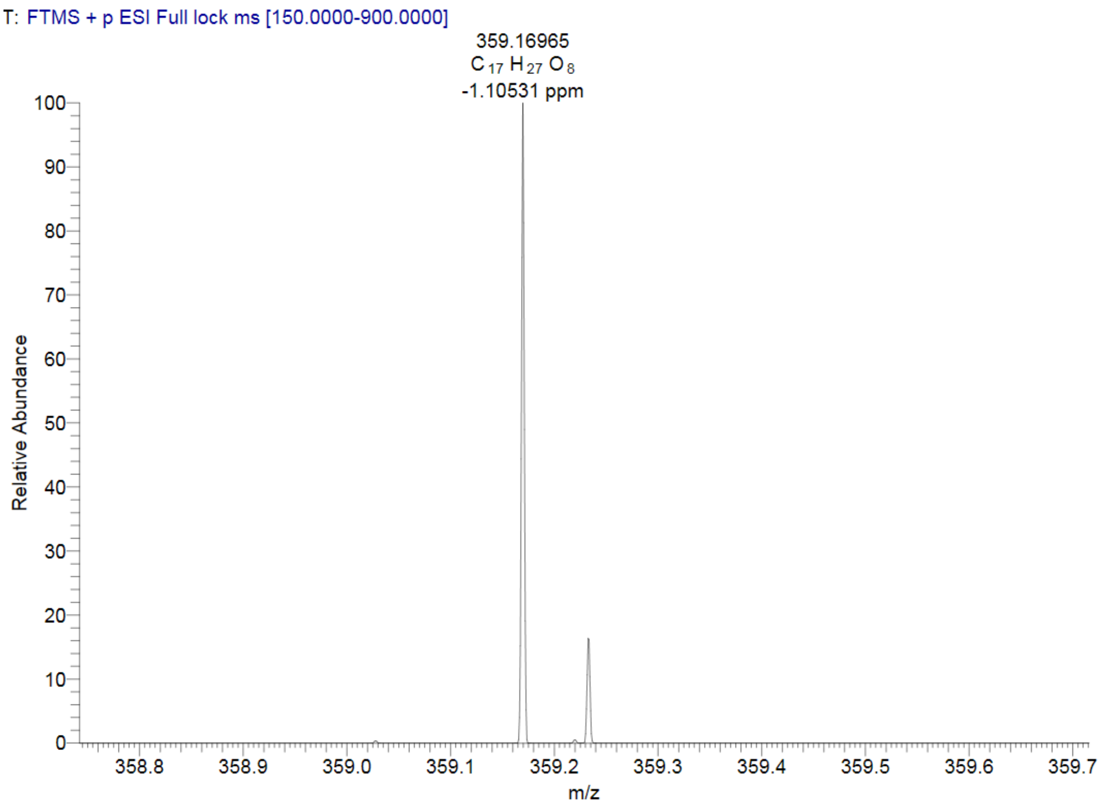

Figure S36: HR-ESI-MS of compound **5**

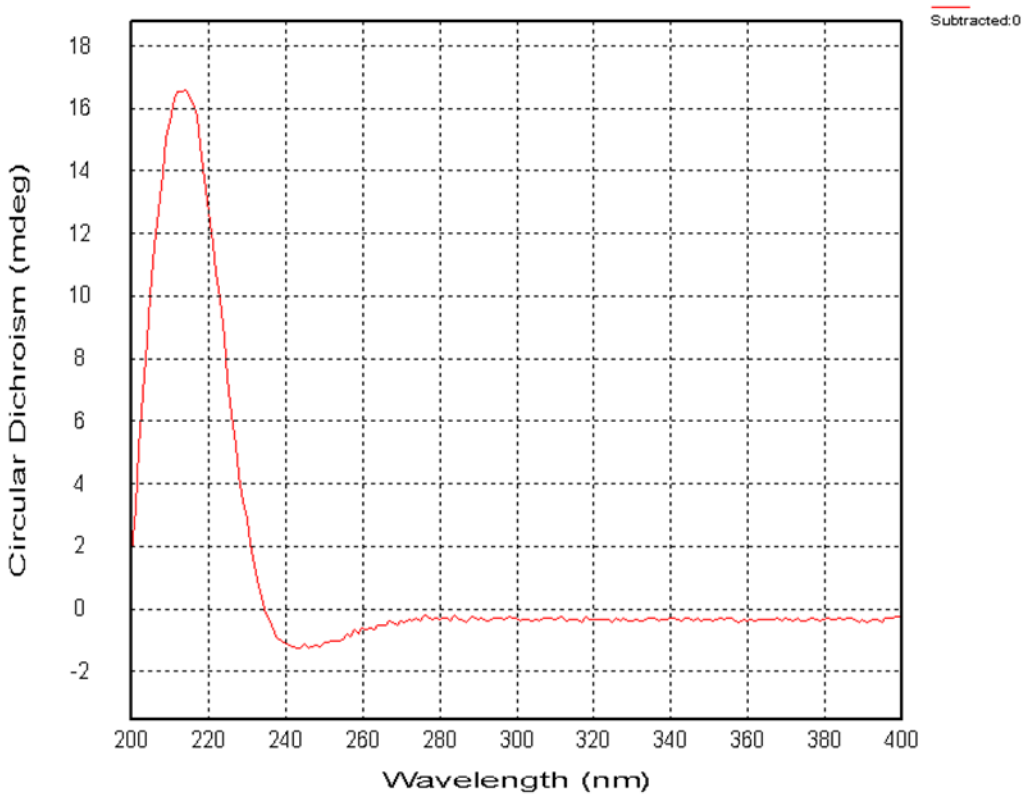

Figure S37: CD spectrum of compound **5**

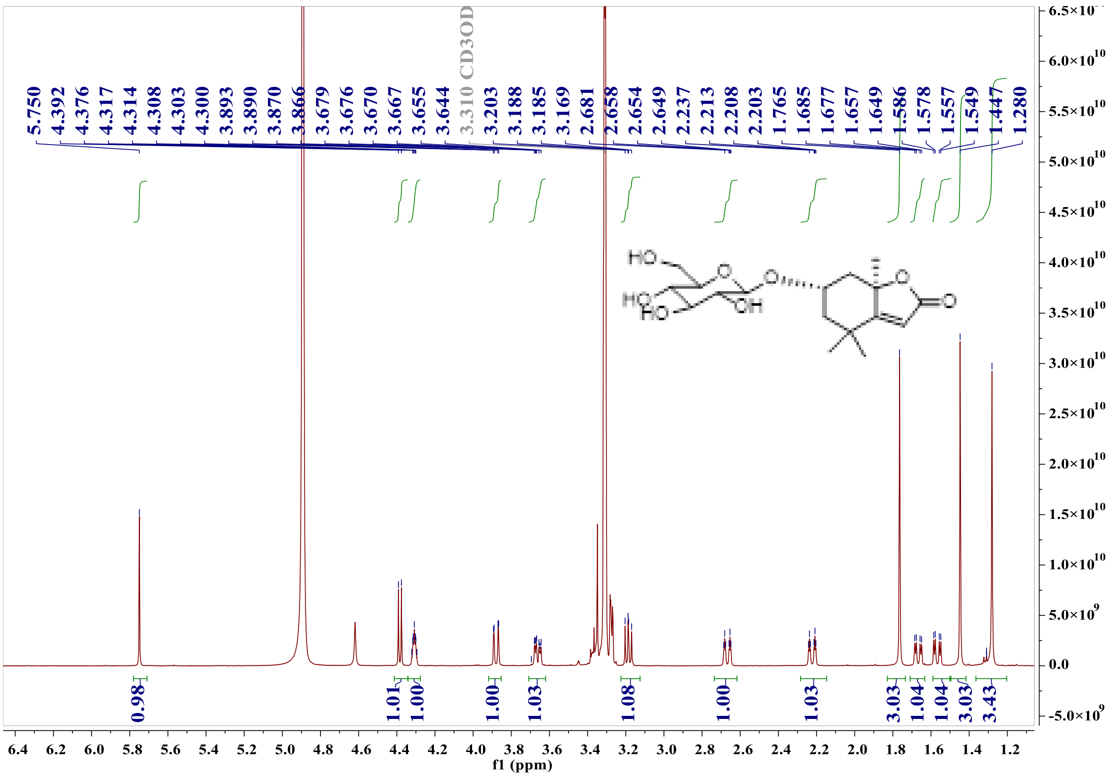

Figure S38: 1H-NMR (500 MHz, CD3OD) spectrum of compound **6a**

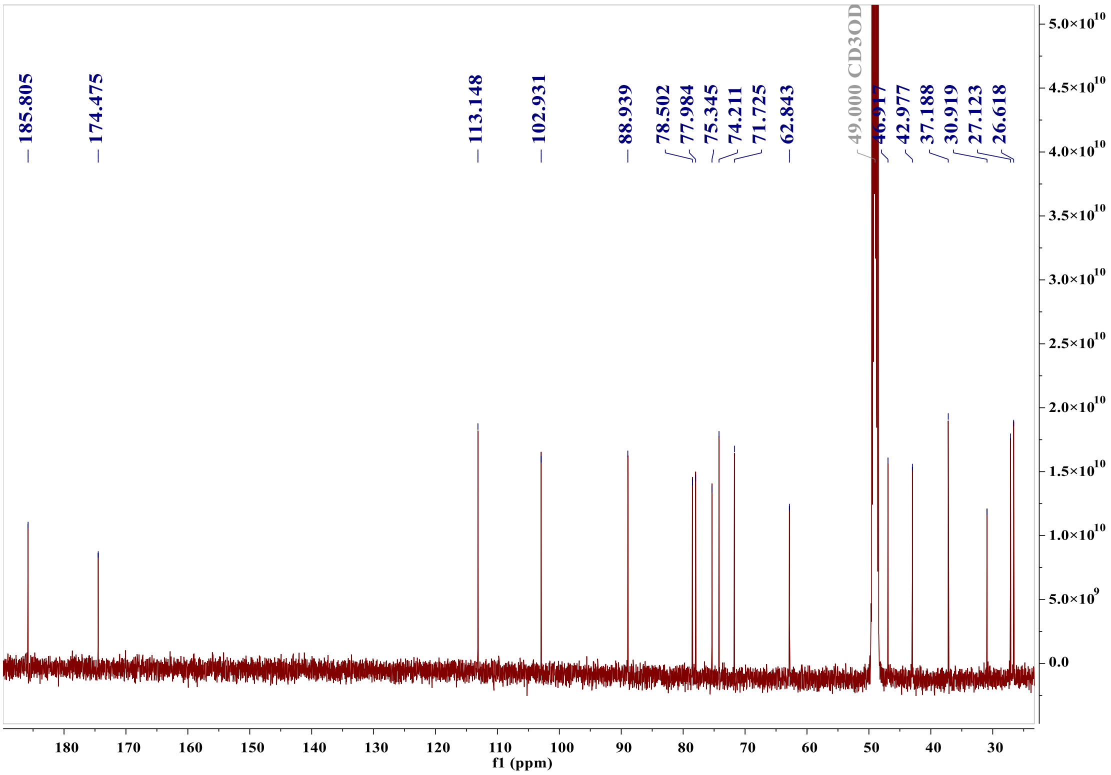

Figure S39: ^{13}C-NMR (125 MHz, CD_3OD) spectrum of compound **6a**

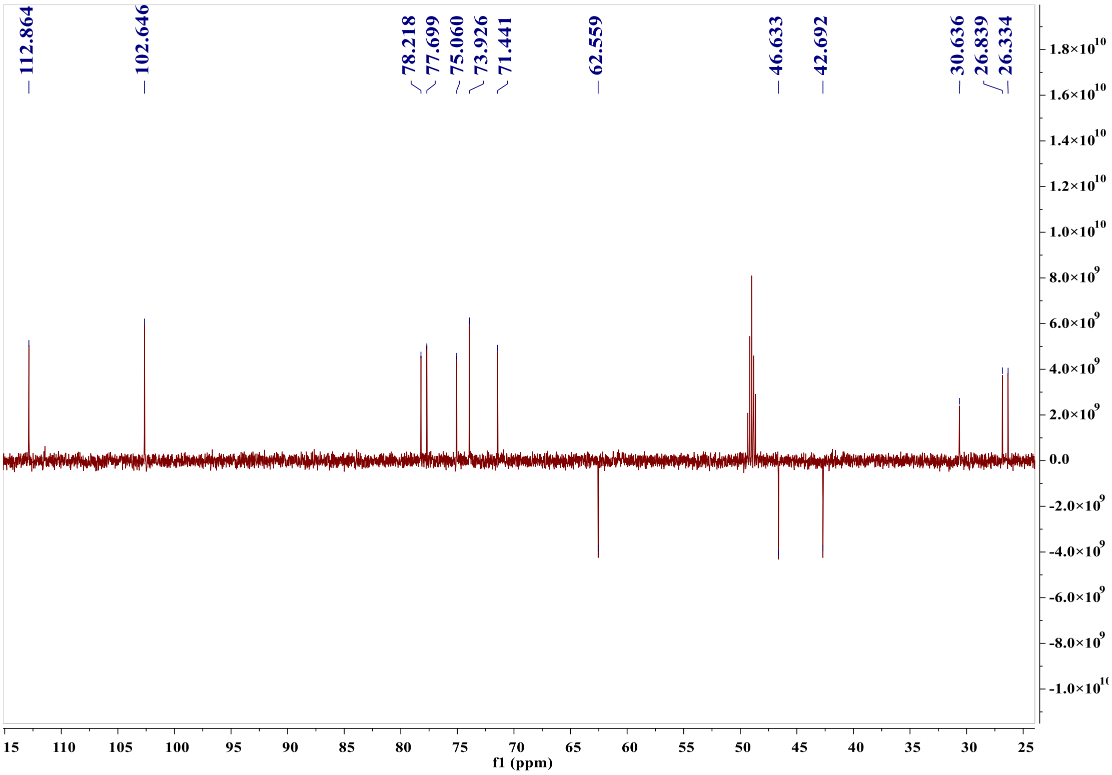

Figure S40: ^{13}C-NMR-DEPT (θ =135°) spectrum of compound **6a**

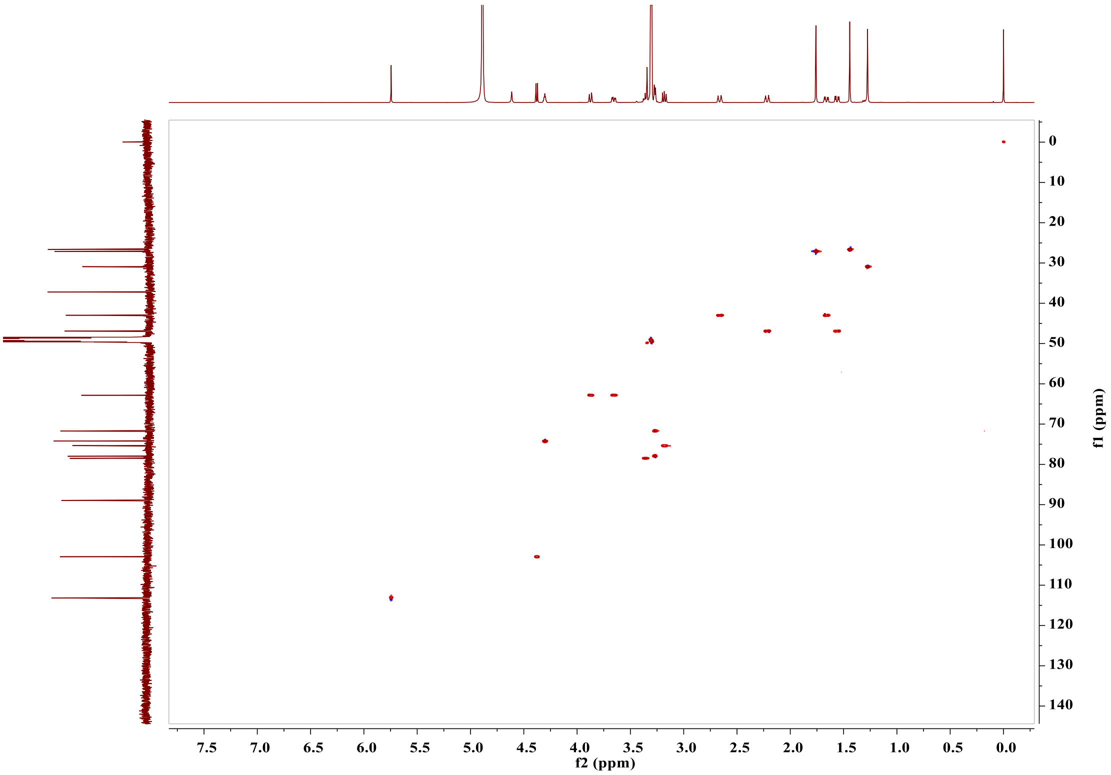

Figure S41: HSQC spectrum of compound **6a**

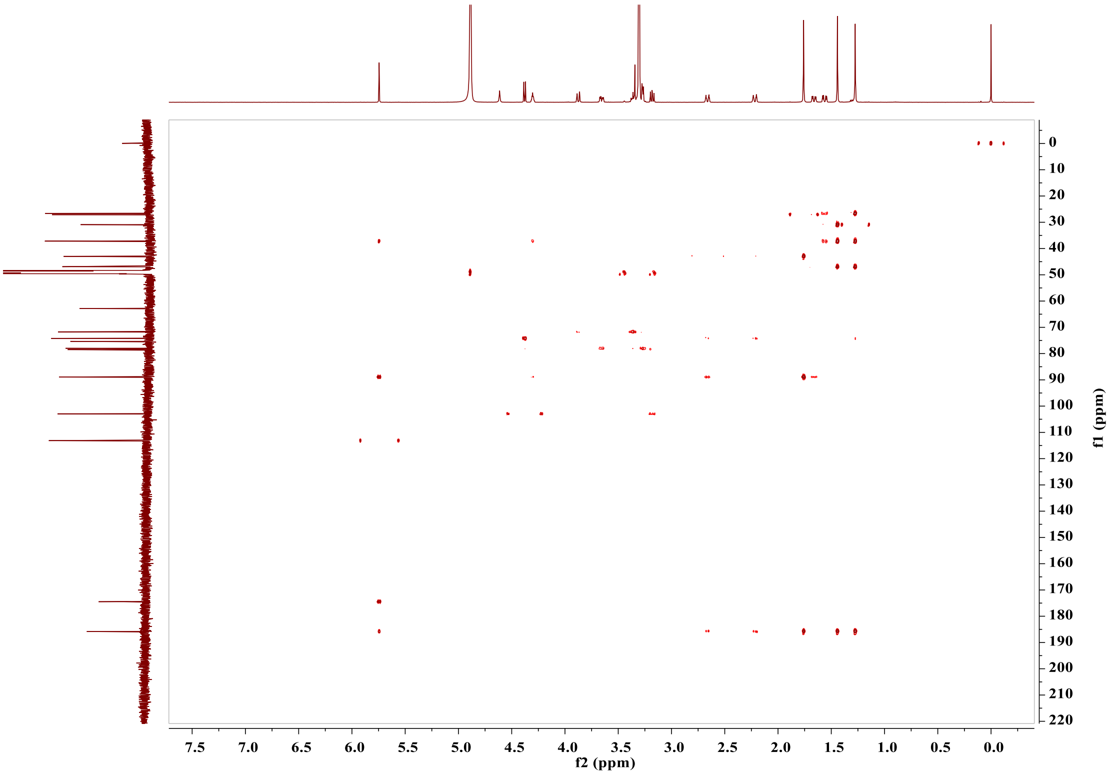

Figure S42: HMBC spectrum of compound **6a**

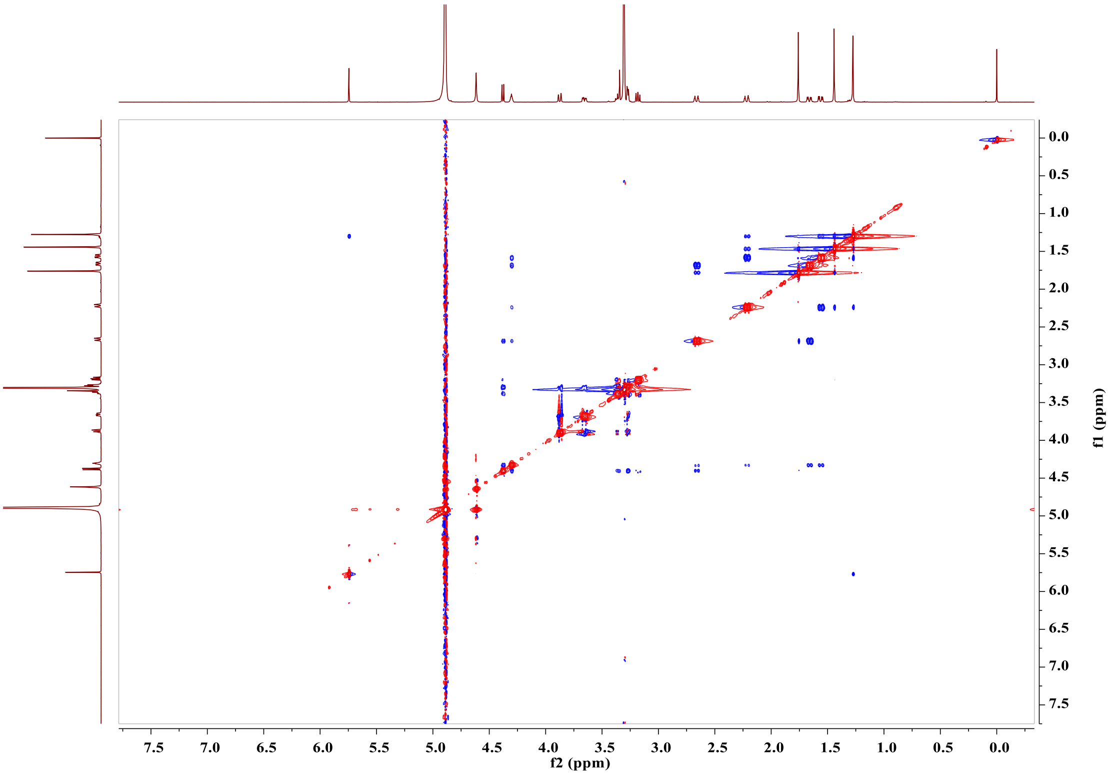

Figure S43: ROESY spectrum of compound **6a**

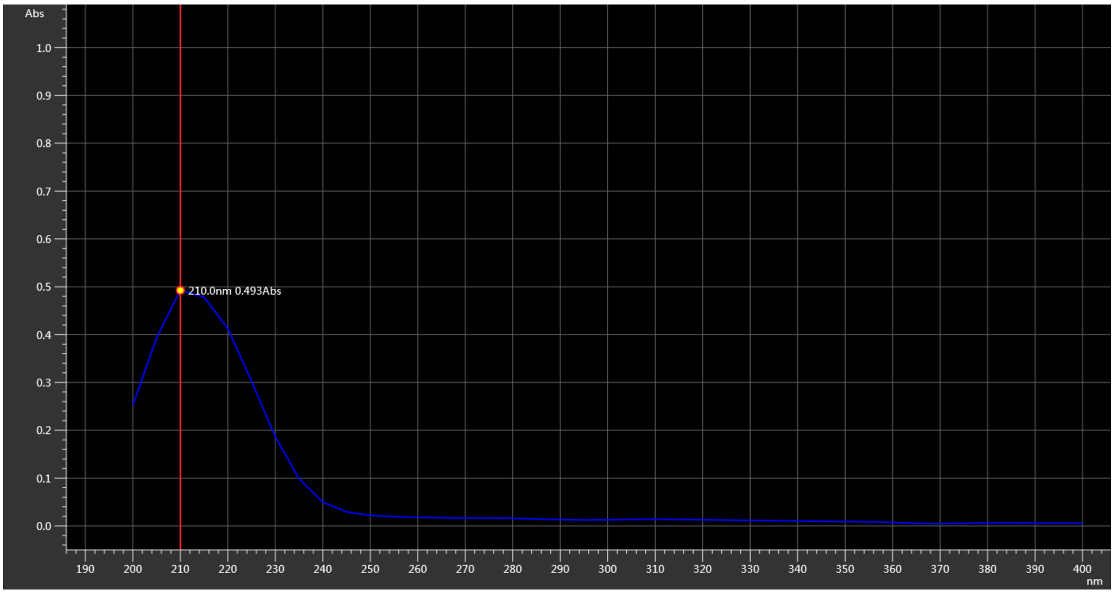

Figure S44: UV spectrum of compound **6a**

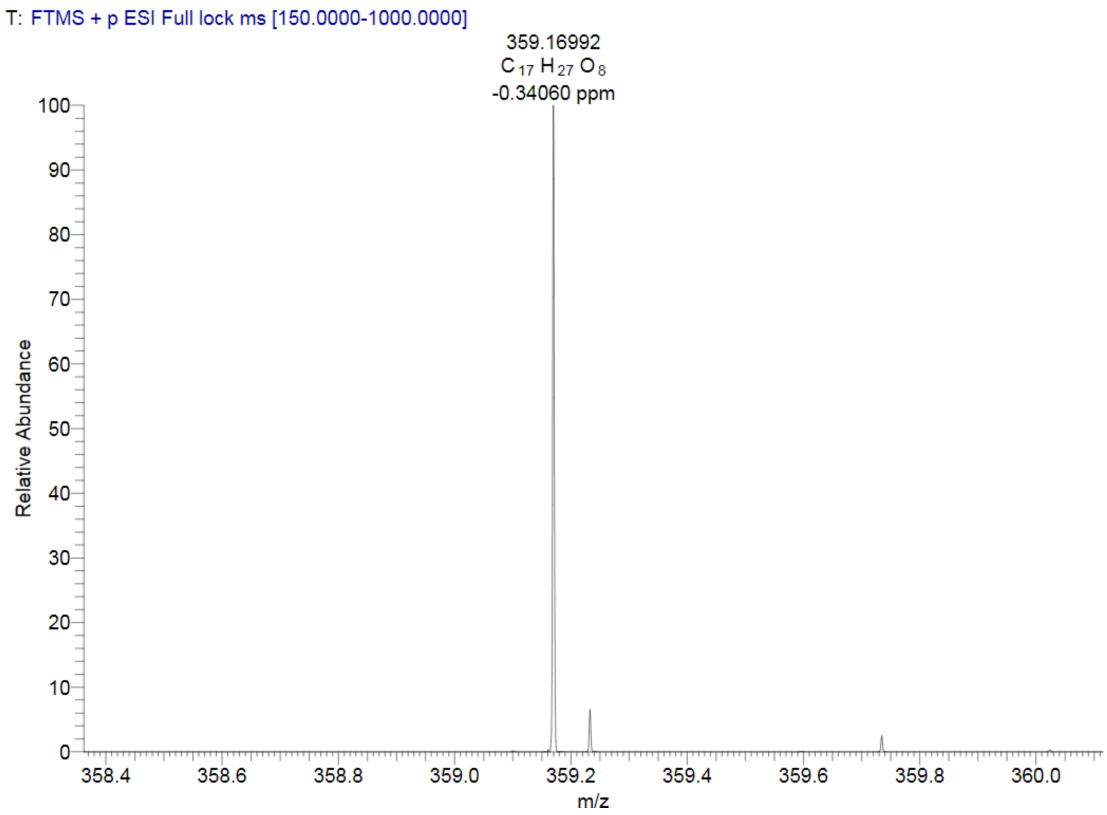

Figure S45: HR-ESI-MS of compound **6a**

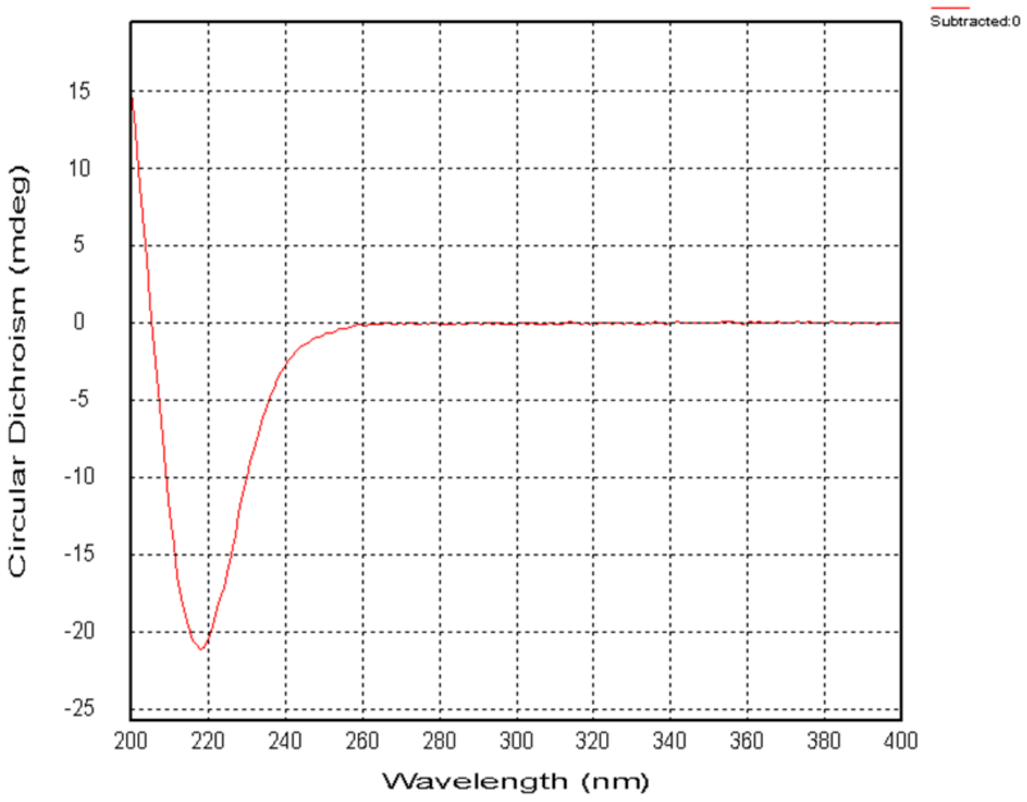

Figure S46: CD spectrum of compound **6a**

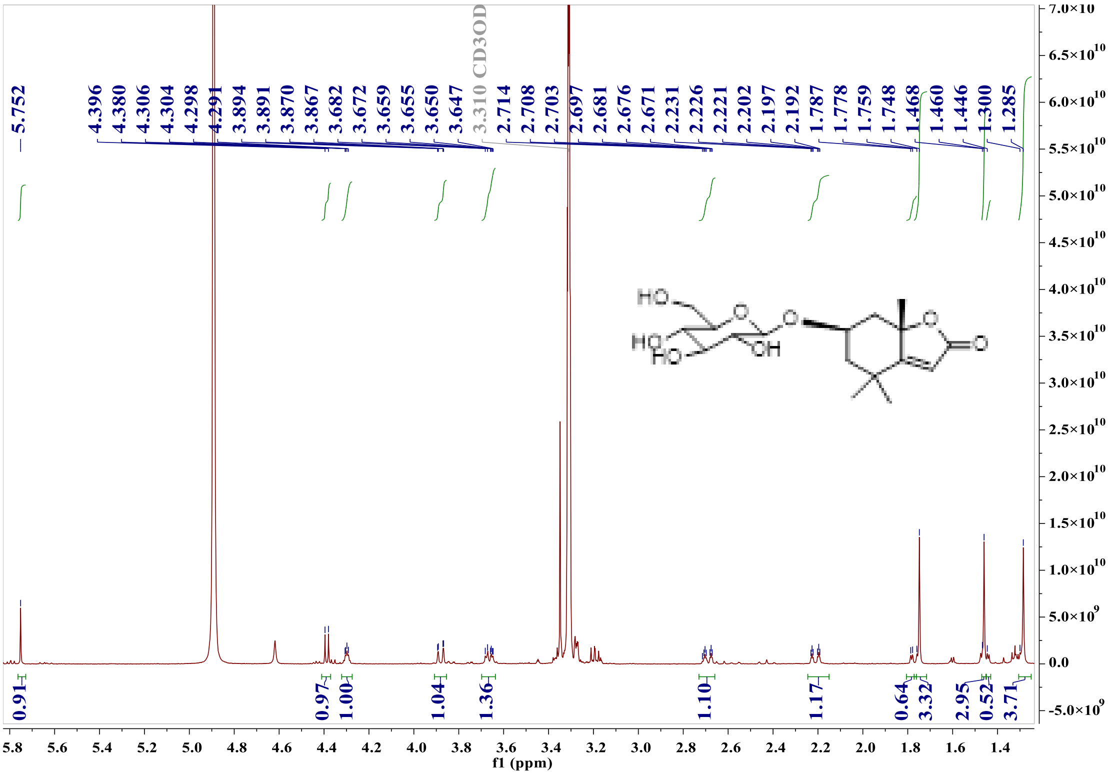

Figure S47: 1H-NMR (500 MHz, CD3OD) spectrum of compound **6b**

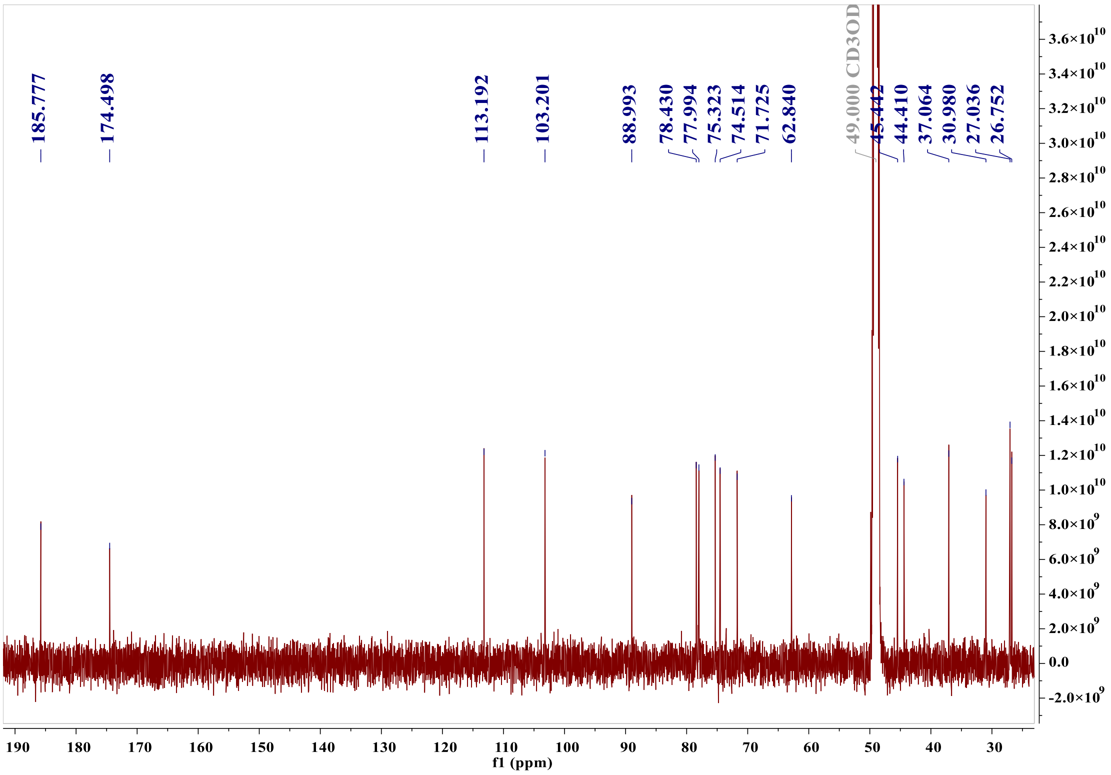

Figure S48: ^{13}C-NMR (125 MHz, CD3OD) spectrum of compound **6b**

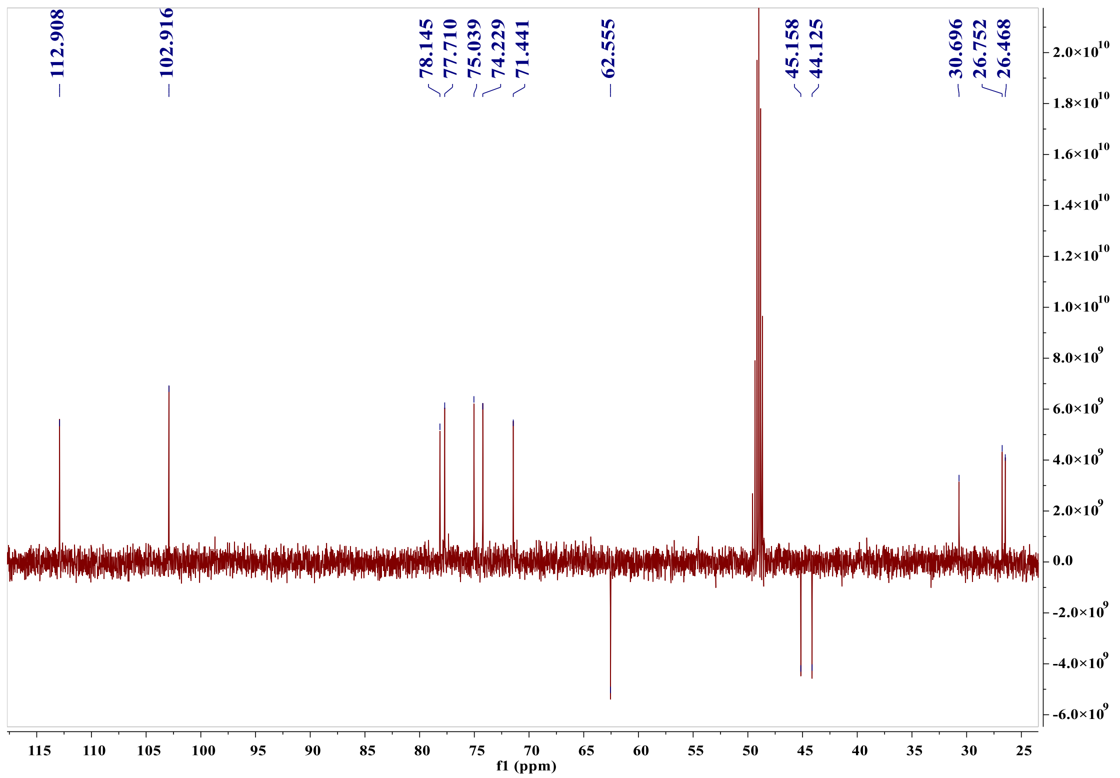

Figure S49: ^{13}C-NMR-DEPT (θ =135°) spectrum of compound **6b**

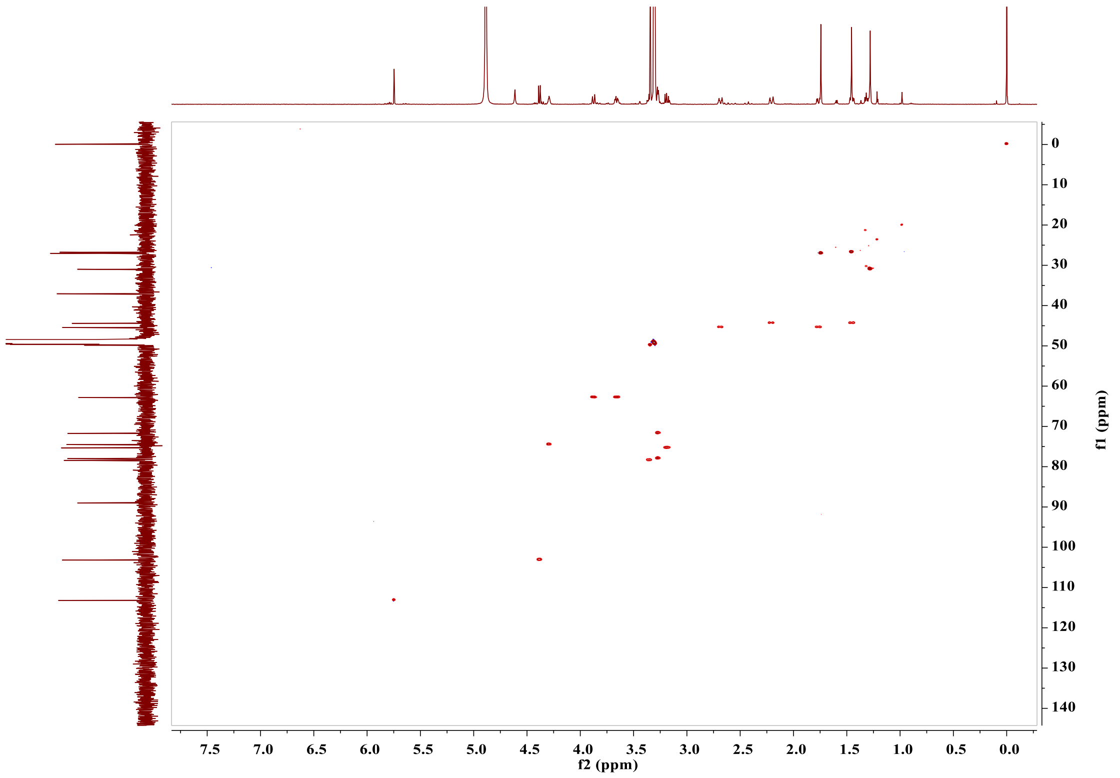

Figure S50: HSQC spectrum of compound **6b**

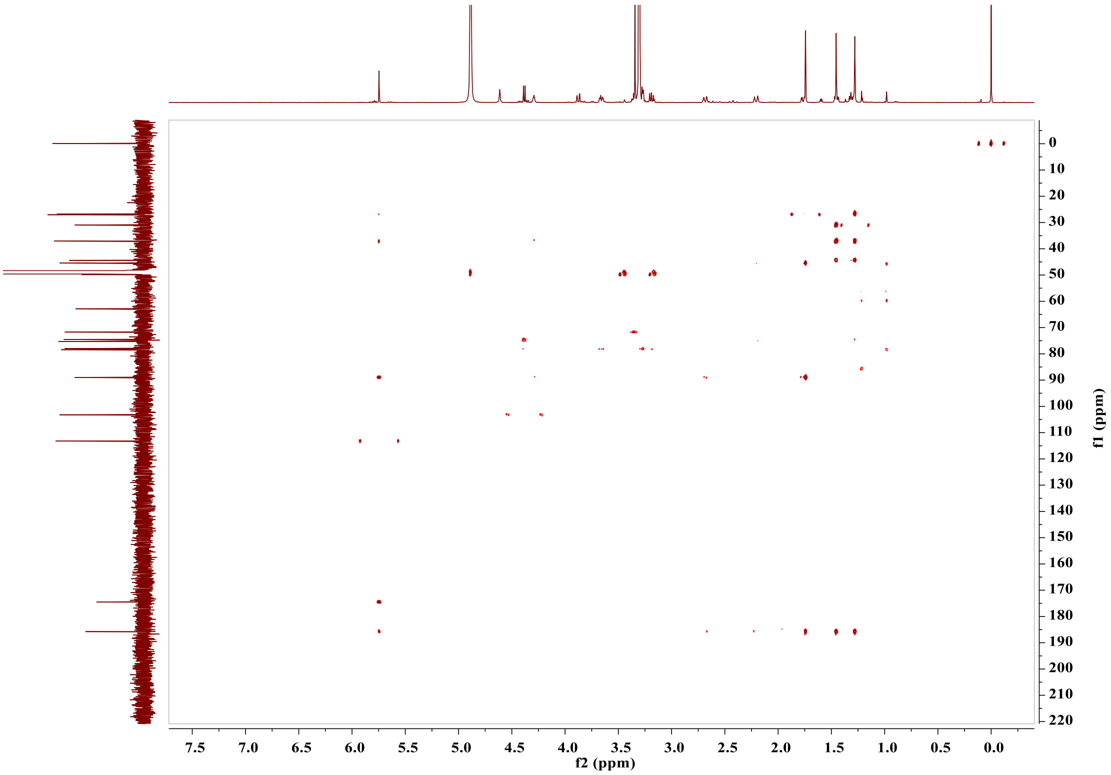

Figure S51: HMBC spectrum of compound **6b**

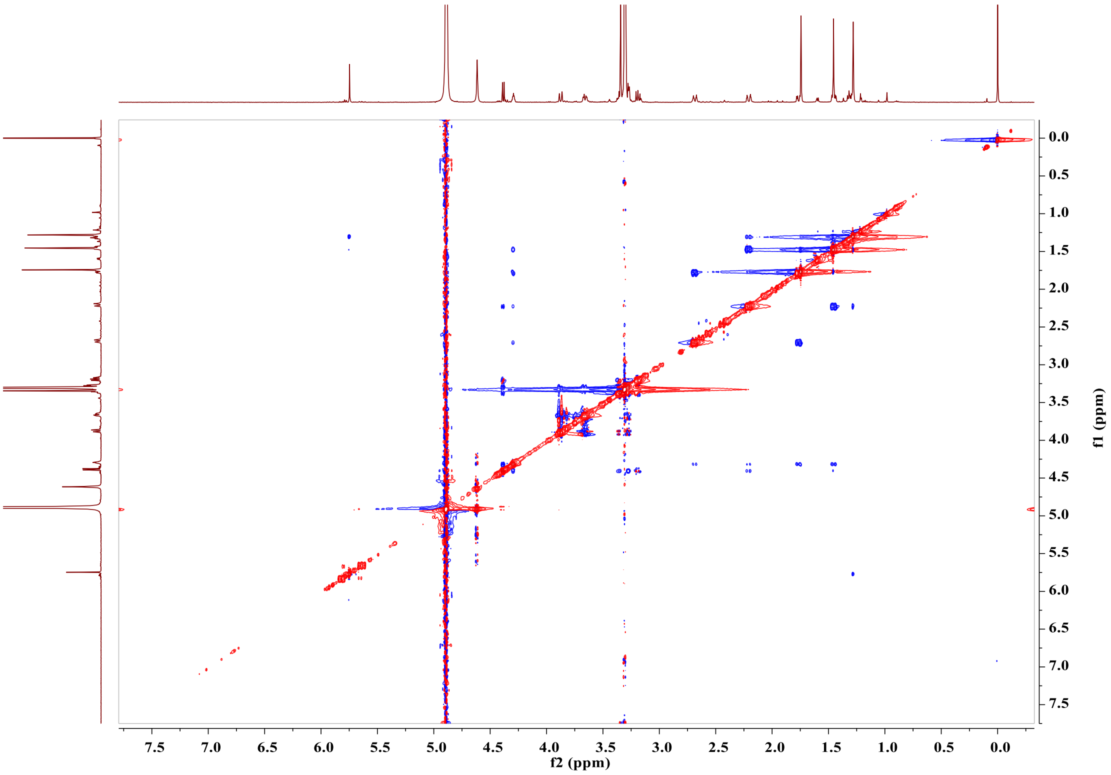

Figure S52: ROESY spectrum of compound **6b**

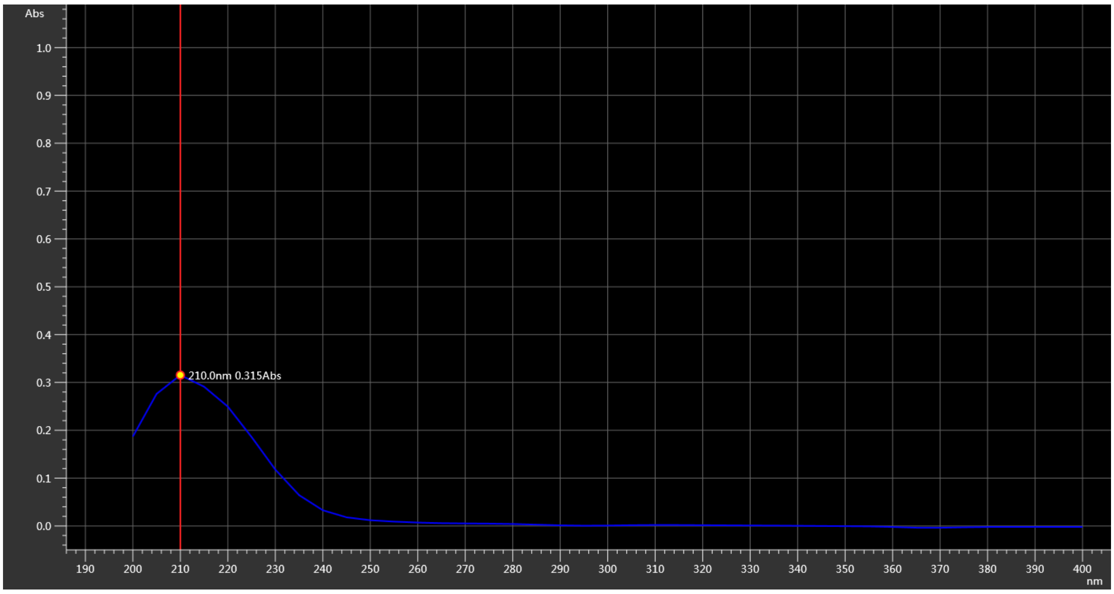

Figure S53: UV spectrum of compound **6b**

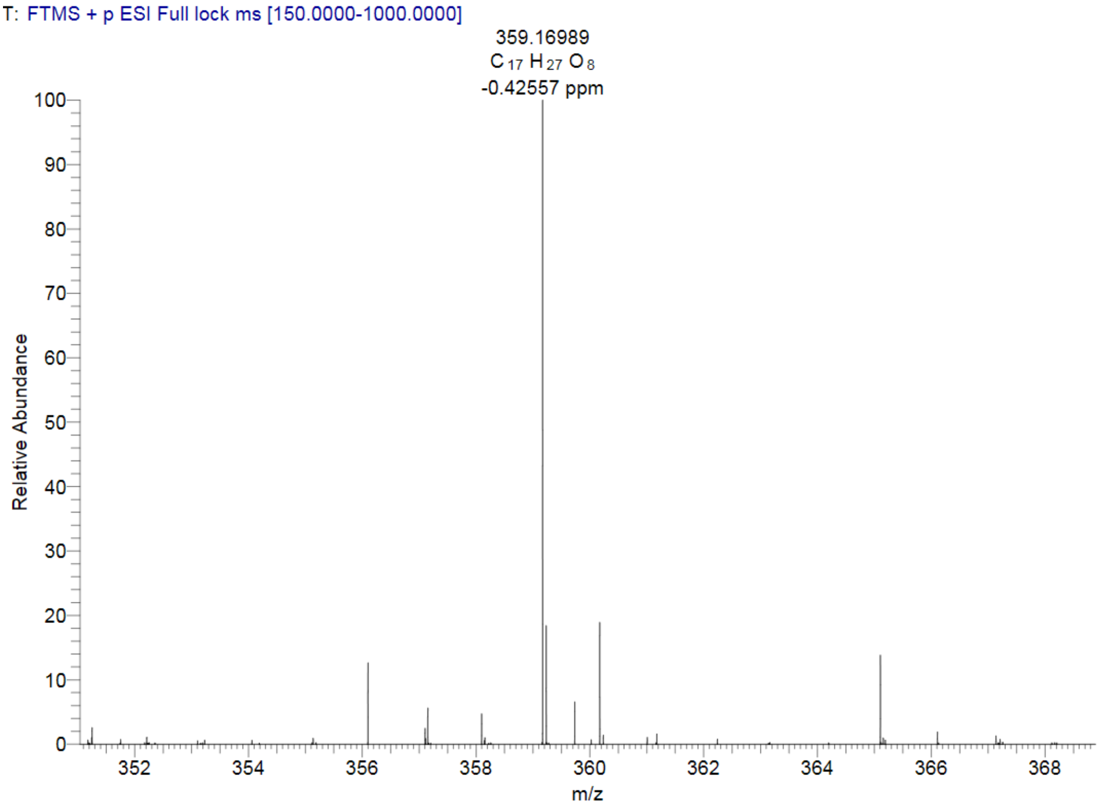

Figure S54: HR-ESI-MS of compound **6b**

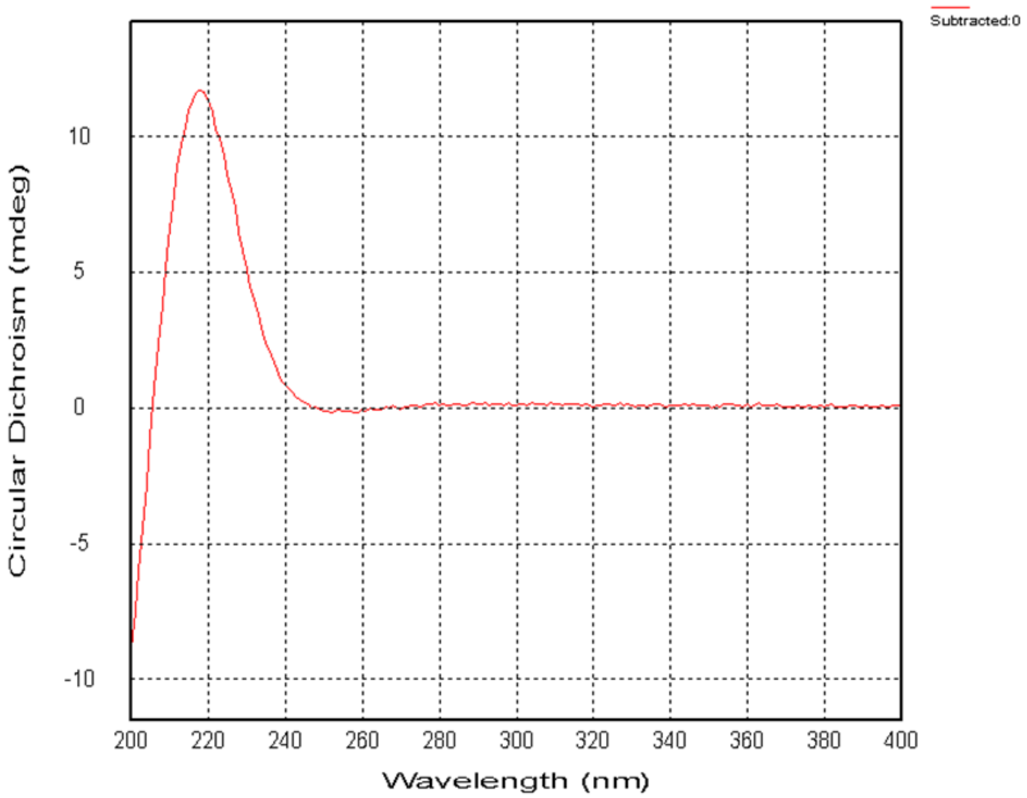

Figure S55: CD spectrum of compound **6b**

Computational NMR calculation of compound 3

Conformer	Energy (kcal/mol)	Population (%)
1	160.31	53.8
2	161.04	15.7
3	161.19	12.2
4	161.65	5.6
5	162.11	2.6
6	162.23	2.1
7	162.26	2
8	162.41	1.6
9	162.62	1.1
10	162.62	1.1

Table.S 1 Small molecule configuration search results of **3 (2*R*,6*R*,9*S*)**

Table.S 2 Geometric optimization at PM6 theoretical level of **3 (2*R*,6*R*,9*S*)**

Conformer	Energy (Hartree)	Energy (kcal/mol)	Population (%)
4	-0.60933	-382.36	66.8
7	-0.60819	-381.65	20.1
2	-0.60779	-381.39	13
1	-0.60285	-378.3	0.1
10	-0.60156	-377.49	0

Table.S 3 Geometric optimization at HF/6-31G(d) theoretical level of **3 (2*R*,6*R*,9*S*)**

Conformer	Energy (Hartree)	Energy (kcal/mol)	Population (%)
2	-1336.97	-838960	73.3
7	-1336.97	-838959	26.6

Table.S 4 Geometric optimization at B3LYP/6-31G(d) theoretical level of **3 (2*R*,6*R*,9*S*)**

Conformer	Energy (Hartree)	Energy (kcal/mol)	Population (%)
2	-1345.13	-844082	54.6
7	-1345.13	-844082	45.4

Table.S 5 Energy of structure for NMR calculation

	Conformer	Energy (Hartree)	Energy (kcal/mol)	Population (%)
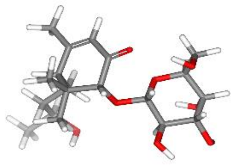	2	-1345.13	-844082	54.6
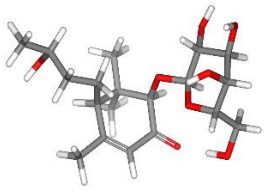	7	-1345.13	-844082	45.4

Table.S 6 Standard orientations at mPW1PW91/6-311G(2d,p) level in Methanol. of **3 (2*R*,6*R*,9*S*)**

Conformer 2			
Center	Atomic	Atomic	Coordinates (Angstroms)

Number	Number	Type	X	Y	Z
1	6	0	0.305684	-1.36719	1.066598
2	6	0	-0.00012	-2.41619	-0.0071
3	6	0	1.019677	-2.6743	-1.0267
4	6	0	2.22728	-2.1173	-0.988
5	6	0	2.634986	-1.09671	0.061998
6	6	0	1.808085	-1.2575	1.374398
7	6	0	2.628594	0.322294	-0.586
8	8	0	-1.05053	-3.00288	0.003898
9	6	0	3.863899	1.163587	-0.2524
10	6	0	3.806907	2.568987	-0.8536
11	8	0	2.680512	3.279994	-0.3931
12	6	0	5.085712	3.36018	-0.5996
13	6	0	-1.53481	-0.02028	0.425598
14	8	0	-1.70371	-0.09488	-0.9684
15	6	0	-3.01961	-0.06017	-1.4833
16	6	0	-3.7505	1.178832	-0.955
17	6	0	-3.5682	1.36723	0.548098
18	6	0	-2.0923	1.291622	0.934998
19	8	0	-1.9463	1.329021	2.323198
20	8	0	-4.11489	2.585334	0.977598
21	8	0	-3.28049	2.337229	-1.619
22	6	0	-3.74032	-1.40077	-1.361
23	6	0	3.280078	-2.48321	-2.0008

24	6	0	2.242577	-2.5573	2.085198
25	6	0	2.041392	-0.1035	2.360398
26	8	0	-3.92102	-1.86137	-0.0525
27	8	0	-0.18021	-0.10969	0.661998
28	1	0	-0.22232	-1.67419	1.963498
29	1	0	0.746873	-3.3817	-1.7894
30	1	0	3.667085	-1.32501	0.321998
31	1	0	1.730997	0.849399	-0.3093
32	1	0	2.581794	0.220194	-1.6662
33	1	0	4.758096	0.662782	-0.6198
34	1	0	3.9885	1.251986	0.824698
35	1	0	3.655107	2.486088	-1.924
36	1	0	2.759312	3.423993	0.540998
37	1	0	5.268613	3.469079	0.467398
38	1	0	5.003718	4.35168	-1.0303
39	1	0	5.948509	2.865675	-1.0346
40	1	0	-2.04821	-0.82498	0.921698
41	1	0	-2.89161	0.101126	-2.5482
42	1	0	-4.8061	1.120138	-1.1848
43	1	0	-4.0962	0.584934	1.077298
44	1	0	-1.5394	2.108219	0.474298
45	1	0	-2.5102	2.016924	2.655598
46	1	0	-3.91519	3.240332	0.318198
47	1	0	-2.34239	2.268423	-1.7469

48	1	0	-4.72042	-1.30256	-1.816
49	1	0	-3.17812	-2.12447	-1.9404
50	1	0	3.716783	-1.60041	-2.4561
51	1	0	2.879874	-3.11161	-2.7864
52	1	0	4.087975	-3.02071	-1.5104
53	1	0	3.292478	-2.50241	2.353998
54	1	0	1.678777	-2.6996	3.001698
55	1	0	2.106572	-3.4412	1.473098
56	1	0	1.750097	0.853699	1.954998
57	1	0	1.457191	-0.2631	3.261198
58	1	0	3.086892	-0.05981	2.650598
59	1	0	-3.19612	-2.43477	0.169498

Conformer 7					
Center	Atomic	Atomic	Coordinates (Angstroms)		
Number	Number	Type	X	Y	Z
1	6	0	-0.2163	1.038196	1.207203
2	6	0	0.089107	2.251895	0.334503
3	6	0	-0.82679	2.527398	-0.7749
4	6	0	-1.9709	1.866802	-0.939
5	6	0	-2.4199	0.737103	-0.0205
6	6	0	-1.7239	0.817101	1.374903
7	6	0	-2.287	-0.6219	-0.7804
8	8	0	1.058509	2.927692	0.556603
9	6	0	-3.5859	-1.20049	-1.3685

10	6	0	-4.52951	-1.87059	-0.3651
11	8	0	-5.1031	-0.94259	0.532203
12	6	0	-5.62191	-2.67869	-1.0581
13	6	0	1.721999	-0.17051	0.528503
14	8	0	2.0856	0.292989	-0.7451
15	6	0	3.467001	0.313185	-1.0611
16	6	0	4.012996	-1.10932	-0.952
17	6	0	3.665794	-1.73302	0.396803
18	6	0	2.172694	-1.61071	0.694003
19	8	0	1.897293	-2.01321	2.001703
20	8	0	4.05409	-3.07822	0.455203
21	8	0	3.482193	-1.92032	-1.9788
22	6	0	4.242304	1.377882	-0.2867
23	6	0	-2.88429	2.214305	-2.086
24	6	0	-1.9734	-0.4297	2.239103
25	6	0	-2.30979	2.019003	2.148403
26	8	0	3.800808	2.656884	-0.6042
27	8	0	0.350099	-0.10811	0.612003
28	1	0	0.246803	1.205195	2.173803
29	1	0	-0.53189	3.321097	-1.4378
30	1	0	-3.474	0.889907	0.174203
31	1	0	-1.5689	-0.5025	-1.5842
32	1	0	-1.85351	-1.3656	-0.1298
33	1	0	-4.1436	-0.44139	-1.9143

34	1	0	-3.30621	-1.95149	-2.1039
35	1	0	-3.95181	-2.53589	0.265403
36	1	0	-5.7492	-0.42169	0.073203
37	1	0	-6.21571	-2.04629	-1.7155
38	1	0	-5.20041	-3.47649	-1.6606
39	1	0	-6.28521	-3.11798	-0.3216
40	1	0	2.169501	0.443389	1.301203
41	1	0	3.501401	0.606685	-2.1017
42	1	0	5.086896	-1.11942	-1.0885
43	1	0	4.209896	-1.22772	1.187103
44	1	0	1.612493	-2.21481	-0.0165
45	1	0	2.320191	-2.85081	2.146703
46	1	0	3.857989	-3.47192	-0.3877
47	1	0	2.595994	-1.63771	-2.1692
48	1	0	4.212403	1.204582	0.786103
49	1	0	5.282104	1.321779	-0.5871
50	1	0	-2.51879	3.072004	-2.6363
51	1	0	-2.971	1.383305	-2.7786
52	1	0	-3.88399	2.437808	-1.724
53	1	0	-1.6572	-0.2339	3.259403
54	1	0	-3.0305	-0.6701	2.257803
55	1	0	-1.42111	-1.2925	1.897703
56	1	0	-1.79439	2.156401	3.093903
57	1	0	-3.3554	1.834406	2.371103

58	1	0	-2.24809	2.951303	1.600003
59	1	0	2.927508	2.801586	-0.2544

Functional		Solvent?		Basis Set	
mPW1PW91		PCM		6-311G(d,p)	
		DP4+	0.00%	100.00%	-
Nuclei	sp2?	xperiments	Isomer 1	Isomer 2	Isomer 3
C		43.2	49.9	45.3	
C	x	77.2	97.0	89.4	
C		201.2	206.8	206.6	
C		124	130.8	127.8	
C		168.6	178.2	185.5	
C	x	54.5	53.1	54.1	
C		25.9	26.8	32.8	
C		38.2	41.3	44.7	
C	x	75.4	68.3	73.4	
C		24.6	14.7	27.3	
C		21	25.5	25.0	
C		24.5	25.67	26.66	
C		19.8	25.60	25.86	
C		102	112.09	104.74	
C		78.1	74.23	75.06	
C		71.8	65.79	77.66	
C		77.9	72.68	77.80	
C		75.1	85.67	84.84	
C		62.9	66.06	69.28	
H	x	4.2	4.16	3.35	
H		5.81	6.16	6.18	
H	x	2.1	2.53	1.96	
H		2.11	1.42	2.95	
H		1.6	2.06	1.16	
H		1.6	1.78	1.56	
H		1.6	1.35	1.49	
H	x	3.87	3.82	3.48	
H		2.03	0.74	0.84	
H		1.19	0.76	1.9	
H		0.88	1.93	2.35	
H		1.19	0.91	0.87	
H		4.33	4.91	5.68	
H		3.14	3.84	3.42	
H		3.35	3.85	3.82	
H		3.34	3.93	3.86	
H		3.25	4.34	3.65	
H		3.65	4.03	3.7	
H		3.85	3.33	4.2	

Figure S56: Results of DP4+ Analysis (Isomer 1 is **2*R*,6*R*,9*R***; Isomer 2 is **2*R*,6*R*,9*S***)

ECD calculation of 3

Table.S 7 Energy of structure for ECD calculation

conformer	Energy (Hartree)	Energy (kcal/mol)	Population (%)
2	-734.5051176	-460908.92	79.82
1	-734.5036195	-460907.98	16.33
3	-734.5022537	-460907.12	3.84

Table.S 8 Standard orientations at B3LYP/6-311G(d,p) level in Methanol.

Conformer 2					
Center	Atomic	Atomic	Coordinates (Angstroms)		
Number	Number	Type	X	Y	Z
1	6	0	-0.8796	-1.563	0.3017
2	6	0	-0.7314	-0.2822	-0.5323
3	6	0	-1.5075	0.9686	0.0485
4	6	0	-2.8797	0.4887	0.5862
5	6	0	-2.6921	-0.5976	1.6404
6	6	0	-1.7335	-1.6779	1.3335
7	6	0	-1.765	1.9982	-1.0791
8	6	0	-0.7488	1.7074	1.171
9	6	0	0.7369	-0.0029	-0.9797
10	6	0	1.8233	0.061	0.1095
11	6	0	3.2404	0.0824	-0.482
12	8	0	3.513	-1.1477	-1.147
13	6	0	4.2865	0.254	0.6114
14	8	0	-3.6429	1.5707	1.1179
15	8	0	-3.3733	-0.6435	2.6636
16	6	0	-0.1125	-2.7859	-0.1442
17	1	0	-1.256	-0.5283	-1.4706
18	1	0	-3.4786	0.0504	-0.2222
19	1	0	-1.8357	-2.5795	1.9271
20	1	0	-0.8325	2.3988	-1.488
21	1	0	-2.3255	1.5466	-1.9053
22	1	0	-2.3454	2.854	-0.716
23	1	0	0.0938	2.2798	0.7717
24	1	0	-0.3743	1.0185	1.9346
25	1	0	-1.3872	2.4396	1.6783
26	1	0	0.7775	0.9221	-1.5653
27	1	0	1.0056	-0.7932	-1.6925
28	1	0	1.7027	0.9597	0.7159
29	1	0	1.7345	-0.7929	0.7872
30	1	0	3.3436	0.8988	-1.2062
31	1	0	3.134	-1.0911	-2.0401
32	1	0	4.1472	1.1947	1.1526
33	1	0	4.2455	-0.5738	1.3282
34	1	0	5.2935	0.2461	0.1803
35	1	0	-4.1951	1.1881	1.8302
36	1	0	-0.4298	-3.6864	0.3936

37	1	0	0.9618	-2.6771	0.0177
38	1	0	-0.2878	-2.9702	-1.2094

Conformer 1

Center	Atomic	Atomic	Coordinates (Angstroms)		
Number	Number	Type	X	Y	Z
1	6	0	-0.9322	-1.6034	0.3244
2	6	0	-0.743	-0.3394	-0.5293
3	6	0	-1.4564	0.9425	0.0601
4	6	0	-2.8689	0.5266	0.5472
5	6	0	-2.7747	-0.5846	1.5879
6	6	0	-1.8284	-1.6875	1.3232
7	6	0	-1.6221	2.014	-1.0442
8	6	0	-0.6865	1.6025	1.225
9	6	0	0.7261	-0.1274	-1.0046
10	6	0	1.8128	-0.0117	0.073
11	6	0	3.212	-0.3769	-0.4437
12	8	0	4.141	-0.2744	0.6343
13	6	0	3.6984	0.54	-1.5573
14	8	0	-3.5912	1.6382	1.0749
15	8	0	-3.504	-0.6197	2.5779
16	6	0	-0.1473	-2.8372	-0.0496
17	1	0	-1.2915	-0.5728	-1.4571
18	1	0	-3.4635	0.1339	-0.2873
19	1	0	-1.963	-2.5752	1.9313
20	1	0	-0.6566	2.3808	-1.406
21	1	0	-2.1719	1.6136	-1.9031
22	1	0	-2.1734	2.887	-0.6768
23	1	0	0.1802	2.1631	0.8649
24	1	0	-0.3429	0.8687	1.9605
25	1	0	-1.3065	2.3323	1.758
26	1	0	0.7786	0.7528	-1.6545
27	1	0	0.9813	-0.9702	-1.6617
28	1	0	1.8579	1.0083	0.4652
29	1	0	1.5839	-0.6438	0.9378
30	1	0	3.2278	-1.415	-0.7941
31	1	0	3.8491	-0.8797	1.3371
32	1	0	3.0785	0.4553	-2.4543
33	1	0	3.7022	1.5855	-1.2299
34	1	0	4.7307	0.2936	-1.8296
35	1	0	-4.1958	1.2721	1.7523
36	1	0	-0.4871	-3.7221	0.5002
37	1	0	0.9176	-2.7155	0.1632
38	1	0	-0.2715	-3.0544	-1.1156

Conformer 3

Center	Atomic	Atomic	Coordinates (Angstroms)		
Number	Number	Type	X	Y	Z
1	6	0	-0.8829	-1.5681	0.2857
2	6	0	-0.7401	-0.2827	-0.5415
3	6	0	-1.5143	0.9662	0.048
4	6	0	-2.8781	0.4835	0.6036

5	6	0	-2.6736	-0.6039	1.6536
6	6	0	-1.7229	-1.6859	1.3284
7	6	0	-1.7892	1.9934	-1.078
8	6	0	-0.7447	1.7105	1.1594
9	6	0	0.7256	0.0019	-0.9947
10	6	0	1.8229	0.0357	0.0864
11	6	0	3.2481	0.1554	-0.4778
12	8	0	3.3522	1.3047	-1.312
13	6	0	3.6914	-1.0601	-1.2794
14	8	0	-3.6347	1.5634	1.1491
15	8	0	-3.3361	-0.6508	2.6889
16	6	0	-0.1351	-2.793	-0.1854
17	1	0	-1.2696	-0.5241	-1.4782
18	1	0	-3.4879	0.0459	-0.197
19	1	0	-1.8242	-2.5917	1.9158
20	1	0	-0.8632	2.3973	-1.4985
21	1	0	-2.3577	1.5387	-1.8969
22	1	0	-2.3686	2.8474	-0.7091
23	1	0	0.0998	2.2733	0.7502
24	1	0	-0.3716	1.026	1.9276
25	1	0	-1.3757	2.4515	1.6632
26	1	0	0.7567	0.9382	-1.5617
27	1	0	0.9861	-0.775	-1.7243
28	1	0	1.6708	0.9021	0.7318
29	1	0	1.7731	-0.8425	0.7358
30	1	0	3.9461	0.2899	0.357
31	1	0	3.1435	2.0828	-0.7668
32	1	0	3.5786	-1.9824	-0.7021
33	1	0	3.1349	-1.1542	-2.2172
34	1	0	4.745	-0.9569	-1.5626
35	1	0	-4.1845	1.1769	1.8608
36	1	0	-0.4523	-3.6962	0.3479
37	1	0	0.9425	-2.6958	-0.0397
38	1	0	-0.3288	-2.9642	-1.2496

NMR calculation of 4

Table.S 27 Small molecule configuration search results of **4 (2*S*,6*S*,9*R*)**

Conformer	Energy (kcal/mol)	Population (%)
1	47.13	28.6
2	47.61	12.7
3	47.67	11.5
4	47.69	11.1
5	47.8	9.2
6	48.25	4.3
7	48.32	3.8
8	48.33	3.8
9	48.61	2.4
10	48.62	2.3
11	49.01	1.2
12	49.13	1
13	49.17	0.9
14	49.27	0.8
15	49.27	0.8
16	49.34	0.7
17	49.34	0.7
18	49.37	0.6
19	49.73	0.4
20	49.78	0.3
21	49.88	0.3
22	49.89	0.3
23	49.97	0.2
24	50.01	0.2
25	50.12	0.2
26	50.15	0.2
27	50.2	0.2
28	50.23	0.2
29	50.24	0.2
30	50.26	0.2
31	50.46	0.1
32	50.49	0.1
33	50.66	0.1
34	50.76	0.1
35	50.96	0
36	50.99	0
37	51.2	0
38	51.21	0
39	51.31	0
40	51.31	0
41	51.44	0
42	51.46	0

43	51.55	0
44	51.6	0
45	51.61	0
46	51.67	0
47	51.68	0
48	51.72	0
49	51.74	0
50	51.76	0
51	51.83	0
52	51.85	0
53	51.86	0
54	51.86	0
55	52.1	0
56	52.2	0
57	52.21	0
58	52.22	0
59	52.23	0
60	52.23	0
61	52.36	0
62	52.37	0
63	52.39	0
64	52.62	0
65	52.76	0
66	52.87	0
67	52.93	0
68	53.01	0
69	53.21	0
70	53.24	0
71	53.26	0
72	53.32	0
73	53.33	0
74	53.43	0
75	53.57	0
76	53.58	0
77	53.63	0
78	53.65	0
79	53.9	0
80	53.97	0
81	54	0
82	54.06	0

Table.S 98 Geometric optimization at PM6 theoretical level of **4 (2*S*,6*S*,9*R*)**

Conformer	Energy (Hartree)	Energy (kcal/mol)	Population (%)
11	-0.2156985	-135.35	28.08
5	-0.2156985	-135.35	28.08

10	-0.2150116	-134.92	13.57
2	-0.2150116	-134.92	13.57
6	-0.2150116	-134.92	13.57
1	-0.2121066	-133.1	0.63
4	-0.2121066	-133.1	0.63
7	-0.2121066	-133.1	0.63
9	-0.2121066	-133.1	0.63
3	-0.2114691	-132.7	0.32
8	-0.2114691	-132.7	0.32

Table.S 29 Geometric optimization at HF/6-31G(d) theoretical level of **4 (2*S*,6*S*,9*R*)**

Conformer	Energy (Hartree)	Energy (kcal/mol)	Population (%)
1	-728.4834583	-457130.27	66.53
11	-728.4827033	-457129.79	29.9
10	-728.4806952	-457128.53	3.57

Table.S 30 Geometric optimization at B3LYP/6-31G(d) theoretical level of **4 (2*S*,6*S*,9*R*)**

Conformer	Energy (Hartree)	Energy (kcal/mol)	Population (%)
11	-733.1135593	-460035.7	49.58
1	-733.1134473	-460035.63	44.04
10	-733.1116234	-460034.49	6.38

Table.S 31 Energy of structure for NMR calculation

	conformer	Energy (Hartree)	Energy (kcal/mol)	Population (%)
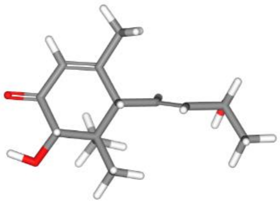	11	-733.1135593	-460035.7	49.58
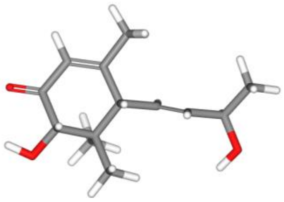	1	-733.1134473	-460035.63	44.04

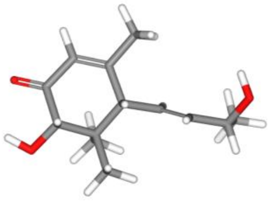

10	-733.1116234	-460034.49	6.38	

Table.S 32 Standard orientations at mPW1PW91/6-311G(2d,p) level in Methanol. of **4 (2*S*,6*S*,9*R*)**

Conformer 11					
Center Number	Atomic Number	Atomic Type	Coordinates (Angstroms) X	Y	Z
1	6	0	0.7925	1.6477	-0.2407
2	6	0	0.0726	0.3175	-0.4788
3	6	0	0.8865	-0.9265	0.0009
4	6	0	2.3303	-0.7545	-0.5073
5	6	0	2.9512	0.524	0.02
6	6	0	2.1021	1.7198	-0.0165
7	6	0	0.8854	-1.0723	1.5325
8	6	0	0.3062	-2.204	-0.6214
9	8	0	3.1218	-1.8486	-0.1821
10	8	0	4.0906	0.5333	0.395
11	6	0	-1.3289	0.3496	0.0932
12	6	0	-2.43	0.1752	-0.6122
13	6	0	-3.8371	0.2054	-0.0599
14	8	0	-3.867	0.2983	1.3396
15	6	0	-4.6561	-0.9877	-0.5499
16	6	0	-0.0351	2.9039	-0.33
17	1	0	-0.0255	0.2337	-1.5613
18	1	0	2.291	-0.6411	-1.5935
19	1	0	2.598	2.6598	0.1463
20	1	0	1.177	-0.1572	2.0376
21	1	0	1.5771	-1.8518	1.8241
22	1	0	-0.098	-1.3507	1.8946
23	1	0	-0.7179	-2.3622	-0.3044
24	1	0	0.8932	-3.0635	-0.3241
25	1	0	0.3164	-2.153	-1.7069
26	1	0	3.971	-1.5169	0.0927
27	1	0	-1.4155	0.5451	1.148
28	1	0	-2.3603	0.0008	-1.6756
29	1	0	-4.3173	1.1143	-0.4073
30	1	0	-3.5416	-0.5109	1.7124
31	1	0	-4.2126	-1.9224	-0.2152
32	1	0	-4.7045	-1.0133	-1.6338
33	1	0	-5.6662	-0.9279	-0.1614
34	1	0	0.5931	3.785	-0.3002
35	1	0	-0.7511	2.9635	0.4823
36	1	0	-0.6066	2.9161	-1.2534
Conformer 1					

Center Number	Atomic Number	Atomic Type	Coordinates (Angstroms) X	Y	Z
1	6	0	0.6944	1.6287	-0.114
2	6	0	0.0719	0.2512	-0.3572
3	6	0	1.0147	-0.9359	0.0199
4	6	0	2.4022	-0.6258	-0.5733
5	6	0	2.9503	0.6814	-0.0357
6	6	0	2.0057	1.8027	0.0282
7	6	0	0.4997	-2.2355	-0.6141
8	6	0	1.1295	-1.1341	1.5415
9	8	0	3.2998	-1.6602	-0.3433
10	8	0	4.1068	0.7742	0.2679
11	6	0	-1.2872	0.1414	0.301
12	6	0	-2.4201	-0.0858	-0.3374
13	6	0	-3.7712	-0.1916	0.3225
14	8	0	-4.3733	-1.4284	0.0185
15	6	0	-4.7314	0.8839	-0.1635
16	6	0	-0.2362	2.814	-0.1025
17	1	0	-0.0934	0.1965	-1.4332
18	1	0	2.2805	-0.4787	-1.6492
19	1	0	2.4335	2.7751	0.194
20	1	0	0.4287	-2.146	-1.6947
21	1	0	1.1749	-3.0517	-0.3919
22	1	0	-0.4828	-2.4919	-0.2354
23	1	0	1.3785	-0.2154	2.0629
24	1	0	1.902	-1.8609	1.7556
25	1	0	0.2001	-1.5086	1.9555
26	1	0	4.1365	-1.2691	-0.1119
27	1	0	-1.3102	0.2669	1.3723
28	1	0	-2.4231	-0.2187	-1.4092
29	1	0	-3.6476	-0.0955	1.3997
30	1	0	-3.8112	-2.1325	0.3121
31	1	0	-4.864	0.8123	-1.2379
32	1	0	-5.6995	0.7536	0.3062
33	1	0	-4.3546	1.8724	0.0762
34	1	0	0.3193	3.7429	-0.0879
35	1	0	-0.8744	2.8046	-0.981
36	1	0	-0.8913	2.7927	0.7618

Conformer 10

Center Number	Atomic Number	Atomic Type	Coordinates (Angstroms) X	Y	Z
1	6	0	0.5776	1.5911	-0.1474
2	6	0	0.0523	0.1854	-0.45
3	6	0	1.0413	-0.9526	-0.04
4	6	0	2.4399	-0.5493	-0.5449
5	6	0	2.8774	0.7758	0.047
6	6	0	1.8657	1.8387	0.0745
7	6	0	1.0797	-1.1763	1.4818
8	6	0	0.6414	-2.2661	-0.7262
9	8	0	3.383	-1.534	-0.2811

10	8	0	4.0066	0.9301	0.4203
11	6	0	-1.3324	-0.0183	0.1294
12	6	0	-2.4001	-0.3246	-0.5844
13	6	0	-3.8066	-0.5552	-0.0809
14	8	0	-4.7116	0.2037	-0.8525
15	6	0	-4.0221	-0.3097	1.4071
16	6	0	-0.42	2.7205	-0.1711
17	1	0	-0.0486	0.1431	-1.5344
18	1	0	2.372	-0.3867	-1.6233
19	1	0	2.2249	2.8309	0.2812
20	1	0	0.152	-1.6138	1.833
21	1	0	1.243	-0.256	2.0337
22	1	0	1.8807	-1.861	1.7279
23	1	0	1.3508	-3.0456	-0.4799
24	1	0	0.6278	-2.1575	-1.8073
25	1	0	-0.3439	-2.5878	-0.4101
26	1	0	4.1802	-1.0996	0.0057
27	1	0	-1.4171	0.0943	1.1969
28	1	0	-2.3083	-0.4323	-1.6544
29	1	0	-4.071	-1.5837	-0.2998
30	1	0	-4.5249	1.1255	-0.7312
31	1	0	-5.0641	-0.4831	1.6476
32	1	0	-3.779	0.7146	1.6772
33	1	0	-3.4158	-0.9734	2.0139
34	1	0	0.0779	3.6797	-0.1094
35	1	0	-1.1204	2.6451	0.654
36	1	0	-1.0046	2.6914	-1.0858

Table.S 33 Small molecule configuration search results of **4 (2*S*,6*R*,9*S*)**

Conformer	Energy (kcal/mol)	Population (%)
1	45.52	23.5
2	45.7	17.3
3	45.96	11.2
4	46.18	7.7
5	46.25	6.8
6	46.35	5.8
7	46.47	4.7
8	46.48	4.6
9	46.8	2.7
10	46.83	2.6
11	46.87	2.4
12	46.9	2.3
13	47.07	1.7
14	47.14	1.5
15	47.17	1.5
16	47.63	0.7
17	47.68	0.6
18	47.94	0.4

19	47.95	0.4
20	48.17	0.3
21	48.26	0.2
22	48.31	0.2
23	48.35	0.2
24	48.42	0.2
25	48.5	0.2
26	48.96	0.1
27	49	0.1
28	49.17	0
29	49.58	0
30	49.66	0
31	49.69	0
32	49.71	0
33	49.73	0
34	49.79	0
35	49.89	0
36	50.04	0
37	50.05	0
38	50.15	0
39	50.22	0
40	50.31	0
41	50.34	0
42	50.48	0
43	50.53	0
44	50.62	0
45	50.65	0
46	50.65	0
47	50.69	0
48	50.88	0
49	51.07	0
50	51.28	0
51	51.41	0
52	51.41	0
53	51.42	0
54	51.62	0
55	51.89	0
56	52.04	0
57	52.09	0
58	52.12	0
59	52.18	0
60	52.37	0
61	52.44	0

Table.S 34 Geometric optimization at PM6 theoretical level of **4 (2*S*,6*R*,9*S*)**

Conformer	Energy (Hartree)	Energy (kcal/mol)	Population (%)

11	-0.2159525	-135.51	17.6
8	-0.2159525	-135.51	17.6
12	-0.2156838	-135.34	13.24
13	-0.2156838	-135.34	13.24
3	-0.2156838	-135.34	13.24
4	-0.2156838	-135.34	13.24
9	-0.2152563	-135.08	8.42
1	-0.212847	-133.56	0.66
10	-0.212847	-133.56	0.66
15	-0.212847	-133.56	0.66
7	-0.212847	-133.56	0.66
2	-0.2119929	-133.03	0.27
6	-0.2116243	-132.8	0.18
14	-0.2115824	-132.77	0.17
5	-0.2115824	-132.77	0.17

Table.S 35 Geometric optimization at HF/6-31G(d) theoretical level of **4 (2*S*,6*R*,9*S*)**

Conformer	Energy (Hartree)	Energy (kcal/mol)	Population (%)
1	-728.4844395	-457130.88	72.61
11	-728.4833464	-457130.2	22.82
12	-728.4815898	-457129.1	3.55
9	-728.4804104	-457128.36	1.02

Table.S 36 Geometric optimization at B3LYP/6-31G(d) theoretical level of **4 (2*S*,6*R*,9*S*)**

Conformer	Energy (Hartree)	Energy (kcal/mol)	Population (%)
11	-733.1139821	-460035.97	47.76
1	-733.1138662	-460035.89	42.24
12	-733.1121514	-460034.82	6.87
9	-733.1114094	-460034.35	3.13

Table.S 37 Energy of structure for NMR calculation

	Conformer	Energy (Hartree)	Energy (kcal/mol)	Population (%)
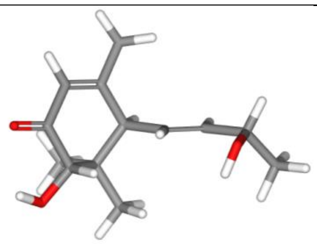	11	-733.1139821	-460035.97	47.76

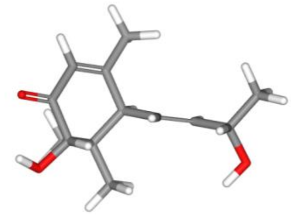	1	-733.1138662	-460035.89	42.24
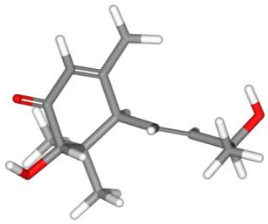	12	-733.1121514	-460034.82	6.87
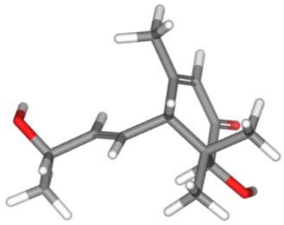	9	-733.1114094	-460034.35	3.13

Table.S 38 Standard orientations at mPW1PW91/6-311G(2d,p) level in Methanol. of **4 (2*S*,6*R*,9*S*)**

Conformer 11					
Center Number	Atomic Number	Atomic Type	Coordinates (Angstroms) X	Y	Z
1	6	0	0.7877	1.685	0.3047
2	6	0	0.0741	0.4103	0.7464
3	6	0	1.0051	-0.8415	0.7219
4	6	0	1.7369	-0.8794	-0.6305
5	6	0	2.5025	0.4074	-0.8901
6	6	0	1.8689	1.6645	-0.4698
7	8	0	2.59	-1.9738	-0.7132
8	6	0	2.0182	-0.7504	1.8774
9	6	0	0.1986	-2.1348	0.9009
10	6	0	-1.1907	0.27	-0.0811
11	6	0	-2.4139	0.3247	0.4111
12	6	0	-3.6874	0.2043	-0.3952
13	8	0	-3.4487	-0.1327	-1.7357
14	6	0	-4.6667	-0.7738	0.2521
15	8	0	3.5707	0.3662	-1.4345
16	6	0	0.1917	2.9801	0.7859
17	1	0	-0.2339	0.5545	1.7788
18	1	0	0.9896	-0.9429	-1.4235
19	1	0	2.3624	2.5678	-0.7807
20	1	0	3.3675	-1.6964	-1.1873
21	1	0	1.5019	-0.8033	2.8314
22	1	0	2.7166	-1.5756	1.8277
23	1	0	2.5875	0.1723	1.8627
24	1	0	-0.473	-2.3146	0.0694

25	1	0	-0.3997	-2.0912	1.8065
26	1	0	0.8691	-2.9815	0.9756
27	1	0	-1.0652	0.1529	-1.1439
28	1	0	-2.5561	0.469	1.4718
29	1	0	-4.1578	1.1812	-0.4357
30	1	0	-3.1223	-1.0222	-1.7801
31	1	0	-4.2356	-1.7705	0.3062
32	1	0	-4.9199	-0.4667	1.2618
33	1	0	-5.5789	-0.8246	-0.3311
34	1	0	-0.8551	3.0471	0.5102
35	1	0	0.2437	3.0331	1.8705
36	1	0	0.7139	3.8354	0.3765

Conformer 1

Center Number	Atomic Number	Atomic Type	Coordinates (Angstroms) X	Y	Z
1	6	0	0.6497	1.6292	0.3712
2	6	0	0.0755	0.2529	0.6953
3	6	0	1.1449	-0.8807	0.6232
4	6	0	1.9204	-0.7385	-0.6976
5	6	0	2.536	0.6434	-0.8426
6	6	0	1.7478	1.787	-0.3625
7	8	0	2.8992	-1.718	-0.816
8	6	0	0.4866	-2.2658	0.6807
9	6	0	2.1021	-0.7589	1.8227
10	6	0	-1.1375	0.0278	-0.1889
11	6	0	-2.3795	-0.0733	0.2475
12	6	0	-3.5852	-0.2776	-0.6338
13	8	0	-4.2814	-1.4411	-0.2512
14	6	0	-4.5768	0.8713	-0.5257
15	8	0	3.6161	0.7648	-1.3498
16	6	0	-0.1025	2.8096	0.9234
17	1	0	-0.2831	0.2859	1.7206
18	1	0	1.2129	-0.8337	-1.5234
19	1	0	2.1437	2.7605	-0.5906
20	1	0	3.6524	-1.3222	-1.243
21	1	0	1.2469	-3.0351	0.7246
22	1	0	-0.132	-2.4591	-0.1883
23	1	0	-0.1414	-2.3552	1.5626
24	1	0	2.563	0.2202	1.8922
25	1	0	1.5639	-0.9358	2.7491
26	1	0	2.8911	-1.4956	1.747
27	1	0	-0.9533	-0.0251	-1.2509
28	1	0	-2.5899	-0.0236	1.3056
29	1	0	-3.2572	-0.366	-1.6679
30	1	0	-3.7012	-2.1881	-0.3105
31	1	0	-4.1238	1.8015	-0.8517
32	1	0	-5.4447	0.6686	-1.1426
33	1	0	-4.9101	0.9859	0.5003
34	1	0	0.3213	3.7463	0.5847
35	1	0	-0.0729	2.7928	2.0099

36	1	0	-1.1462	2.7734	0.6306

Conformer 12

Center	Atomic	Atomic	Coordinates (Angstroms)		
Number	Number	Type	X	Y	Z
1	6	0	0.5259	1.6327	0.2975
2	6	0	0.0558	0.2624	0.7766
3	6	0	1.1862	-0.8123	0.7341
4	6	0	1.8687	-0.7499	-0.6431
5	6	0	2.3871	0.6449	-0.9525
6	6	0	1.5631	1.7829	-0.5214
7	8	0	2.8978	-1.6792	-0.7391
8	6	0	0.6178	-2.2201	0.9582
9	6	0	2.2064	-0.5274	1.8511
10	6	0	-1.1912	-0.1089	-0.0066
11	6	0	-2.3891	-0.2558	0.5297
12	6	0	-3.6749	-0.6132	-0.1802
13	8	0	-4.6886	0.2917	0.1996
14	6	0	-3.5819	-0.7211	-1.6968
15	8	0	3.4227	0.7792	-1.5425
16	6	0	-0.2569	2.816	0.7985
17	1	0	-0.2423	0.3677	1.8163
18	1	0	1.119	-0.9618	-1.4077
19	1	0	1.8849	2.7518	-0.8594
20	1	0	3.596	-1.2838	-1.2513
21	1	0	0.0554	-2.2626	1.8866
22	1	0	1.4248	-2.9395	1.0157
23	1	0	-0.0445	-2.5254	0.1565
24	1	0	3.0316	-1.2247	1.7896
25	1	0	2.6119	0.4771	1.8015
26	1	0	1.7376	-0.6456	2.8236
27	1	0	-1.061	-0.2389	-1.0677
28	1	0	-2.5143	-0.1157	1.5925
29	1	0	-4.0122	-1.5666	0.2108
30	1	0	-4.4568	1.1605	-0.1019
31	1	0	-3.2525	0.2152	-2.1394
32	1	0	-4.561	-0.957	-2.0959
33	1	0	-2.892	-1.501	-2.0004
34	1	0	-1.3149	2.6929	0.594
35	1	0	0.0803	3.7393	0.3449
36	1	0	-0.1473	2.9033	1.8764

Conformer 9

Center	Atomic	Atomic	Coordinates (Angstroms)		
Number	Number	Type	X	Y	Z
1	6	0	-0.4418	1.6986	-0.1584
2	6	0	-0.2702	0.5158	-1.0999
3	6	0	-1.3893	-0.5615	-0.9304
4	6	0	-1.512	-0.8835	0.5678
5	6	0	-1.794	0.3646	1.3874
6	6	0	-1.114	1.6045	0.9854
7	8	0	-2.4904	-1.8443	0.8008

8	6	0	-1.0521	-1.8478	-1.6981
9	6	0	-2.7203	-0.0189	-1.4802
10	6	0	1.1428	-0.0488	-1.0243
11	6	0	1.9942	0.047	-0.0204
12	6	0	3.3828	-0.5425	-0.0239
13	8	0	4.3564	0.4642	0.1378
14	6	0	3.5435	-1.6277	1.0374
15	8	0	-2.5347	0.3068	2.3294
16	6	0	0.18	2.9956	-0.6026
17	1	0	-0.3737	0.9068	-2.1105
18	1	0	-0.5457	-1.2537	0.9117
19	1	0	-1.2415	2.4469	1.6418
20	1	0	-2.9301	-1.6156	1.6134
21	1	0	-0.8662	-1.6336	-2.7475
22	1	0	-1.8822	-2.5397	-1.6383
23	1	0	-0.1782	-2.3464	-1.2948
24	1	0	-2.6484	0.1311	-2.5535
25	1	0	-3.5166	-0.7277	-1.2934
26	1	0	-3.0033	0.9281	-1.0345
27	1	0	1.4588	-0.5755	-1.9099
28	1	0	1.7171	0.5743	0.8806
29	1	0	3.5829	-0.9682	-0.9987
30	1	0	4.3156	0.8072	1.0208
31	1	0	3.3453	-1.2306	2.0309
32	1	0	4.5566	-2.0141	1.0236
33	1	0	2.8539	-2.4469	0.8647
34	1	0	1.2265	2.8575	-0.851
35	1	0	-0.3194	3.3555	-1.4984
36	1	0	0.1002	3.7589	0.1612

Table.S 39 Small molecule configuration search results of **4 (2*R*,6*S*,9*S*)**

Conformer	Energy (kcal/mol)	Population (%)
1	45.65	22
2	45.85	15.7
3	45.99	12.4
4	46.16	9.3
5	46.24	8.1
6	46.47	5.5
7	46.48	5.4
8	46.72	3.6
9	46.73	3.6
10	47.1	1.9
11	47.17	1.7
12	47.18	1.7
13	47.32	1.3
14	47.36	1.2
15	47.37	1.2
16	47.45	1.1
17	47.49	1

18	47.64	0.8
19	48.22	0.3
20	48.22	0.3
21	48.28	0.3
22	48.33	0.2
23	48.36	0.2
24	48.45	0.2
25	48.59	0.2
26	48.66	0.1
27	48.69	0.1
28	48.92	0.1
29	48.96	0.1
30	49.14	0.1
31	49.23	0
32	49.3	0
33	49.36	0
34	49.49	0
35	49.57	0
36	49.66	0
37	49.88	0
38	49.89	0
39	50	0
40	50.44	0
41	50.46	0
42	50.67	0
43	50.7	0
44	50.72	0
45	50.84	0
46	50.91	0
47	50.99	0
48	51.33	0
49	51.41	0
50	51.47	0
51	51.52	0
52	51.62	0
53	51.69	0
54	51.83	0
55	51.94	0
56	52.28	0
57	52.45	0
58	52.52	0
59	52.63	0

Table.S 40 Geometric optimization at PM6 theoretical level of **4 (2*R*,6*S*,9*S*)**

Conformer	Energy (Hartree)	Energy (kcal/mol)	Population (%)
4	-0.2161616	-135.64	20.24
10	-0.2161615	-135.64	20.24
13	-0.2157751	-135.4	13.44

7	-0.2157751	-135.4	13.44
14	-0.2155748	-135.28	10.87
16	-0.2155748	-135.28	10.87
12	-0.2145539	-134.63	3.69
15	-0.2145538	-134.63	3.69
11	-0.2135184	-133.98	1.23
2	-0.2126161	-133.42	0.47
3	-0.2126161	-133.42	0.47
8	-0.212616	-133.42	0.47
9	-0.2121428	-133.12	0.29
1	-0.2121427	-133.12	0.29
5	-0.2116647	-132.82	0.17
6	-0.2114086	-132.66	0.13

Table.S 41 Geometric optimization at HF/6-31G(d) theoretical level of **4 (2*R*,6*S*,9*S*)**

Conformer	Energy (Hartree)	Energy (kcal/mol)	Population (%)
13	-728.483127	-457130.06	43.62
11	-728.482838	-457129.88	32.12
14	-728.48195	-457129.32	12.54
4	-728.4817567	-457129.2	10.22
12	-728.4799424	-457128.06	1.5

Table.S 42 Geometric optimization at B3LYP/6-31G(d) theoretical level of **4 (2*R*,6*S*,9*S*)**

Conformer	Energy (Hartree)	Energy (kcal/mol)	Population (%)
13	-733.1139251	-460035.93	54.94
14	-733.1127612	-460035.2	16.01
4	-733.1126943	-460035.16	14.92
11	-733.1123842	-460034.96	10.74
12	-733.111293	-460034.28	3.38

Table.S 43 Energy of structure for NMR calculation

Compound	conformer	Energy (Hartree)	Energy (kcal/mol)	Population (%)
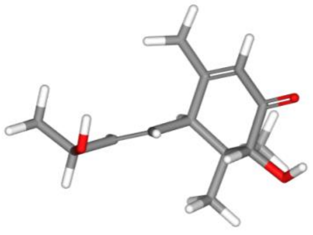	13	-733.1139251	-460035.93	54.94

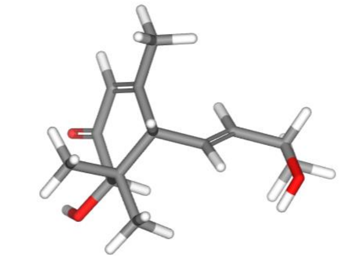	14	-733.1127612	-460035.2	16.01
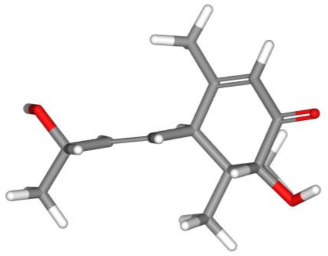	4	-733.1126943	-460035.16	14.92
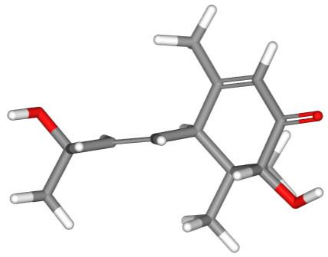	11	-733.1123842	-460034.96	10.74
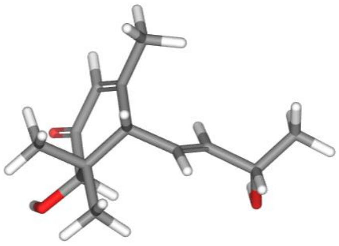	12	-733.111293	-460034.28	3.38

Table.S 10 Standard orientations at mPW1PW91/6-311G(2d,p) level in Methanol. of **4 (2*R*,6*S*,9*S*)**

Conformer 13					
Center Number	Atomic Number	Atomic Type	Coordinates (Angstroms) X	Y	Z
1	6	0	-0.5084	1.6118	0.363
2	6	0	-0.0522	0.2173	0.7818
3	6	0	-1.1966	-0.8401	0.7047
4	6	0	-1.8876	-0.7141	-0.6641
5	6	0	-2.3886	0.6987	-0.915
6	6	0	-1.5481	1.8082	-0.4428
7	8	0	-2.9316	-1.6238	-0.7868
8	6	0	-2.2056	-0.5877	1.8395
9	6	0	-0.6435	-2.2624	0.8672
10	6	0	1.1814	-0.1385	-0.028
11	6	0	2.3857	-0.3131	0.4824
12	6	0	3.6278	-0.6587	-0.3078

13	8	0	3.4185	-0.6082	-1.6939
14	6	0	4.8127	0.2151	0.1011
15	8	0	-3.4251	0.8709	-1.4939
16	6	0	0.2922	2.764	0.9068
17	1	0	0.2543	0.2768	1.8229
18	1	0	-1.1467	-0.9071	-1.4417
19	1	0	-1.8595	2.7938	-0.7395
20	1	0	-3.6238	-1.2019	-1.2855
21	1	0	-2.6004	0.4223	1.832
22	1	0	-1.7314	-0.7487	2.8031
23	1	0	-3.039	-1.273	1.7571
24	1	0	-1.4585	-2.9744	0.8981
25	1	0	-0.0792	-2.3495	1.7915
26	1	0	0.0131	-2.5405	0.0513
27	1	0	1.0497	-0.2453	-1.091
28	1	0	2.5312	-0.226	1.5488
29	1	0	3.8808	-1.6938	-0.1041
30	1	0	3.2703	0.2917	-1.9543
31	1	0	4.6036	1.2641	-0.0962
32	1	0	5.6946	-0.0702	-0.4607
33	1	0	5.0303	0.1133	1.1595
34	1	0	1.3475	2.6355	0.6918
35	1	0	-0.0353	3.7092	0.4926
36	1	0	0.1885	2.8094	1.988

Conformer 14

Center Number	Atomic Number	Atomic Type	Coordinates (Angstroms) X	Y	Z
1	6	0	0.634	1.698	-0.3653
2	6	0	0.2309	0.4387	-1.1176
3	6	0	1.2045	-0.7561	-0.8593
4	6	0	1.3929	-0.8916	0.6603
5	6	0	1.9013	0.3998	1.279
6	6	0	1.369	1.6607	0.7426
7	8	0	2.2463	-1.9446	0.9732
8	6	0	2.5523	-0.488	-1.5524
9	6	0	0.6427	-2.0712	-1.4184
10	6	0	-1.2356	0.0959	-0.8859
11	6	0	-1.9875	0.4538	0.1387
12	6	0	-3.4436	0.0828	0.3137
13	8	0	-3.8938	-0.8283	-0.6534
14	6	0	-3.7203	-0.4329	1.7252
15	8	0	2.6946	0.3602	2.1782
16	6	0	0.17	3.0017	-0.958
17	1	0	0.3107	0.6724	-2.1778
18	1	0	0.4135	-1.0688	1.1053
19	1	0	1.6589	2.5554	1.2644
20	1	0	2.7715	-1.6764	1.7204
21	1	0	3.2579	-1.2722	-1.3105
22	1	0	2.992	0.4595	-1.2618
23	1	0	2.422	-0.4733	-2.6305

24	1	0	1.376	-2.8598	-1.3105
25	1	0	-0.2554	-2.3842	-0.8983
26	1	0	0.4053	-1.9721	-2.4744
27	1	0	-1.6886	-0.4913	-1.6646
28	1	0	-1.5772	1.0629	0.9284
29	1	0	-4.0416	0.9737	0.153
30	1	0	-3.4775	-1.668	-0.5079
31	1	0	-3.145	-1.3336	1.9256
32	1	0	-4.7732	-0.6663	1.8342
33	1	0	-3.4515	0.3055	2.4737
34	1	0	0.6624	3.1669	-1.913
35	1	0	0.3959	3.8389	-0.3096
36	1	0	-0.8976	2.983	-1.1463

Conformer 4

Center Number	Atomic Number	Atomic Type	Coordinates (Angstroms) X	Y	Z
1	6	0	-0.6973	1.6465	0.3285
2	6	0	-0.0815	0.2959	0.6835
3	6	0	-1.1153	-0.8716	0.6431
4	6	0	-1.9022	-0.7824	-0.6755
5	6	0	-2.5628	0.5758	-0.8445
6	6	0	-1.8061	1.7535	-0.3983
7	8	0	-2.8499	-1.795	-0.7674
8	6	0	-0.4136	-2.2338	0.7268
9	6	0	-2.0695	-0.7527	1.8453
10	6	0	1.1358	0.0923	-0.1995
11	6	0	2.3787	0.0234	0.2368
12	6	0	3.5916	-0.1571	-0.641
13	8	0	4.4757	0.9329	-0.507
14	6	0	4.3081	-1.475	-0.3596
15	8	0	-3.6509	0.6515	-1.3437
16	6	0	0.0307	2.8616	0.8361
17	1	0	0.2783	0.3645	1.7068
18	1	0	-1.1963	-0.8723	-1.5032
19	1	0	-2.2312	2.7091	-0.6486
20	1	0	-3.6182	-1.4311	-1.1958
21	1	0	-1.1493	-3.0254	0.7893
22	1	0	0.2096	-2.4252	-0.139
23	1	0	0.219	-2.285	1.6085
24	1	0	-1.5213	-0.893	2.7723
25	1	0	-2.5595	0.2132	1.8963
26	1	0	-2.836	-1.5146	1.7903
27	1	0	0.9537	0.0321	-1.2612
28	1	0	2.577	0.0944	1.2978
29	1	0	3.2811	-0.137	-1.6776
30	1	0	4.8802	0.911	0.3501
31	1	0	4.632	-1.5259	0.678
32	1	0	5.1842	-1.5644	-0.9923
33	1	0	3.6545	-2.3207	-0.5439
34	1	0	1.0669	2.851	0.5158

35	1	0	0.0287	2.8687	1.9233
36	1	0	-0.4314	3.7766	0.4879

Conformer 11

Center	Atomic	Atomic	Coordinates (Angstroms)		
Number	Number	Type	X	Y	Z
1	6	0	-0.6958	1.6476	0.3237
2	6	0	-0.0806	0.2979	0.6824
3	6	0	-1.1148	-0.8689	0.6455
4	6	0	-1.9002	-0.7841	-0.6743
5	6	0	-2.5588	0.5743	-0.8504
6	6	0	-1.8028	1.7529	-0.4065
7	8	0	-2.8494	-1.7957	-0.7642
8	6	0	-0.4133	-2.2309	0.7349
9	6	0	-2.0703	-0.7457	1.8461
10	6	0	1.1364	0.0916	-0.2004
11	6	0	2.379	0.0445	0.2385
12	6	0	3.5884	-0.15	-0.6326
13	8	0	4.4586	0.9227	-0.3508
14	6	0	4.2714	-1.4882	-0.3584
15	8	0	-3.6457	0.6485	-1.3532
16	6	0	0.0296	2.8633	0.8334
17	1	0	0.2802	0.3695	1.7051
18	1	0	-1.1934	-0.8783	-1.5008
19	1	0	-2.2275	2.7079	-0.6597
20	1	0	-3.6175	-1.431	-1.1923
21	1	0	0.2107	-2.4254	-0.1298
22	1	0	-1.149	-3.0225	0.7996
23	1	0	0.2188	-2.2786	1.617
24	1	0	-2.8366	-1.508	1.7935
25	1	0	-1.5229	-0.8819	2.7741
26	1	0	-2.5607	0.2203	1.8927
27	1	0	0.9529	0.0131	-1.2606
28	1	0	2.5903	0.1419	1.2922
29	1	0	3.2827	-0.113	-1.676
30	1	0	5.2572	0.8228	-0.8511
31	1	0	5.1598	-1.5995	-0.9757
32	1	0	3.6057	-2.3157	-0.5783
33	1	0	4.574	-1.5521	0.6816
34	1	0	-0.4246	3.7778	0.4735
35	1	0	1.0704	2.8467	0.5294
36	1	0	0.0119	2.8765	1.9203

Conformer 12

Center	Atomic	Atomic	Coordinates (Angstroms)		
Number	Number	Type	X	Y	Z
1	6	0	0.4034	1.6804	-0.2067
2	6	0	0.2536	0.4637	-1.1074
3	6	0	1.4066	-0.5745	-0.9186
4	6	0	1.5551	-0.8483	0.587
5	6	0	1.8022	0.4312	1.3688
6	6	0	1.0858	1.6398	0.9339

7	8	0	2.5714	-1.7657	0.8334
8	6	0	2.7141	-0.0085	-1.5002
9	6	0	1.0997	-1.8933	-1.6431
10	6	0	-1.138	-0.145	-0.9911
11	6	0	-1.9822	-0.0378	0.0178
12	6	0	-3.3496	-0.6749	0.0529
13	8	0	-3.452	-1.5924	1.1172
14	6	0	-4.4616	0.3683	0.1288
15	8	0	2.5464	0.4243	2.3096
16	6	0	-0.251	2.9479	-0.6879
17	1	0	0.332	0.8235	-2.1319
18	1	0	0.6067	-1.2434	0.9519
19	1	0	1.1964	2.5054	1.5626
20	1	0	2.9983	-1.5117	1.6451
21	1	0	3.5331	-0.688	-1.3038
22	1	0	2.625	0.1089	-2.5764
23	1	0	2.9745	0.9585	-1.0846
24	1	0	1.9524	-2.5564	-1.5742
25	1	0	0.8946	-1.7165	-2.696
26	1	0	0.2478	-2.4065	-1.2127
27	1	0	-1.4403	-0.7274	-1.846
28	1	0	-1.7149	0.5358	0.8932
29	1	0	-3.4839	-1.2709	-0.8404
30	1	0	-3.4089	-1.1298	1.9437
31	1	0	-4.4413	1.0233	-0.7359
32	1	0	-5.4279	-0.1213	0.1743
33	1	0	-4.3528	0.9866	1.0175
34	1	0	0.2395	3.2946	-1.5939
35	1	0	-1.2934	2.7761	-0.9326
36	1	0	-0.192	3.735	0.0533

Table.S 45 Small molecule configuration search results of **4 (2*R*,6*R*,9*R*)**

Conformer	Energy (kcal/mol)	Population (%)
1	47.26	22.1
2	47.27	21.8
3	47.42	16.9
4	47.71	10.4
5	48.14	5
6	48.36	3.5
7	48.39	3.3
8	48.58	2.4
9	48.58	2.4
10	48.63	2.2
11	48.95	1.3
12	49.07	1
13	49.19	0.8
14	49.29	0.7
15	49.33	0.7
16	49.37	0.6

17	49.42	0.6
18	49.52	0.5
19	49.63	0.4
20	49.67	0.4
21	49.69	0.4
22	49.7	0.4
23	49.86	0.3
24	49.87	0.3
25	49.98	0.2
26	50.05	0.2
27	50.22	0.2
28	50.35	0.1
29	50.37	0.1
30	50.4	0.1
31	50.52	0.1
32	50.53	0.1
33	50.57	0.1
34	50.58	0.1
35	50.69	0.1
36	50.71	0.1
37	50.87	0
38	51.02	0
39	51.03	0
40	51.1	0
41	51.13	0
42	51.19	0
43	51.25	0
44	51.3	0
45	51.35	0
46	51.43	0
47	51.47	0
48	51.52	0
49	51.54	0
50	51.64	0
51	51.69	0
52	51.73	0
53	51.74	0
54	51.85	0
55	51.99	0
56	52.1	0
57	52.4	0
58	52.45	0
59	52.5	0
60	52.57	0
61	52.6	0
62	52.62	0
63	52.66	0
64	52.7	0
65	52.8	0
66	52.84	0

67	52.86	0
68	52.96	0
69	53.03	0
70	53.07	0
71	53.16	0
72	53.32	0
73	53.49	0
74	53.65	0
75	53.68	0
76	53.69	0
77	53.69	0
78	53.71	0
79	53.74	0
80	53.82	0
81	53.85	0
82	53.87	0
83	54.09	0
84	54.16	0
85	54.17	0

Table.S 46 Geometric optimization at PM6 theoretical level of 4 (2R,6R,9R)

Conformer	Energy (Hartree)	Energy (kcal/mol)	Population (%)
3	-0.2154137	-135.17	25.98
7	-0.2154137	-135.17	25.98
5	-0.2152439	-135.07	21.7
9	-0.2152439	-135.07	21.7
1	-0.2119891	-133.03	0.69
10	-0.2119891	-133.03	0.69
4	-0.2119891	-133.03	0.69
6	-0.2119891	-133.03	0.69
8	-0.2119891	-133.03	0.69
11	-0.2118389	-132.93	0.59
2	-0.2118389	-132.93	0.59

Table.S 47 Geometric optimization at HF/6-31G(d) theoretical level of **4 (2*R*,6*R*,9*R*)**

Conformer	Energy (Hartree)	Energy (kcal/mol)	Population (%)
1	-728.4833386	-457130.19	44.85
11	-728.4832057	-457130.11	38.96
5	-728.4821376	-457129.44	12.57
3	-728.4809632	-457128.7	3.62

Table.S 48 Geometric optimization at B3LYP/6-31G(d) theoretical level of **4 (2*R*,6*R*,9*R*)**

Conformer	Energy (Hartree)	Energy (kcal/mol)	Population (%)
11	-733.1136956	-460035.79	37.08
5	-733.1134532	-460035.63	28.69
1	-733.1133991	-460035.6	27.09

3	-733.1121396	-460034.81	7.14

Table.S 49 Energy of structure for NMR calculation

	conformer	Energy (Hartree)	Energy (kcal/mol)	Population (%)
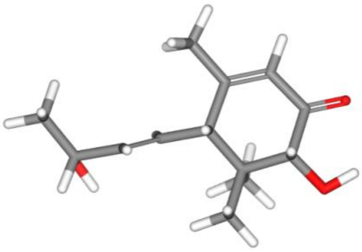	11	-733.1136956	-460035.79	37.08
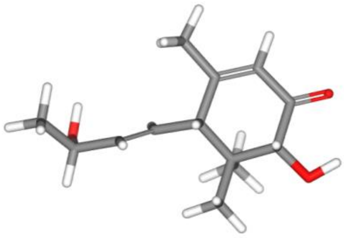	5	-733.1134532	-460035.63	28.69
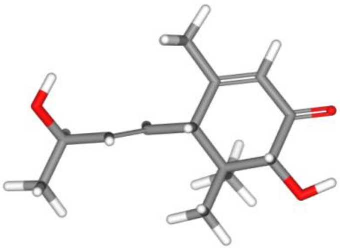	1	-733.1133991	-460035.6	27.09
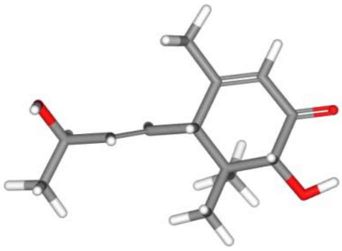	3	-733.1121396	-460034.81	7.14

Table.S 50 Standard orientations at mPW1PW91/6-311G(2d,p) level in Methanol. of **4 (2*R*,6*R*,9*R*)**

Conformer 11					
Center	Atomic	Atomic	Coordinates (Angstroms)		
Number	Number	Type	X	Y	Z
1	6	0	-0.5488	1.5942	-0.1122
2	6	0	-0.0439	0.192	-0.4631
3	6	0	-1.0429	-0.945	-0.0765
4	6	0	-2.4407	-0.5108	-0.5564
5	6	0	-2.857	0.8021	0.077
6	6	0	-1.8321	1.8505	0.1285
7	8	0	-3.3938	-1.4913	-0.3127

8	6	0	-1.0697	-1.2115	1.4386
9	6	0	-0.6664	-2.2432	-0.8038
10	6	0	1.3444	-0.042	0.0941
11	6	0	2.4051	-0.3322	-0.6351
12	6	0	3.8001	-0.5626	-0.1011
13	8	0	3.8386	-0.657	1.2984
14	6	0	4.743	0.5677	-0.4943
15	8	0	-3.9822	0.9581	0.4623
16	6	0	0.4641	2.7099	-0.1067
17	1	0	0.0423	0.1816	-1.5499
18	1	0	-2.3803	-0.318	-1.6303
19	1	0	-2.1765	2.8399	0.3706
20	1	0	-4.1821	-1.0549	-0.0045
21	1	0	-1.2175	-0.3057	2.0177
22	1	0	-0.1428	-1.6671	1.7685
23	1	0	-1.875	-1.8952	1.6733
24	1	0	0.319	-2.5844	-0.5087
25	1	0	-0.6635	-2.1041	-1.8816
26	1	0	-1.3818	-3.0216	-0.5716
27	1	0	1.4587	0.0494	1.1605
28	1	0	2.3116	-0.4129	-1.708
29	1	0	4.1756	-1.4876	-0.539
30	1	0	3.3624	-1.4283	1.575
31	1	0	5.7453	0.3543	-0.1416
32	1	0	4.4096	1.4978	-0.0481
33	1	0	4.7728	0.6905	-1.5721
34	1	0	1.0387	2.7053	-1.0281
35	1	0	-0.0203	3.6728	-0.0055
36	1	0	1.1731	2.5944	0.7058

Conformer 5

Center Number	Atomic Number	Atomic Type	Coordinates (Angstroms) X	Y	Z
1	6	0	-0.5715	1.5839	-0.165
2	6	0	-0.0497	0.1706	-0.4372
3	6	0	-1.0504	-0.9537	-0.0187
4	6	0	-2.4385	-0.554	-0.5542
5	6	0	-2.879	0.785	0.0033
6	6	0	-1.8618	1.8426	0.0296
7	8	0	-3.3918	-1.5281	-0.2875
8	6	0	-0.6464	-2.2831	-0.671
9	6	0	-1.1121	-1.1455	1.5067
10	6	0	1.3268	-0.0336	0.1597
11	6	0	2.4018	-0.3549	-0.5345
12	6	0	3.7814	-0.5817	0.0413
13	8	0	3.8627	-0.2454	1.401
14	6	0	4.8524	0.1465	-0.7695
15	8	0	-4.0141	0.953	0.3524
16	6	0	0.433	2.7072	-0.1854
17	1	0	0.0601	0.11	-1.5202
18	1	0	-2.3521	-0.413	-1.6345

19	1	0	-2.2187	2.84	0.2147
20	1	0	-4.1898	-1.0837	-0.0187
21	1	0	-0.6126	-2.1963	-1.7539
22	1	0	0.3306	-2.6039	-0.3295
23	1	0	-1.3653	-3.0531	-0.4223
24	1	0	-0.1897	-1.5742	1.8811
25	1	0	-1.2842	-0.2138	2.0362
26	1	0	-1.9172	-1.825	1.7545
27	1	0	1.4138	0.0789	1.2264
28	1	0	2.3253	-0.4908	-1.6031
29	1	0	3.9919	-1.6453	0.0069
30	1	0	3.7807	0.6948	1.4947
31	1	0	4.6826	1.2206	-0.7547
32	1	0	5.8325	-0.052	-0.3515
33	1	0	4.8482	-0.1757	-1.8059
34	1	0	1.1117	2.6423	0.6586
35	1	0	1.041	2.6581	-1.0837
36	1	0	-0.0602	3.6702	-0.1528

Conformer 1

Center Number	Atomic Number	Atomic Type	Coordinates (Angstroms) X	Y	Z
1	6	0	-0.7446	1.6409	-0.1096
2	6	0	-0.0773	0.2864	-0.3613
3	6	0	-0.9789	-0.9337	0.0122
4	6	0	-2.3787	-0.6661	-0.573
5	6	0	-2.9672	0.6192	-0.0257
6	6	0	-2.06	1.7712	0.039
7	8	0	-3.2408	-1.7307	-0.3453
8	6	0	-0.4237	-2.2116	-0.6319
9	6	0	-1.0796	-1.1444	1.5331
10	6	0	1.2862	0.2179	0.2934
11	6	0	2.4211	0.0011	-0.3449
12	6	0	3.7743	-0.0827	0.3136
13	8	0	4.6549	0.8639	-0.248
14	6	0	4.4243	-1.444	0.1167
15	8	0	-4.1244	0.6726	0.2845
16	6	0	0.1458	2.857	-0.0972
17	1	0	0.0872	0.242	-1.438
18	1	0	-2.2669	-0.5089	-1.6486
19	1	0	-2.5192	2.7282	0.2107
20	1	0	-4.0887	-1.3689	-0.107
21	1	0	0.5681	-2.4382	-0.2589
22	1	0	-0.3612	-2.1129	-1.7123
23	1	0	-1.0714	-3.0506	-0.4122
24	1	0	-1.3576	-0.2378	2.0612
25	1	0	-0.1358	-1.4894	1.94
26	1	0	-1.8258	-1.8985	1.7463
27	1	0	1.3091	0.3536	1.3636
28	1	0	2.4254	-0.1298	-1.4169
29	1	0	3.6617	0.1052	1.3799

30	1	0	4.2961	1.7332	-0.1328
31	1	0	5.4077	-1.4524	0.5725
32	1	0	3.8217	-2.2258	0.566
33	1	0	4.5392	-1.6567	-0.9408
34	1	0	0.8029	2.8553	0.7661
35	1	0	-0.4401	3.767	-0.0781
36	1	0	0.7806	2.8716	-0.9782

Conformer 3

Center	Atomic	Atomic	Coordinates (Angstroms)		
Number	Number	Type	X	Y	Z
1	6	0	-0.7411	1.6401	-0.1048
2	6	0	-0.0795	0.2832	-0.3595
3	6	0	-0.9854	-0.9344	0.0101
4	6	0	-2.3834	-0.6602	-0.5758
5	6	0	-2.9677	0.6262	-0.026
6	6	0	-2.0565	1.7741	0.0436
7	8	0	-3.25	-1.722	-0.3519
8	6	0	-0.4337	-2.2125	-0.6363
9	6	0	-1.0881	-1.1485	1.5303
10	6	0	1.283	0.212	0.2964
11	6	0	2.4194	0.0103	-0.3417
12	6	0	3.7746	-0.0658	0.3159
13	8	0	4.627	0.9473	-0.1679
14	6	0	4.4152	-1.4407	0.1455
15	8	0	-4.1257	0.6819	0.2817
16	6	0	0.1557	2.8511	-0.0881
17	1	0	0.0852	0.2413	-1.4363
18	1	0	-2.2699	-0.5008	-1.6509
19	1	0	-2.512	2.7323	0.2187
20	1	0	-4.0959	-1.3566	-0.1119
21	1	0	-1.0826	-3.0508	-0.4176
22	1	0	0.5581	-2.4413	-0.2643
23	1	0	-0.372	-2.1123	-1.7168
24	1	0	-1.8374	-1.9003	1.7412
25	1	0	-0.1459	-1.4977	1.9372
26	1	0	-1.3629	-0.242	2.0601
27	1	0	1.3053	0.3448	1.3665
28	1	0	2.4137	-0.1171	-1.4158
29	1	0	3.6647	0.1448	1.3719
30	1	0	4.8516	0.7699	-1.0717
31	1	0	3.8029	-2.2145	0.5959
32	1	0	4.5358	-1.684	-0.9081
33	1	0	5.3948	-1.4544	0.6101
34	1	0	-0.4261	3.7636	-0.0612
35	1	0	0.819	2.8405	0.7697
36	1	0	0.7883	2.8677	-0.9704

Functional	Solvent?	Basis Set	Type of Data
mPW1PW91	PCM	6-311G(d,p)	Unscaled Shifts

Nuclei	sp2?	xperimenta	Isomer 1	Isomer 2	Isomer 3	Isomer 4	Isomer 5
		DP4+	0.00%	0.24%	99.76%	0.00%	-
C		42.8	50.3	48.3	48.4	50.4	
C		77.4	82.8	78.9	78.6	82.8	
C	x	200.9	206.5	206.7	206.9	206.5	
C	x	125	128.3	127.9	129.0	128.4	
C	x	164.7	179.2	178.9	178.7	178.9	
C		58.6	60.0	61.5	60.2	60.1	
C	x	128.3	133.3	134.1	134.4	133.7	
C	x	138.5	149.5	148.6	146.2	149.2	
C		77	73.5	73.4	74.0	73.5	
C		23.7	27.3	26.0	26.4	27.2	
C		25.5	26.1	23.1	23.9	26.1	
C		20.1	14.14	23.37	22.26	14.15	
C		21	24.56	24.54	25.90	24.64	
H		4.17	4.09	4.35	4.33	4.09	
H	x	5.91	6.36	6.25	6.27	6.37	
H		2.77	3.17	2.89	2.93	3.13	
H	x	5.77	5.89	5.95	6.03	5.87	
H	x	5.76	6.15	6.18	6.12	6.00	
H		4.4	4.44	4.39	4.38	4.44	
H		1.92	1.90	2.08	2.15	2.10	
H		1.11	0.97	1.20	1.28	1.03	
H		0.9	0.69	0.64	0.91	0.69	
H		1.3	1.01	0.94	0.91	1.42	

Fig.S 57 Results of DP4+ Analysis (Isomer 1 is **2*S*,6*S*,9*R***; Isomer 2 is **2*S*,6*R*,9*S*;** Isomer 3 is **2*R*,6*S*,9*S*** ;Isomer 4 is **2*R*,6*R*,9*R***)

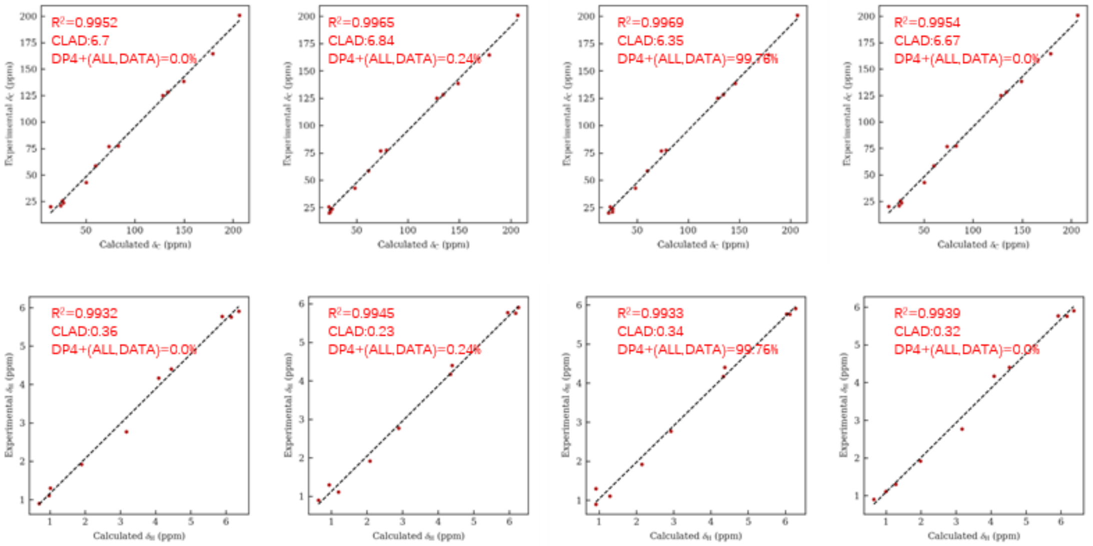

Fig.S 58 Linear regression equation for NMR calculation

(a)(e) is **2*S*,6*S*,9*R***; (b)(f) is **2*S*,6*R*,9*S*;** (c)(g) is **2*R*,6*S*,9*S*** ;(d)(h) is **2*R*,6*R*,9*R***

ECD calculation of 4

Table.S 51 Energy of structure for ECD calculation

conformer	Energy (Hartree)	Energy (kcal/mol)	Population (%)
1	-733.2730127	-460135.76	76.35
2	-733.271887	-460135.05	23.17
3	-733.2682216	-460132.75	0.48

Table.S 52 Standard orientations at B3LYP/6-311G(d,p) level in Methanol.

Conformer 1					
Center	Atomic	Atomic	Coordinates (Angstroms)		
Number	Number	Type	X	Y	Z
1	6	0	-1.1986	0.6585	1.5841
2	6	0	-0.4582	-0.1804	0.5396
3	6	0	-1.2186	-0.2603	-0.8327
4	6	0	-2.7033	-0.6116	-0.5711
5	6	0	-3.3489	0.3585	0.4134
6	6	0	-2.5148	0.9212	1.5001
7	8	0	-2.8356	-1.936	-0.0556
8	6	0	-0.5885	-1.3601	-1.7211
9	6	0	-1.1557	1.0697	-1.6267
10	6	0	0.9756	0.293	0.3625
11	6	0	2.053	-0.4921	0.5345
12	6	0	3.4692	-0.0345	0.3561
13	8	0	4.1658	-0.2992	1.5665
14	6	0	4.16	-0.7785	-0.7765
15	8	0	-4.5549	0.6019	0.3906
16	6	0	-0.4276	1.1267	2.7924
17	1	0	-0.4233	-1.1965	0.9619
18	1	0	-3.2649	-0.5634	-1.5124
19	1	0	-3.0498	1.5004	2.2446
20	1	0	-3.7932	-2.0966	0.0444
21	1	0	-1.1795	-1.5129	-2.6315
22	1	0	-0.5318	-2.3201	-1.1965
23	1	0	0.4271	-1.0957	-2.035
24	1	0	-0.1368	1.2956	-1.9593
25	1	0	-1.5078	1.9233	-1.0393
26	1	0	-1.7762	1.0134	-2.529
27	1	0	1.1174	1.3348	0.0796
28	1	0	1.9212	-1.5365	0.8133
29	1	0	3.5219	1.0438	0.1684
30	1	0	3.6564	0.1186	2.2815
31	1	0	3.6596	-0.6037	-1.7346
32	1	0	4.1853	-1.8581	-0.588
33	1	0	5.2025	-0.4533	-0.8647
34	1	0	0.3089	1.8875	2.516
35	1	0	0.0903	0.2847	3.2635
36	1	0	-1.0827	1.5692	3.5512

Conformer 2

Center Number	Atomic Number	Atomic Type	Coordinates (Angstroms) X	Y	Z
1	6	0	-1.0957	0.651	1.478
2	6	0	-0.4511	-0.2584	0.4291
3	6	0	-1.2801	-0.3362	-0.9026
4	6	0	-2.7638	-0.6135	-0.5594
5	6	0	-3.3172	0.4029	0.4348
6	6	0	-2.4025	0.9672	1.4538
7	8	0	-2.9291	-1.9199	-0.0075
8	6	0	-0.744	-1.4838	-1.7918
9	6	0	-1.1984	0.9705	-1.7328
10	6	0	0.9978	0.126	0.1736
11	6	0	2.0398	-0.7031	0.3638
12	6	0	3.4982	-0.4003	0.1478
13	8	0	3.9677	-1.2234	-0.9155
14	6	0	3.8528	1.0419	-0.1924
15	8	0	-4.5122	0.6947	0.4687
16	6	0	-0.2388	1.1358	2.6201
17	1	0	-0.4535	-1.2619	0.8818
18	1	0	-3.3698	-0.5593	-1.4725
19	1	0	-2.8708	1.598	2.2012
20	1	0	-3.8871	-2.0342	0.1407
21	1	0	-1.3856	-1.6307	-2.6684
22	1	0	-0.7039	-2.4329	-1.2462
23	1	0	0.2655	-1.273	-2.1609
24	1	0	-0.1883	1.1423	-2.1203
25	1	0	-1.4833	1.8529	-1.1517
26	1	0	-1.8649	0.9208	-2.6021
27	1	0	1.1717	1.1391	-0.182
28	1	0	1.8346	-1.7103	0.7238
29	1	0	4.0458	-0.6863	1.0531
30	1	0	3.6432	-2.1252	-0.7594
31	1	0	3.527	1.7334	0.591
32	1	0	3.4088	1.3485	-1.1463
33	1	0	4.9372	1.145	-0.3121
34	1	0	0.5119	1.851	2.2698
35	1	0	0.2691	0.2926	3.0999
36	1	0	-0.831	1.6387	3.3927

ECD calculation of 5

Table.S 71 Energy of structure for ECD calculation

conformer	Energy (Hartree)	Energy (kcal/mol)	Population (%)
1	-654.6705393	-410811.96	100

Table.S 72 Standard orientations at B3LYP/6-311G(d,p) level in Methanol.

Conformer 1					
Center	Atomic	Atomic	Coordinates (Angstroms)		
Number	Number	Type	X	Y	Z
1	6	0	0.4318	-0.8771	-0.0064
2	6	0	1.396	0.2426	-0.2424
3	6	0	1.0788	1.4188	0.6821
4	6	0	-0.397	1.8162	0.6038
5	6	0	-1.317	0.6269	0.9159
6	6	0	-1.064	-0.6253	0.0366
7	6	0	1.0987	-1.9924	0.3
8	6	0	2.52	-1.6551	0.3457
9	8	0	2.6653	-0.3225	0.121
10	6	0	-1.836	-1.8085	0.6652
11	6	0	-1.6365	-0.4194	-1.3782
12	8	0	3.4153	-2.4411	0.592
13	6	0	1.53	0.6731	-1.7064
14	8	0	-0.6635	2.8415	1.5584
15	1	0	1.7175	2.2802	0.4468
16	1	0	1.3383	1.17	1.7213
17	1	0	-0.6357	2.2264	-0.3826
18	1	0	-2.3637	0.9494	0.8312
19	1	0	-1.1885	0.3668	1.9772
20	1	0	0.7304	-2.9649	0.5739
21	1	0	-2.906	-1.5823	0.7446
22	1	0	-1.4765	-2.0346	1.676
23	1	0	-1.7382	-2.7183	0.0614
24	1	0	-1.3057	0.5137	-1.8383
25	1	0	-2.7321	-0.3794	-1.3554
26	1	0	-1.3511	-1.2414	-2.045
27	1	0	1.6541	-0.1953	-2.3643
28	1	0	2.4272	1.2892	-1.8413
29	1	0	0.6778	1.2535	-2.0647
30	1	0	-0.0696	3.5892	1.372

ECD calculation of 6

Table.S 73 Energy of structure for ECD calculation

conformer	Energy (Hartree)	Energy (kcal/mol)	Population (%)
1	-654.6710175	-410812.26	99.6
2	-654.665819	-410809	0.4

Table.S 74 Standard orientations at B3LYP/6-311G(d,p) level in Methanol.

Conformer 1					
Center	Atomic	Atomic	Coordinates (Angstroms)		
Number	Number	Type	X	Y	Z
1	6	0	0.3566	0.6554	0.1124
2	6	0	0.5624	-0.8674	0.1953
3	6	0	-0.1981	-1.5739	-0.9268
4	6	0	-1.6459	-1.0827	-1.037
5	6	0	-1.8038	0.4457	-1.0461
6	6	0	-1.0478	1.2042	0.0828
7	6	0	1.5417	1.2616	-0.0446
8	6	0	2.6195	0.2395	-0.1501
9	8	0	2.011	-1.0269	-0.0872
10	6	0	-1.0413	2.7074	-0.2474
11	6	0	-1.7267	0.9878	1.4429
12	8	0	3.8065	0.3118	-0.2962
13	6	0	0.3462	-1.4634	1.5715
14	8	0	-2.322	-1.6777	0.094
15	1	0	-0.1946	-2.6717	-0.7491
16	1	0	0.3347	-1.4287	-1.8886
17	1	0	-2.1221	-1.5113	-1.9524
18	1	0	-2.8766	0.7102	-0.9954
19	1	0	-1.4526	0.8246	-2.028
20	1	0	1.7778	2.3092	-0.1232
21	1	0	-0.5369	3.2844	0.5371
22	1	0	-0.5255	2.9202	-1.1903
23	1	0	-2.0588	3.1037	-0.3329
24	1	0	-1.8705	-0.0855	1.6496
25	1	0	-1.1288	1.4034	2.2615
26	1	0	-2.7107	1.4646	1.4827
27	1	0	0.7321	-0.8211	2.3705
28	1	0	0.8661	-2.429	1.6588
29	1	0	-0.7278	-1.6462	1.7515
30	1	0	-3.246	-1.3704	0.1459

Acid hydrolysis of compounds 3-5,6a and 6b

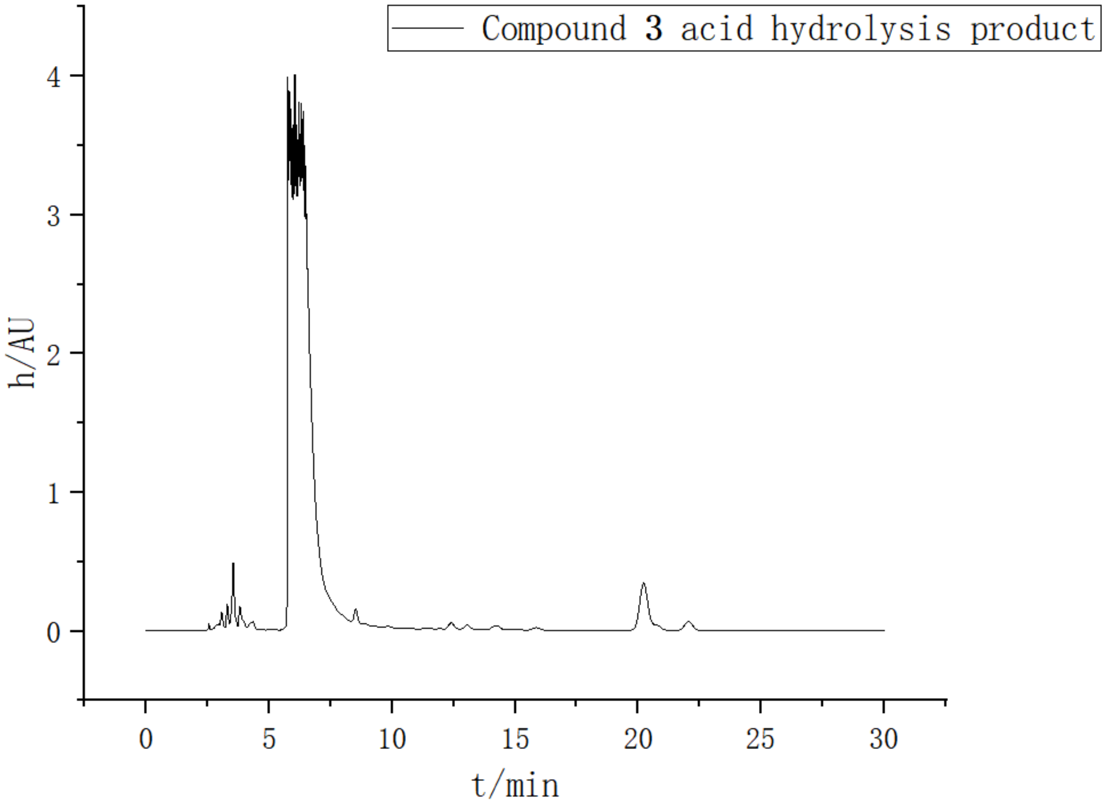

Fig.S 59 Acid hydrolysate of compound **3**

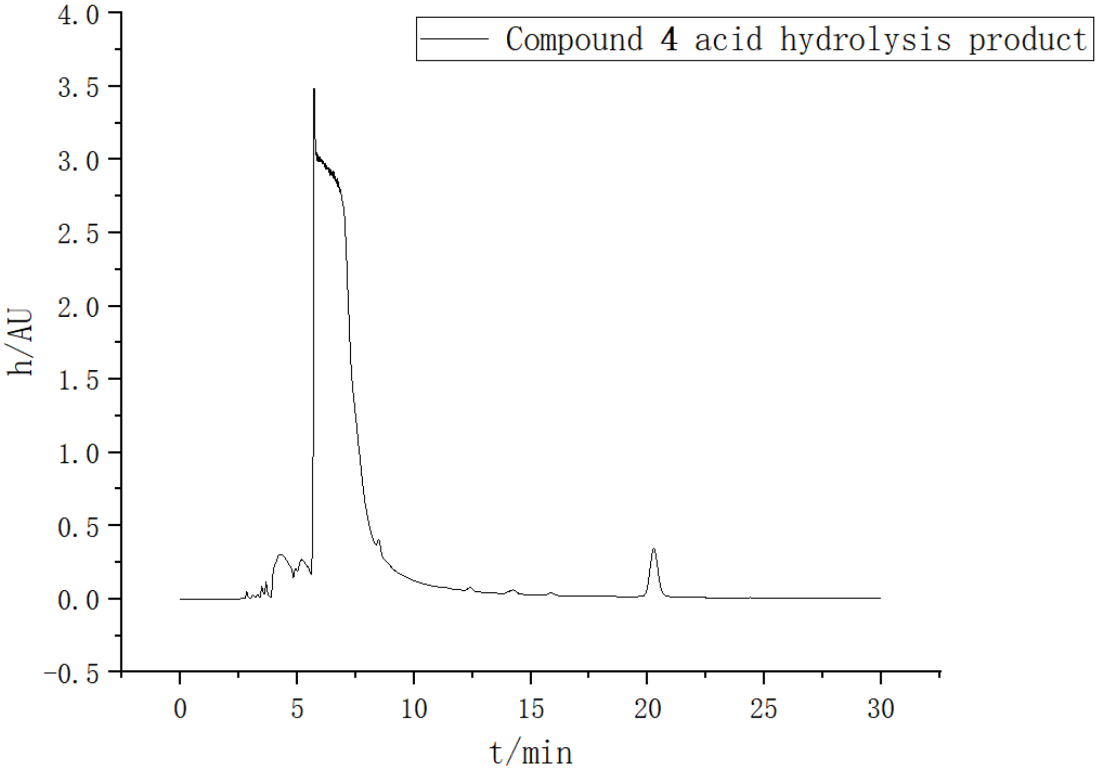

Fig.S 60 Acid hydrolysate of compound **4**

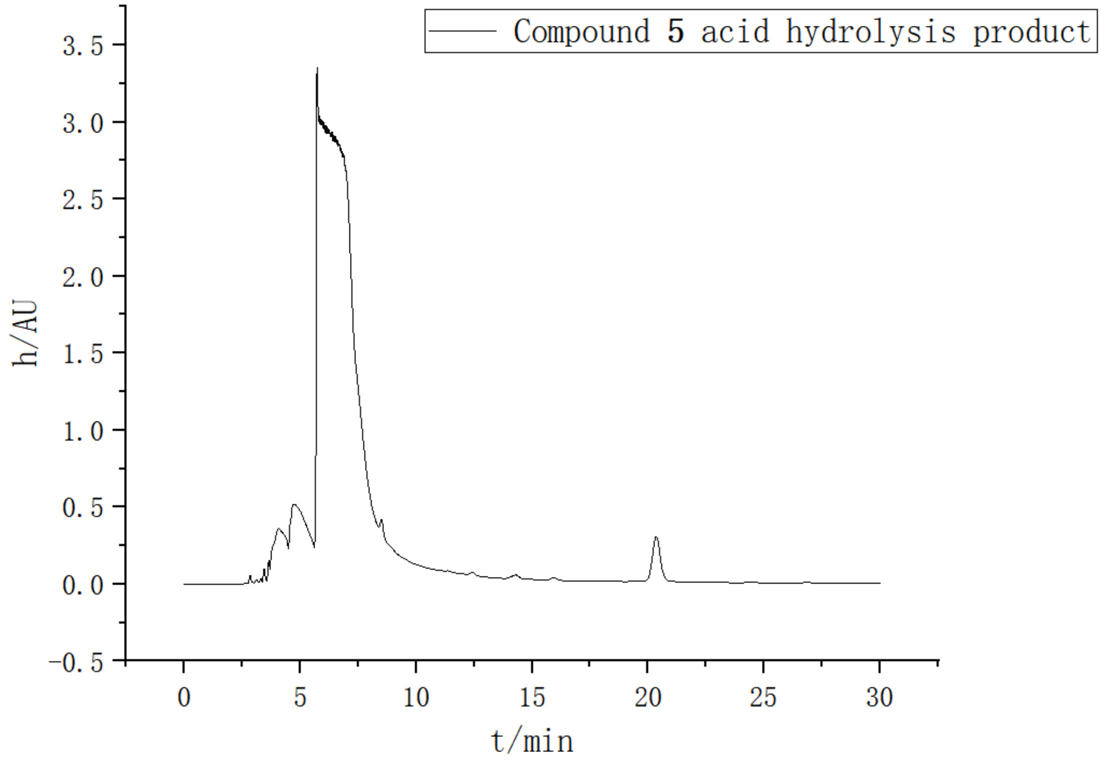

Fig.S 61 Acid hydrolysate of compound **5**

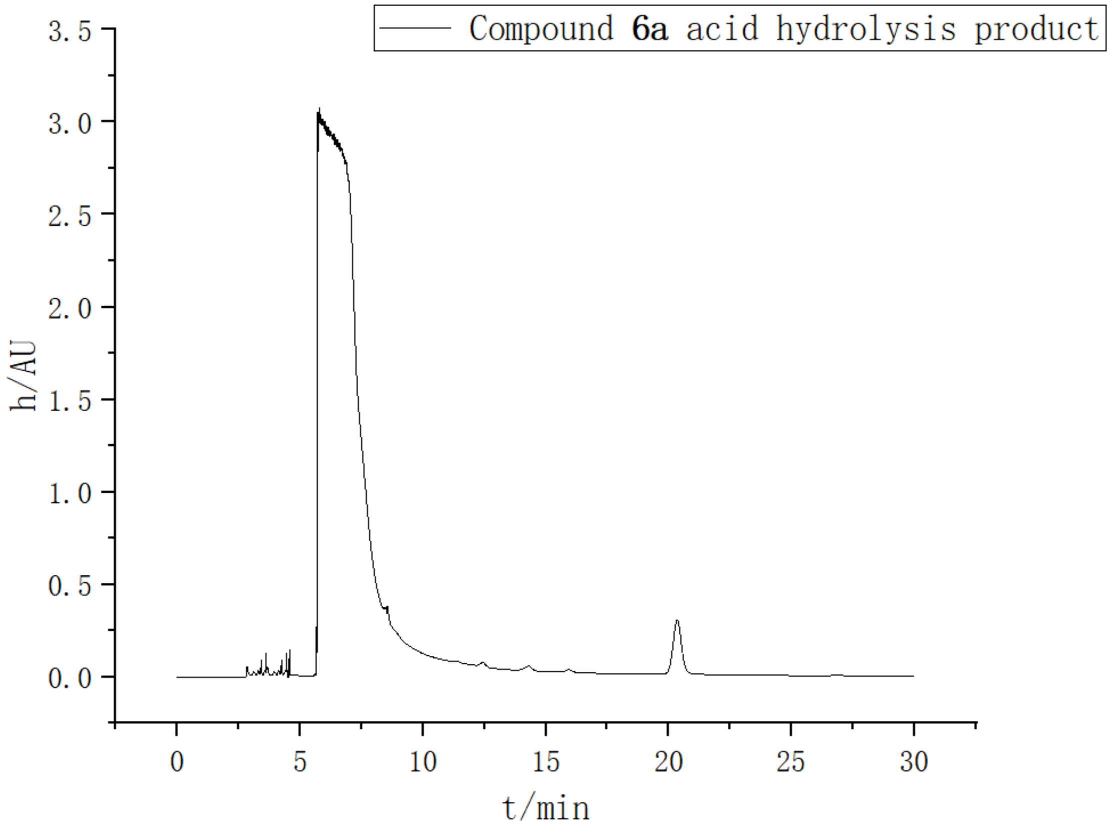

Fig.S 62 Acid hydrolysate of compound **6a**

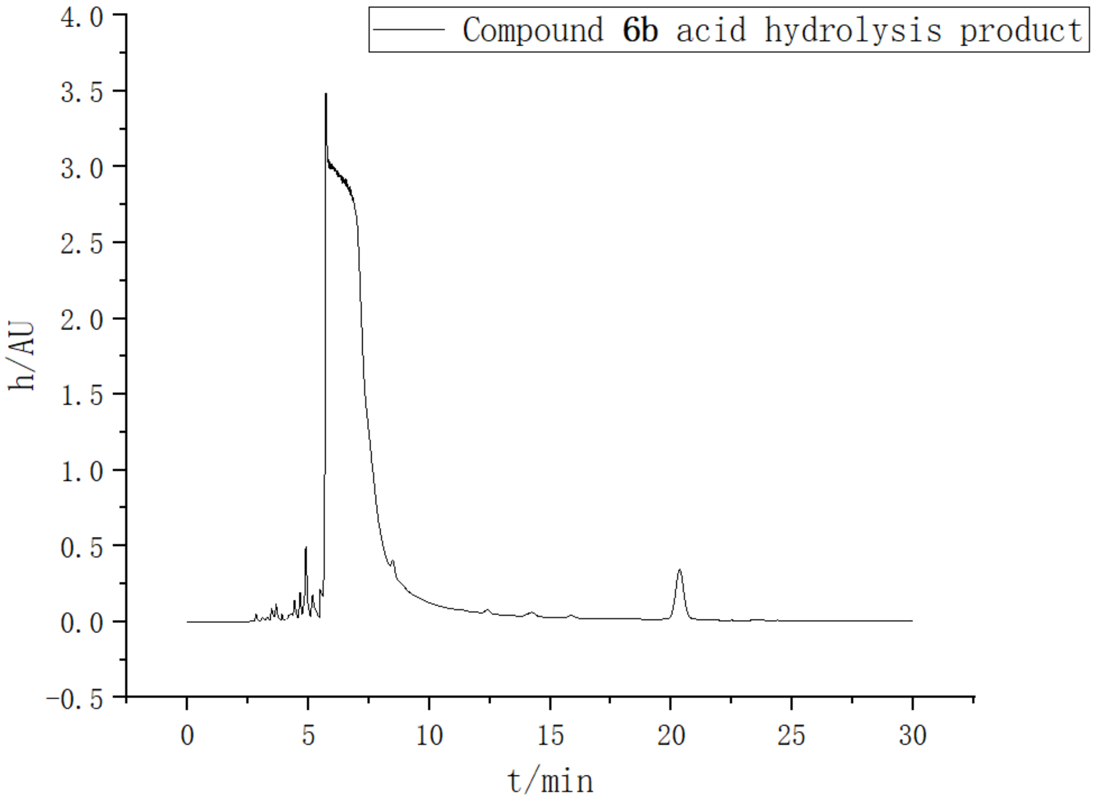

Fig.S 63 Acid hydrolysate of compound **6b**

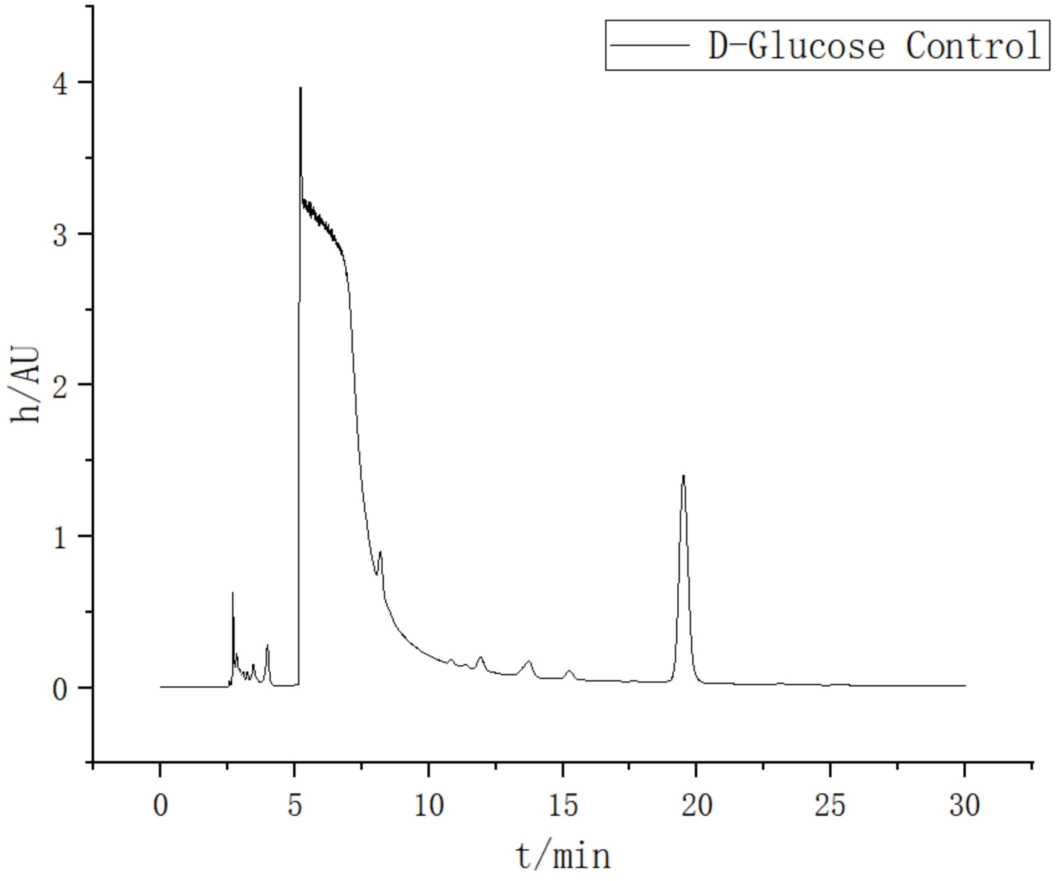

Fig.S 64 Acid hydrolysate of glucose control

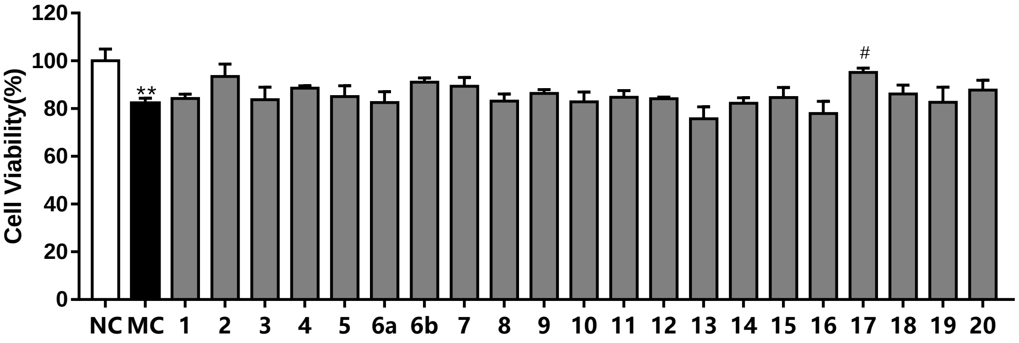

Fig.S 65 The ability of compounds **1-20** against H/R-induced neonatal rat cardiomyocytes injury.

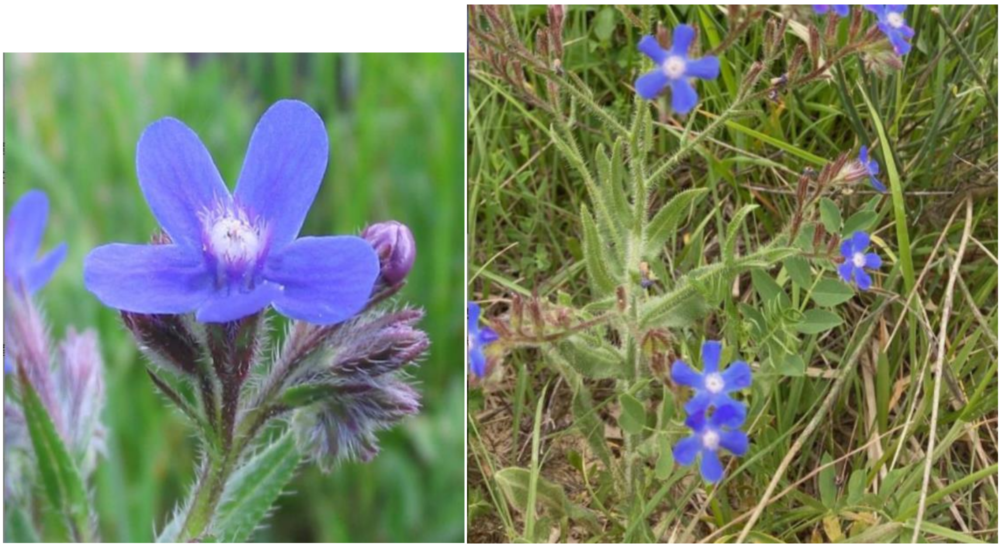

Figure S66 Photographs of *Anchusa italica* Retz.

The feature of *Anchusa italica* Retz. is that there are many small prickles on the leaves and stems, so it is metaphorically called the grass of ox tongue.
